# Murine Myocardial Transcriptome Analysis Reveals a Critical Role of COPS8 in the Gene Expression of Cullin-RING Ligase Substrate Receptors and Redox and Vesicle Trafficking Pathways

**DOI:** 10.3389/fphys.2017.00594

**Published:** 2017-08-17

**Authors:** Ammara Abdullah, Kathleen M. Eyster, Travis Bjordahl, Peng Xiao, Erliang Zeng, Xuejun Wang

**Affiliations:** ^1^Division of Basic Biomedical Sciences, Sanford School of Medicine of the University of South Dakota Vermillion, SD, United States; ^2^Department of Computer Science and Department of Biology, University of South Dakota Vermillion, SD, United States

**Keywords:** the COP9 signalosome, transcription profiling, myocardium, COPS8 gene, conditional gene targeting, cullin-RING ligases, F-box proteins, mouse

## Abstract

**Background:** The COP9 signalosome (CSN) consisting of 8 unique protein subunits (COPS1 through COPS8) serves as the cullin deneddylase, regulating the catalytic dynamics of cullin RING ligases (CRLs), the largest family of ubiquitin ligases Background: The COP9 signalosome (CSN) consisting of 8 unique protein subunits (COPS1 through COPS8) serves as the cullin deneddylase, regulating the catalytic dynamics of cullin RING ligases (CRLs), the largest family of ubiquitin ligases. Supported primarily by the decrease of substrate receptor (SR) proteins of CRLs in cells deficient of a CSN subunit, CSN-mediated cullin deneddylation is believed to prevent autoubiquitination and self-destruction of the SR in active CRLs. However, it is unclear whether the decrease in SRs is solely due to protein destabilization. Moreover, our prior studies have demonstrated that cardiac specific knockout of *Cops8* (Cops8-CKO) impairs autophagosome maturation and causes massive necrosis in cardiomyocytes but the underlying mechanism remains poorly understood. Given that Cops8 is nucleus-enriched and a prior report showed its binding to the promoter of several genes and association of its ablation with decreased mRNA levels of these genes, we sought to determine the dynamic changes of myocardial transcriptome in mice with perinatal Cops8-CKO and to explore their functional implications.

**Methods and Results:** Myocardial transcriptomes of *Cops8*^*flox*/*flox*^, *Cops8*^*flox*/+^*::Myh6-Cre*, and *Cops8*^*flox*/*flox*^*::Myh6-Cre* littermate mice at postnatal 2 and 3 weeks were analyzed. The data were imported into an in-house analysis pipeline using Bioconductor for quantile normalization and statistical analysis. Differentially expressed genes (DEGs) between groups at each time point or between time points within the group were revealed by *t*-test. Genes with *p* < 0.05 after Benjamini and Hochberg false discovery rate correction for multiple hypothesis testing were considered as significant DEGs. We found that (1) the Ingenuity Pathway Analysis (IPA) revealed significant enrichment of DEGs in multiple pathways, especially those responding to oxidative stress, in homozygous Cops8-CKO hearts at both 2 and 3 weeks, corroborating the occurrence of massive cardiomyocyte necrosis at 3 weeks; (2) the decreases in multiple CRL SR proteins were associated with decreased transcript levels; and (3) enrichment of DEGs in the chromatin remodeling pathway and the microtubule motility and vesicle trafficking pathways.

**Conclusions:** Our data are consistent with the notion that Cops8/CSN plays a role in the transcriptional regulation of CRL SRs and in the redox and vesicle trafficking pathways.

## Introduction

Originally identified in mutant *Arabidopsis* that exhibits constitutive photomorphogenesis (Wei and Deng, 1992), the COP9 signalosome (CSN) is evolutionally conserved from yeast to humans, with its *bona fide* biochemical activity being cullin deneddylation responsible for the deconjugation of the ubiquitin-like protein NEDD8 from the cullin scaffold subunit of cullin-RING ligases (CRLs) (Lyapina et al., 2001; Bosu and Kipreos, 2008; Sozen et al., 2015). The assembly of a CRL is centered on a cullin scaffold which binds a substrate receptor (SR) module at its amino terminal end and a catalytic RING-containing protein (either RBX1 or RBX2) at its carboxyl terminal end. Covalent attachment of NEDD8 to the cullin via a ubiquitination-like process known as neddylation activates the CRL while CSN-mediated cullin deneddylation is also required for the proper functioning of most, if not all, CRLs (Wei and Deng, 2003; Chamovitz, 2009). CRLs are the largest family of ubiquitin E3 ligases; hence, the CSN plays an important role in regulation of ubiquitination. In higher eukaryotes, the CSN holocomplex consists of 8 unique protein subunits (CSN1 through CSN8) (Wei and Deng, 1999) where the deneddylase enzymatic activity resides in the metalloenzymatic motif or JAMM domain of CSN5 subunit (Cope et al., 2002). CSN1-CSN4, CSN7, and CSN8 are the six PCI (Proteasome lid-CSN-*I*nitiation factor 3) domain-containing proteins whereas CSN5 and CSN6 are the two MPN (MPR1/PAD1 amino-terminal) domain-containing proteins. The crystal structure of the CSN holoenzyme reveals that the 6 PCI's form a horseshoe-shaped ring where α-helices of all subunits form a large bundle. CSN5 and CSN6 are present at the core of this bundle. In the absence of substrate, CSN5 is auto-inhibited. CSN4 senses the binding of neddylated CRL whereas CSN5 with the help of CSN6, specifically removes NEDD8 from CRLs (Lingaraju et al., 2014). Binding of either the CSN or a CRL substrate to a neddylated CRL is a competitive mechanism. According to the current model, when the substrate for an active CRL runs low, the CSN binding and subsequent deneddylation of CRLs prevents the autoubiquitination and resultant destruction of the SR proteins of the CRL complex (Wang and Martin, 2015). On the other hand, binding of substrate to CRL complexes markedly inhibits deneddylation by the CSN. In fact, binding of substrate to the CSN-CRL complex facilitates dissociation of the CSN (Emberley et al., 2012). Dissociation of the CSN from the unloaded and deneddylated CRL allows binding by CAND1 (Cullin-Associated and Neddylation-Dissociated 1) protein. The CAND1 dissociates the old SR module for a new SR, which allows formation of a new CRL that is specific for a different class of substrates. The preserved SRs can also complex with the cullin and RBX-assembly of no longer needed CRLs and maintain homeostasis of CRL substrates in the cell (Schmidt et al., 2009; Pierce et al., 2013; Wang and Martin, 2015). Given the indispensable role of the CSN in propelling cell division cycle progression and sustaining cell survival, targeting the CSN for treatment of cancer has long been sought and, with the identification of small molecular inhibitors of CSN5 (Schlierf et al., 2016), this strategy has shown great promise; hence, it becomes more significant to have a better understanding of the impact of CSN impairment on the wellbeing of vital organs, such as the heart.

Ablation of any one of the CSN subunits leads to loss of the holocomplex and cullin deneddylation activity (Busch et al., 2007). Therefore, the entire CSN holocomplex is required for cullin deneddylation although the deneddylase activity of the CSN only resides in CSN5 (Wei and Deng, 2003). The CSN8, the smallest and the least conserved CSN subunit, is encoded by the *COPS8* gene. We have previously reported that CSN8 is required for the deneddylation of cullins in cardiomyocytes and that a defect in deneddylation activity due to conditional knockout of the *Cops8* gene in cardiomyocytes (CSN8-CKO) leads to decreased expression of SR proteins, such as Fbxo32, VHL and Fbxw1a, in the heart (Su et al., 2011b). Our group has also provided compelling evidence that CSN8/CSN regulates not only the ubiquitin-proteasome system (UPS) but also the autophagic-lysosomal pathway for cardiac protein quality control and that CSN8-CKO initiated in perinatal and adult mice causes massive cardiomyocyte necrosis and heart failure (Su et al., 2011a,b, 2013). However, the molecular basis underlying these CSN8/CSN functions in the heart remains poorly understood.

Most studies have defined CSN8/CSN as a post-translational regulator of protein degradation. CSN8 function at the transcriptional level has rarely been investigated in vertebrates although the CSN was originally identified as a transcriptional repressor (Wei and Deng, 1992; Wei et al., 1994). CSN subunits have been evaluated for their role beyond the regulation of protein stability and it was suggested that CSN subunits might have a direct role in the regulation of gene expression (Wei et al., 2008; Chamovitz, 2009). In human HEK293 cells, silencing of CSN5- caused a decrease in F-box protein 4 (*Fbx4*) expression (Cope and Deshaies, 2006). In mice, CSN5-deleted thymocytes showed decreased expression of *I*κ*B*-α mRNA (Panattoni et al., 2008). Similarly, CSN8 conditional knockout in peripheral T cells caused aberrant gene expression of several cell cycle genes and chromatin immunoprecipitation assays detected CSN8 at the promoter regions of these genes (Menon et al., 2007). We have previously shown that CSN8 is enriched in the nuclei of cardiomyocytes (Su et al., 2011b), consistent with a role of CSN8 in regulation of cardiac gene expression. However, the role of CSN8 as a potential transcriptional regulator in the heart has not been tested. We therefore sought to determine the impact of cardiac CSN8 deficiency on myocardial gene expression. The impact of loss of a CSN subunit on the full transcriptome of an organ in a vertebrate animal has not been described although the gene expression profile of ablation of CSN subunits, such as CSN4 and CSN5 in lower species (e.g., *Drosophila*) were reported (Oron et al., 2007).

Using whole-genome DNA microarray, here we profiled the dynamic changes in myocardial transcriptome of mice with perinatal CSN8-CKO. Our analyses demonstrate for the first time that CSN8-CKO has a major effect on the transcript levels of genes enriched in multiple pathways including signaling pathways in responses to oxidative stress, microtubule dynamics and vesicle trafficking, corroborating very well with the functional changes in the heart. Further verification of the expression changes at the protein level reveals that the reduced protein levels of many SR proteins of CRLs in myocardium with CSN8-CKO are associated with decreased expression at the transcript level, suggesting that CSN8/CSN plays an important role in promoting the gene expression of the SR in addition to protecting them from autoubiquitination.

## Materials and methods

### Mice

We have previously described generation of CSN8-CKO mice with the use of the *Cre*-loxP system in which transgenic *Cre* expression is driven by the mouse α-myosin heavy chain (*Myh6*) promoter (Su et al., 2011b). The *Cops8*^*flox*/*flox*^ (CTL) and *Cops8*^*flox*/+^*::Myh6-Cre* (Heterozygous *Cops8* knockout or Het-CKO) littermates of *Cops8*^*flox*/*flox*^*::Myh6-Cre* (homozygous CSN8-CKO or Hom-CKO) mice were used. The knockout of the *Cops8* gene which encodes CSN8 was confirmed with real time reverse transcription PCR (RT^2^-PCR) where primers for the 5'UTR region and exon 5 of the *Cops8* gene were used (Supplementary Figures 1A,B). This study was carried out in accordance with the recommendations of the Guide for the Care and Use of Laboratory Animals (US Department of Health, Education, and Welfare, Department of Health and Human Services, NIH Publication 85-23). The protocol for care and use of animals in this study was approved by the Institutional Animal Care and Use Committee of the University of South Dakota.

### RNA extraction and purification

Total RNA from mouse ventricular myocardial samples was extracted and purified using the RNeasy plus mini kit (Catalog number: 74134) from Qiagen. Briefly, 30–50 mg of ventricular myocardial tissue was homogenized in 1 mL of TRI reagent with a Polytron homogenizer. Bromochloropropane (200 μL) and 3 M sodium acetate (60 μL) were added and the homogenate was centrifuged. After centrifugation the aqueous layer was collected, mixed with RLT buffer (Qiagen, Valencia, CA) and ethanol, and centrifuged through a Qiagen RNeasy column. The column was washed and treated with ribonuclease-free deoxyribonuclease (Qiagen) to remove any potentially contaminating DNA. The total RNA was then eluted from the column.

### RNA quality control and quantification

RNA samples were tested for their quality and quantity by microfluidics analysis using a RNA 6000 Nano LabChip kit in an Agilent Bioanalyzer (Agilent Technologies).

### Microarray gene expression analysis

Gene expression in the myocardium of CTL, Het-CKO, and Hom-CKO littermate mice at 2 and 3 weeks of age (*n* = 3 mice/group, both sexes were used for each group) was analyzed using DNA microarrays (CodeLink Whole Mouse Genome Bioarrays, Applied Microarrays, Inc., Tempe, AZ) as described previously (Eyster et al., 2007). Briefly, the MessageAmp II aRNA Amplification kit (Ambion/ThermoFisher) was used for the synthesis of biotinylated cRNA. RNA in the samples was reverse transcribed into first strand complementary DNA (cDNA) and the second strand of cDNA was then synthesized. Complementary RNA (cRNA) was synthesized from the double-stranded cDNA in a reaction that incorporates biotin-11-UTP. The biotinylated cRNA was purified on a Qiagen RNeasy column and fragmented. The fragmented cRNA was hybridized with the DNA microarrays for 18 h at 37°C. The slides were washed and incubated with streptavidin-Alexa Fluor 647 (Molecular Probes) and washed again. The slides were scanned with an Axon GenePix 4,000B Scanner. Gene expression values were obtained from a summarization of intensity values of all corresponding probes using the RMA (Robust Multi-array Average) method. The pre-processed microarray data were imported into an in-house analysis pipeline using Bioconductor for quantile normalization and statistical analysis.

The choice of the 2- and 3-week-of-age time points was based on the phenotype of these mice. Overt left heart failure as reflected by increased lung weight to body weight ratios was not discerned at 3 weeks but was evidenced at 4 weeks in the Hom-CKO mice; cardiomyocyte necrosis as well as the impairment of UPS performance as reflected by accumulation of a surrogate UPS substrate were not discernible until 3 weeks of age (Su et al., 2011a,b). Hence, to decipher potential molecular basis at the transcription level for CSN8 deficiency to induce cardiomyocyte necrosis and myocardial UPS impairment we elected to examine a time point before (2 weeks) and a time point after (3 weeks) these major pathologies become detectable in the Hom-CKO mice.

### Pathway and network analysis

Differentially expressed genes (DEGs) were analyzed using the QIAGEN's Ingenuity Pathway Analysis (IPA, QIAGEN Redwood City, www.qiagen.com/ingenuity) to identify significantly enriched pathways. Expression data were processed by the IPA software suite system, which scores and ranks pathways enriched in our data by the proportion of pathway associated genes with significant expression values. Ultimately, this allows the visualization of canonical pathways significantly enriched for our given data set. Network analysis was performed using R package WGCNA on the expression profiles of all hand-curated pathway genes. The package WGCNA uses a topological overlap measure to construct weighted correlation networks. We applied it in this study to detect gene correlation modules, which are defined as groups of highly correlated genes. The network obtained was exported to Cytoscape, a network visualization software platform.

### Real time reverse transcriptase PCR (RT^2^-PCR)

RT^2^-PCR was used to confirm the gene expression of *Cops8* and additional genes in mouse heart tissue. Predesigned primers and probes for 5′UTR and exon 5 region of the CSN8 gene as well as for Synapsin II were obtained from Assays on Demand (Applied Biosystems). TaqMan Gold RT-PCR reagents were used to perform RT-PCR (Applied Biosystems, Foster City, CA). While the first-strand cDNA synthesis was carried out using a MultiScribe Reverse Transcriptase, AmpliTaq Gold DNA polymerase was used for DNA amplification. Triplicates of each RNA sample were performed. For control reactions, a no-reverse transcriptase (NRT) and a no-RNA control (NRC) for each experimental and reference gene (GAPDH) were included. Each well contained appropriate Master Mix pool or No-RT Master Mix pool (19 μl) and appropriate RNA (6 μl). Plate was sealed with a 96-well plate film and centrifuged at 1,200 × g for 2 min. Plate was then placed in StepOne Plus Real Time PCR Machine and program was run according to manufacturer's instruction. Changes in expression of each gene of interest were calculated relative to the endogenous control (housekeeping gene) GAPDH. An RNA concentration response curve was obtained for all genes including GAPDH. The validation curves were used to determine the concentration of RNA added to the RT-PCR reaction. Data from the RT^2^-PCR reactions were analyzed with use of qBase software (http://medgen.ugent.be/qbase/). This program uses a delta Ct (threshold cycle) relative quantitation model with PCR efficiency correction and multiple reference gene normalization.

### Western blot analysis

Ventricular myocardial tissues from Hom-CKO mice as well as littermate controls were collected and immediately frozen in liquid nitrogen. Samples were then stored at –80°C until further use. Tissues were homogenized using a lysis buffer (80 mM Tris-HCl, 2% SDS and 10% glycerol) and further sonicated on ice. The tissue lysates were boiled for 5 min and then centrifuged at 12,000 g for 8 min. The supernatant was collected for western blot analyses. The concentration of total protein was determined with BCA reagents (Pierce, Rockford, IL, USA). To probe for FBXO31, 60 μg of total proteins, and to probe for Asb-14 and TTLL1, 100 μg of total proteins were separated by SDS-PAGE, transferred to PVDF membrane, and blotted with rabbit anti-FBXO31 antibody (1:500, Abcam), Anti-Asb14 antibody (1:200, Santa Cruz), and anti-TTLL1 antibody (Santa Cruz, 1:1,000). Western blot analysis was performed as described previously (Su et al., 2011a).

### Statistical analysis

The microarray data were imported into an in-house analysis pipeline using Bioconductor for quantile normalization and statistical analysis. DEGs between groups at each time point or between time points within the group were revealed by *t*-test. Genes with *p* < 0.05 after Benjamini and Hochberg false discovery rate correction for multiple hypothesis testing were considered as significant DEGs. The enrichment analysis of hand-curated pathway genes in significant DEGs was performed using Fisher's exact test. The *p*-value (< a cutoff value 0.05) obtained represents enrichment of pathway genes in the DEG set. The comparison of RT^2^-PCR and microarray data of individual gene expression among CTL, Het-CKO, Hom-CKO groups used one way ANOVA followed by Tukey's test for pair-wise comparison. Western blot data were analyzed with *t*-test using GraphPad Prism (GraphPad Software, San Diego, CA).

## Results

### Identification of DEGs in response to CSN8-CKO

Whole mouse genome DNA microarrays (CodeLink) were utilized to compare the gene expression profiles among control (CSN8^flox/flox^, CTL), heterozygous CSN8-CKO (Het-CKO), and homozygous CSN8-CKO (Hom-CKO) mouse groups. The raw data for these DNA microarrays have been deposited in the National Center for Biotechnology Information Gene Expression Omnibus (NCBI GEO; www.ncbi.nlm.nih.gov/geo, Accession Number GSE100104) in accordance with Minimum Information About a Microarray Experiment (MIAME) standards (Brazma et al., 2001).

GeneSpring GS software (v.12.5) was initially used to analyze DEGs. The microarray data revealed 521 DEGs at 3-weeks-of-age with cut-off log2 |fold change|≧1. Among these genes, 271 genes were upregulated and 250 were downregulated. Similarly, at 2-weeks-of-age, out of 78 DEG, 35 genes were upregulated and 43 were downregulated.

The Hom-CKO mice start displaying cardiac hypertrophy and cardiac malfunction at 2 weeks of age and overt heart failure at 4 weeks (Su et al., 2011b). One of the characteristics of cardiac hypertrophy or injury is reactivation of the fetal gene program. Using multiple methods, our group had previously shown the induction of fetal genes natriuretic peptide type A (*Nppa*) and B (*Nppb*), skeletal α-actin (*Acta1*), and β-myosin heavy chain (*Myh7*) in the Hom-CKO hearts (Su et al., 2011b). In the present study, gene expression profiling also detected marked reactivation of the fetal gene program (Supplementary Figure 2A), which is consistent with cardiac pathology in the Hom-CKO mice and, more importantly here, provides a verification of the sensitivity as well as the accuracy of our microarray data. Down-regulation of calcium handling genes (e.g., *Pln, Atp2a2*) and adult forms of sarcomeric genes (e.g., *Actc1* and *Tpm1*) is another gene expression signature of cardiac hypertrophy and failure. Our microarray data also revealed significant down-regulation of these genes in Hom-CKO mouse hearts at 3 weeks (Supplementary Figure 2B), which is again consistent with known phenotype of Hom-CKO mice and once again attests the sensitivity and the accuracy of our microarray data.

Prolonged *Cre* transgene expression driven by the *Myh6* promoter could have a significant impact on cardiac gene expression (Pugach et al., 2015), Het-CKO mice are therefore a better control group for the Hom-CKO mice than the CTL group in terms of controlling for the potential off-target effects of the transgenic *Cre*, given that both Het-CKO and Hom-CKO harbor *Myh6-Cre* and importantly that myocardial CSN8 protein levels are not discernibly altered in the Het-CKO mice compared with the littermate CTL (Supplementary Figure 1C) and wild type mice. Thus, the DEGs from the comparison between Het-CKO and Hom-CKO reflect conservatively the impact of CSN8 deficiency on gene expression, thereby constituting the main stream data for our elucidation of their functional implication. From the Hom-CKO vs. Het-CKO comparison, the top under- or over-expressed DEGs with respect to their fold change values along with *p*-values are listed in Tables 1, 2, respectively. The maximum down-regulation is by 77 and 91% at 2 and 3 weeks, respectively (Table 1), whereas the maximum up-regulation is 2.44 and 7.57 fold at 2 and 3 weeks, respectively (Table 2). For the most part, alterations of these DEGs are more pronounced at 3-weeks compared with 2-weeks, which is further unveiled by comparisons between the 3-week and the 2-week time points within each of the 3 genotypes. With rare exception, the expression levels of these top down- or up-regulated genes were comparable between 3- and 2-weeks-of-age within either CTL or Het-CKO genotypes (Tables 1, 2); however, the transcript levels of approximately 30% of the top down-regulated genes (Table 1) and 70% of the top up-regulated genes (Table 2) in the Hom-CKO mice differed significantly between the 3-week and 2-week time points.

**Table 1:** List of top down-regulated DEGs in Hom-CKO vs. Het-CKO and their temporal changes within each genotype.

				Hom-CKO/Het-CKO^*^				**3-Week/2-Week^§^**					
				**2-Week**		**3-Week**		**Control**		**Het-CKO**		**Hom-CKO**	
**No**.	**ACCN#**	**Gene**	**Description**	**Fold**	***p***	**Fold**	***p***	**Fold**	***p***	**Fold**	***p***	**Fold**	***p***
1	BE994427.1	Syp	Synaptophysin	0.23	0.001	0.16	0.030	0.97	NS	1.06	NS	0.71	NS
2	BG797117.1	Phkg1	Phosphorylase kinase gamma 1	0.27	0.005	0.24	0.009	1.59	NS	1.24	NS	1.11	NS
3	NM_009700.1	Aqp4	aquaporin 4	0.29	0.005	0.21	0.019	0.84	NS	0.79	NS	0.56	0.043
4	NM_178869.2	Ttll1	tubulin tyrosine ligase-like 1, mRNA	0.30	0.001	0.22	0.008	1.94	NS	1.73	NS	1.26	NS
5	X98848.1	Pfkfb1	6-phosphofructo-2-kinase/fructose-2,6-biphosphatase 1	0.35	0.010	0.19	0.008	1.64	NS	1.16	NS	0.62	NS
6	NM_029555.1	Gstk1	glutathione S-transferase kappa 1	0.37	0.004	0.29	0.009	3.83	NS	2.88	0.043	2.24	0.030
7	NM_172778.1	Maob	monoamine oxidase B	0.39	0.009	0.20	0.003	2.35	NS	1.87	NS	0.97	NS
8	NM_177591.3	Igsf1	immunoglobulin superfamily, member 1, transcript variant 1	0.40	0.007	0.32	0.021	1.66	NS	1.19	NS	0.96	NS
9	NM_133664.2	Lad1	ladinin	0.41	0.009	0.48	0.030	1.02	NS	1.12	NS	1.32	NS
10	NM_009405.1	Tnni2	troponin I, skeletal, fast 2	0.44	0.008	0.30	0.027	1.43	NS	1.39	NS	0.95	NS
11	CA451887.1	Cryba4	Crystallin, beta A4	0.44	0.009	0.26	0.024	1.36	NS	1.28	NS	0.76	NS
12	R75121.1	Kcnd2	Potassium voltage-gated channel, shaker-related, subfamily, member 2	0.45	0.012	0.32	0.016	1.17	NS	1.10	NS	0.77	NS
13	AK081465.1	Zfp72	zinc finger protein 72	0.46	0.010	0.40	0.013	1.02	NS	1.02	NS	0.89	NS
14	NM_007994.1	Fbp2	fructose bisphosphatase 2	0.47	0.007	0.17	0.020	0.67	NS	0.48	NS	0.18	0.016
15	NM_146292.1	Olfr1324	olfactory receptor 1324	0.49	0.006	0.42	0.020	1.44	NS	1.00	NS	0.85	NS
16	NM_182839.1	Tppp	Tubulin polymerization promoting protein	0.50	0.006	0.42	0.014	0.76	NS	0.99	NS	0.83	NS
17	AB055885.1	Lrg1	Leucine-rich alpha-2-glycoprotein 1	0.50	0.010	0.34	0.030	1.74	NS	1.40	NS	0.96	NS
18	NM_145158.2	Emilin2	elastin microfibril interfacer 2	0.51	0.003	0.48	0.012	0.77	NS	0.82	NS	0.78	0.046
19	NM_053200.1	Ces3	carboxylesterase 3	0.25	NA	0.09	0.016	6.33	0.044	4.80	NS	1.66	NS
20	NM_080462.1	Hnmt	histamine N-methyltransferase	0.49	NA	0.20	0.012	1.55	NS	1.81	NS	0.72	NS
21	NM_009464.2	Ucp3	uncoupling protein 3, mitochondrial	0.79	NA	0.21	0.030	0.37	NS	0.41	NS	0.11	0.021
22	NM_028247.1	Slc16a10	solute carrier family 16 (monocarboxylic acid transporters), member 10	0.48	NA	0.22	0.030	1.66	NS	1.37	NS	0.69	0.026
23	NM_172303.3	Phf17	PHD finger protein 17	0.91	NA	0.23	0.006	1.09	NS	1.00	NS	0.25	0.011
24	AK017136.1	Ptchd3	Patched domain containing 3	0.52	NA	0.28	0.029	1.46	NS	1.44	NS	0.78	NS
25	AK014194.1	Rhobtb1	Rho-related BTB domain containing 1	0.55	NA	0.30	0.008	1.35	NS	1.44	NS	0.80	NS
26	NM_013912.2	Apln	apelin	0.89	NA	0.31	0.028	0.72	NS	0.59	NS	0.20	0.019
27	NM_025599.1	Cmss1	CMS small, ribosomal subunit 1	0.56	NA	0.31	0.013	1.64	NS	1.71	NS	0.94	NS
28	NM_177725.2	Lrrc8	leucine-rich repeat-containing 8	0.62	NA	0.32	0.010	1.80	NS	1.67	NS	0.86	NS
29	NM_183283.1	Smco1	Single pass membrane protein with coiled coil domains 1	0.53	NA	0.32	0.007	2.13	NS	1.94	NS	1.20	NS
30	AK033571.1	Myo5c	Myosin VC	0.52	NA	0.33	0.030	0.80	NS	0.93	NS	0.59	0.052

* *In the Hom-CKO vs. Het-CKO comparisons, p-vales were derived from one way ANOVA followed by Tukey's test where other two pair-wise comparisons (Hom-CKO vs. CTL and Het-CKO vs. CTL) were not shown; NA, not available as statistical significance was not shown by ANOVA*.

§ *For comparisons between the 3- and. 2-week time points within each genotype, p-values were derived from t-test; NS, not significant. The footnote remains the same for Table 2*.

**Table 2:** List of top up-regulated DEGs in Hom-CKO vs. Het-CKO and their temporal changes within each genotype.

				Hom-CKO/Het-CKO^*^				**3-Week/2-Week^§^**					
				**2-Week**		**3-Week**		**Control**		**Het-CKO**		**Hom-CKO**	
**No**.	**ACCN#**	**Gene**	**Description**	**Folds**	***p***	**Folds**	***p***	**Folds**	***p***	**Folds**	***p***	**Folds**	***p***
1	NM_153744.1	Prkag3	protein kinase, AMP-activated, gamma 3 non-catatlytic subunit	2.44	0.006	4.16	0.010	1.20	NS	1.01	NS	1.72	NS
2	NM_173385.1	Cilp	cartilage intermediate layer protein, nucleotide pyrophosphohydrolase	2.44	0.013	4.18	0.020	0.63	NS	0.82	NS	1.41	NS
3	BQ176278.1	Grm1	Glutamate Receptor,metabotropic 1	2.44	0.009	2.38	0.030	1.66	NS	1.74	NS	1.70	NS
4	NM_183294.1	Cdkl1	cyclin-dependent kinase-like 1 (CDC2-related kinase)	2.43	0.007	3.98	0.005	1.00	NS	0.88	NS	1.44	0.031
5	BC052064.1	Parp6	Poly (ADP-ribose)polymerase family, member 6	2.39	0.002	3.72	0.007	1.09	NS	1.13	NS	1.76	0.026
6	BI412344.1	Mettl21d	Methyltransferase like 21D	2.37	0.005	3.05	0.030	1.02	NS	1.34	NS	1.72	0.031
7	NM_021467.4	Tnni1	troponin I, skeletal, slow 1	2.31	0.022	1.53	NA	0.11	NS	0.36	NS	0.24	0.043
8	NM_007669.2	Cdkn1a	cyclin-dependent kinase inhibitor 1A (P21)	2.27	0.007	6.34	0.005	0.87	NS	1.25	NS	3.50	0.025
9	NM_019875.1	Abcb9	ATP-binding cassette, sub-family B (MDR/TAP), member 9	2.25	0.004	3.61	0.030	1.11	NS	0.93	NS	1.49	0.046
10	NM_016867.1	Semcap2	semaF cytoplasmic domain associated protein 2	2.22	0.007	4.87	0.007	1.21	NS	1.42	0.044	3.11	0.027
11	NM_013468.2	Ankrd1	ankyrin repeat domain 1 (cardiac muscle)	3.53	0.119	7.57	0.011	1.45	NS	1.06	NS	2.28	NS
12	NM_013681.1	Syn2	synapsin II	4.22	0.520	7.38	0.010	1.50	NS	1.12	NS	1.96	NS
13	NM_019687.2	Slc22a4	solute carrier family 22 (organic cation transporter), member 4	1.42	NA	6.48	0.013	3.37	NS	0.83	NS	3.77	0.016
14	CB848957.1	Auh	AU RNA binding protein/enoyl CoA hydratase	1.78	NA	6.25	0.004	1.03	NS	0.94	NS	3.31	0.014
15	AI853801.1	Srsf1	Serine-arginine-rich splicing factor 1	2.78	NA	6.21	0.002	1.25	NS	1.44	NS	3.23	0.015
16	NM_010077.1	Drd2	dopamine receptor 2	1.61	NA	6.05	0.012	1.22	NS	1.07	NS	4.01	0.041
17	AA536960.1	Rexo1	RNA exonuclease 1 homolog	1.10	NA	5.79	0.008	1.22	NS	1.08	NS	5.68	0.028
18	BC027359.1	Mphosph8	M phase phosphoprotein 8	2.72	0.002	5.45	0.008	0.76	NS	0.77	NS	1.54	NS
19	NM_011103.1	Prkcd	protein kinase C, delta	2.92	0.001	5.40	0.004	1.19	NS	1.11	NS	2.06	0.017
20	W83574.1	Ndufa3	NADH dehydrogenase (ubiquinone) 1 alpha subcomplex, 3	2.59	0.002	5.23	0.014	0.89	NS	0.94	NS	1.89	0.014
21	NM_011485.3	Star	steroidogenic acute regulatory protein	1.68	NA	5.15	0.030	1.16	NS	0.83	NS	2.56	NS
22	NM_145495.1	Rin1	Ras and Rab interactor 1	2.03	NA	4.96	0.005	1.46	NS	1.07	NS	2.60	0.026
23	NM_007474.1	Aqp8	aquaporin 8	2.07	NA	4.81	0.030	1.12	NS	1.14	NS	2.66	NS
24	NM_199222.1	Lman1l	Lectin, mannose-binding 1 like	1.42	NA	4.77	0.013	1.24	NS	1.16	NS	3.88	0.013
25	AI647986.1	Bhmt2	Betaine-homocysteine methyltransferase 2	2.72	0.004	4.60	0.006	0.91	NS	1.15	NS	1.94	0.044
26	BY457927.1	Rps3a1	Ribosomal protein S3a	1.21	NA	4.52	0.008	1.04	NS	1.12	NS	4.17	0.016
27	AK009352.1	Nmrk2	Nicotinamide riboside kinase 2	2.46	0.017	4.39	0.028	1.08	NS	1.25	NS	2.24	NS
28	NM_080640.3	Baalc	brain and acute leukemia, cytoplasmic	1.22	NA	4.38	0.030	1.15	NS	1.08	NS	3.87	0.037
29	NM_024286.1	Popdc3	popeye domain containing 3	1.91	NA	4.28	0.005	1.41	NS	1.00	NS	2.23	0.025
30	BE911434.1	Fam20a	Family with sequence similarity 20, member A	1.41	NA	4.23	0.003	1.00	NS	0.98	NS	2.93	0.019

**Figure 1:**
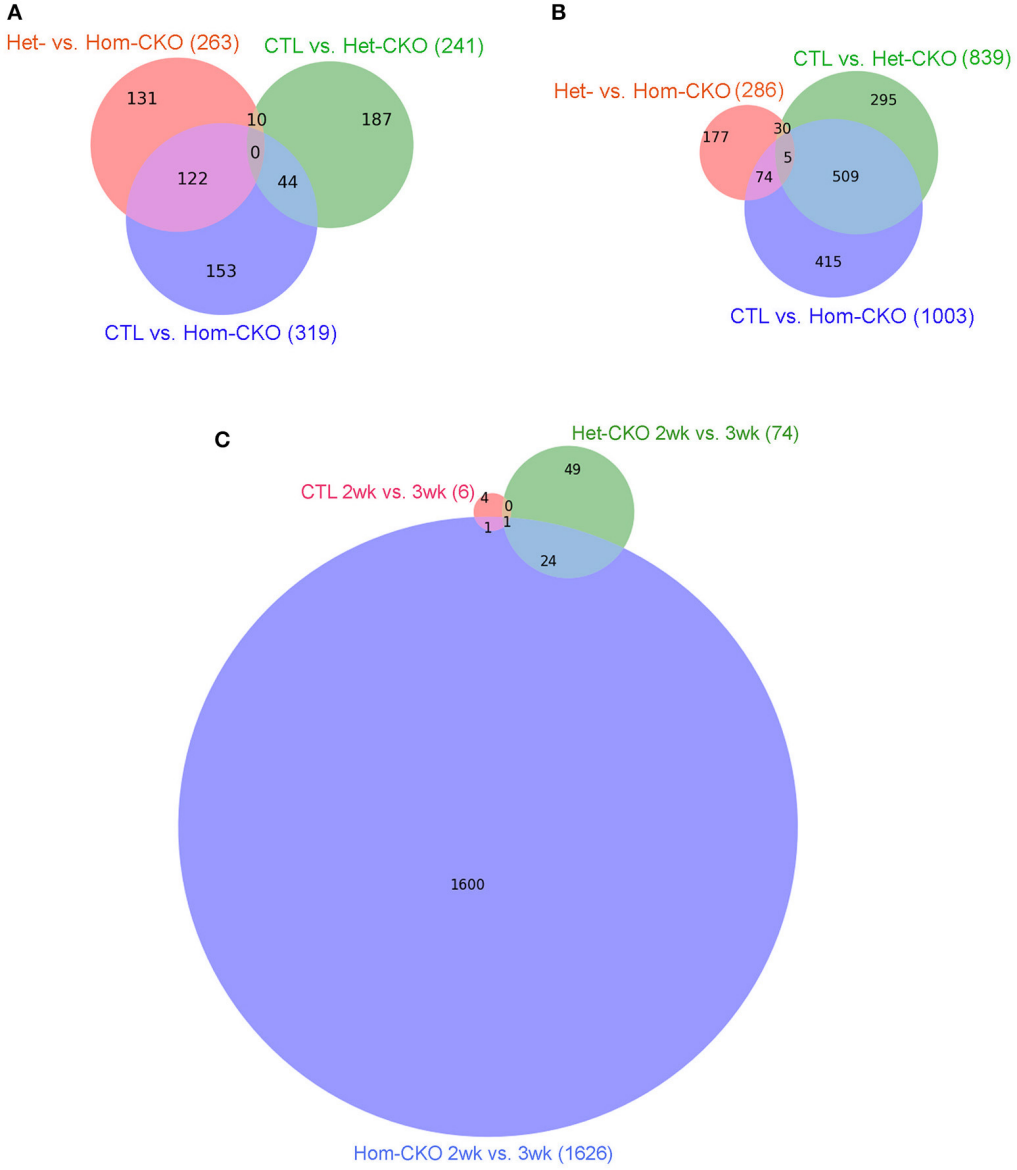
Venn diagrams showing the distribution of significant differentially-expressed genes (DEGs) that are unique or common among the three comparisons between genotypes: Het-CKO vs. Hom-CKO (Red), Control (CTL) vs. Hom-CKO (blue), CTL vs. Het-CKO (green) at postnatal 2 weeks **(A)** and 3 weeks **(B)**, or among the three comparisons between the 2-weeek (2wk) and 3-week (3wk) time points within each genotype **(C)**.

We also used intersection analysis (Venn diagram) to identify shared DEGs among the three comparisons: Het-CKO vs. Hom-CKO, CTL vs. Het-CKO, and CTL vs. Hom-CKO, at both 2 and 3 weeks of age (Figure 1). The analyses reveal that at 2-weeks-of-age (Figure 1A), 263, 241, and 319 genes had differential transcript expression for the three comparison pairs, respectively. These results indicate that 131 out of 263 DEGs were unique for Hom- vs. Het-CKO, 187 out of 241 were unique for Het-CKO vs. CTL, and 153 out of 319 were unique for Hom-CKO vs. CTL. There were 10 DEGs common between CTL vs. Het-CKO and Het- vs. Hom-CKO, 122 common between Het- vs. Hom-CKO and CTL vs. Hom-CKO, and 44 common between CTL vs. Hom-CKO and CTL vs. Het-CKO. We did not find any DEGs that were common among all three comparisons at 2 weeks.

The 3-weeks-of-age Venn-diagram shows 286, 839, and 1003 DEGs for the Het- vs. Hom-CKO, CTL vs. Het-CKO, and CTL vs. Hom-CKO comparisons, respectively (Figure 1B). These results indicate that 177 out of 286 were unique for Het- vs. Hom-CKO, 295 out of 839 DEGs were unique for CTL vs. Het-CKO, and 415 out of 1003 were unique for CTL vs. Hom-CKO. There were 30 DEGs common between CTL vs. Het-CKO and Het vs. Hom-CKO, 509 common between CTL vs. Het-CKO and CTL vs. Hom-CKO, and 74 common between CTL vs. Hom-CKO and Het vs. Hom-CKO. There were 5 DEGs that were common among all three comparisons at 3 weeks. Those genes included *cacna1s, ptgfr, mylk2 and ppp1r3g* whereas one of them was an unknown gene (Supplementary Table 1 and Supplementary Figure 3). Of the four known genes, the expression differentials of the *ptgfr* and *mylk2* genes between Hom-CKO and Het-CKO are more striking than the other two, and both are involved in cardiac muscle contraction. The *ptgfr* gene encodes the receptor for prostaglandin F_2_α (PGF_2_α). PGF_2_α is produced during myocardial inflammation as a cardiac response (Tracey, 2002). In isolated cardiomyocytes, PGF_2_α has been shown to increase intracellular Ca^2+^ and therefore ventricular contractility in response to inflammation (Ponicke et al., 2000; Takayama et al., 2005) by increasing inositol triphosphate (IP_3_) signaling pathway (Fabiato, 1992). Hence, the drastic down-regulation of *ptgfr* gene in Hom-CKO compared with both Het-CKO and CTL groups (Supplementary Figure 3B) may have contributed to CSN8-CKO-induced decrease in cardiac contractility. The *mylk2* gene encodes for myosin light chain kinase (MLCK) enzyme that phosphorylates myosin light chain (MLC2). The phosphorylation of MLC2 regulates contraction of smooth muscles (Murthy, 2006) and increases the myosin-actin cross-bridge formation in cardiomyocytes following increased intracellular Ca^2+^ concentration and thereby improves the cardiac contractility (Moss and Fitzsimons, 2006; Stelzer et al., 2006). Of interest, the expression of *mylk2* was modestly down-regulated in Het-CKO hearts but increased drastically in the Hom-CKO hearts (Supplementary Figure 3C), suggesting either that the down-regulation results from an off-target effect of Myh6-Cre or that the increased *mylk2* expression is a secondary effect. We have previously shown that CSN8-deficiency in cardiomyocytes leads to cardiac hypertrophy and heart failure (Su et al., 2011b, 2013), but the direct effect of CSN8 function on ventricular contractility was not assessed at the transcriptional level. Hence, this is the first time it is proposed that decreased transcription of *ptgfr* contributes to CSN8-CKO-induced decrease in cardiac contractility.

The findings that very few DEGs were common to all three pairwise comparisons (0 at 2 weeks and up to 5 at 3 weeks) also indicate that virtually none of the DEGs caused by loss of CSN8 proteins (Hom-CKO vs. Het-CKO) overlap with those caused potentially by the off-target effects of Cre (Het-CKO vs. CTL).

In addition, we performed longitudinal comparisons between the 2- and 3-week time points within each of the three genotypes, which identified 6, 74, and 1626 significant DEGs for the CTL, Het-CKO, and Hom-CKO mice, respectively. Among these DEGs, only 1 DEG is common to all 3 genotypes, 24 additional DEGs are shared between Het-CKO and Hom-CKO, 1 additional DEGs is common between CTL and Hom-CKO but no additional DEG common between CTL and Het-CKO (Figure 1C). From 2- to 3-week, very few significant DEGs were found in either CTL or Het-CKO mice while a large number of significant DEGs detected in Hom-CKO mice with only a very small proportion of them shared among different genotypes, indicating that the effect of perinatal CSN8 deficiency has a great impact on myocardial transcriptome independent of developmental changes during this critical period; this also suggests that the selection of this time window is physiologically appropriate for revealing the genomic effect of perinatal CSN8 deficiency.

### Functional enrichment analysis for hand-curated pathway genes

To determine the functional enrichment of certain pathways, we performed Fisher's exact test of hand-curated pathway genes. A pathway with a *p*-value below 0.05 was considered as a functionally enriched pathway. Based on our previous findings of CSN8 function, we analyzed CRL SRs (SOCS and F-box genes), autophagy, microtubule-related, vesicle trafficking, chromatin remodeling, endocytosis, ATP synthases, and cell death pathways. The results from the Hom-CKO vs. Het-CKO comparison showed that except the cell death pathway, all pathway genes were functionally enriched in response to CSN8 deficiency in mouse hearts at both 2 and 3 weeks (Table 3). The hand-curated individual genes for all of the above nine pathways are listed in Supplementary Tables 2–10.

**Table 3:** The *p*-value of hand-curated pathway genes tested with Fisher's Exact test.

**No**.	**Pathways**	**2 weeks**		**3 weeks**	
		**Hom-CKO vs. CTL**	**Hom-CKO vs. Het-CKO**	**Hom-CKO vs. CTL**	**Hom-CKO vs. Het-CKO**
1	SOCS-box	0.002256	2.24E-06	9.70E-07	1.36E-08
2	F-box	4.78E-09	1.53E-14	1.82E-09	1.20E-15
3	Autophagy	1.39E-11	2.20E-16	6.44E-16	2.20E-16
4	Microtubules-related	2.20E-16	2.20E-16	2.20E-16	2.20E-16
5	Vesicle trafficking	1.39E-11	2.20E-16	2.20E-16	2.20E-16
6	Chromatin Remodeling	6.90E-09	2.20E-16	2.20E-16	2.09E-15
7	Endocytosis	8.46E-06	2.20E-16	7.33E-15	1.84E-14
8	ATP synthases	0.1417	6.05E-06	4.04E-06	0.006758
9	Cell Death Pathway	1	1	1	1

### Hierarchical cluster analysis of DEGs between CTL and CSN8-CKO samples

**Figure 2:**
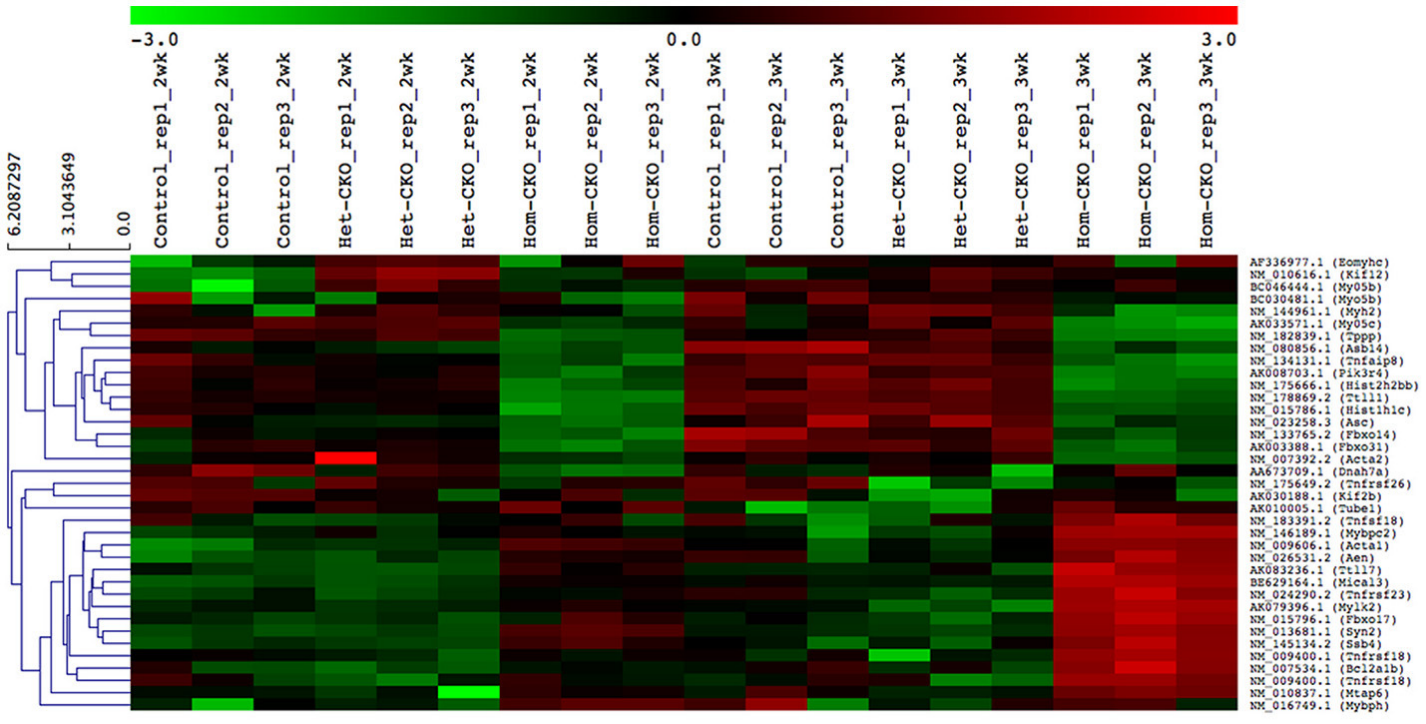
Heatmap/hierarchical clustering of DEGs of seven different pathways. All replicates of each group (Control, Het-CKO and Hom–CKO) are shown. Similarity between clusters is shown through dendrograms. Vertical dendrograms represent expression values from microarray data. Green and red indicate low and high levels of transcript abundance, respectively.

A heat map provides an immediate visual summary of experimental data since the individual values, represented by colors, indicate low and high levels of transcript abundances (Kalil and Florescu, 2013). In hierarchical clustering, subjects with similar features in a heatmap can be grouped as clusters (Zhang et al., 2017). For example, in terms of gene array, genes with similar expression can be clustered in the same group while similarity among clusters is shown by dendrograms. We used average linkage and Euclidean distance metric to generate hierarchical clustering. The results of the clustering analysis of all DEGs among CTL, Het- and Hom-CKO groups at 2- and 3-weeks-of-age are displayed in Supplementary Figure 4. The functional enrichment analysis for hand-curated genes determined DEGs within autophagy, CRL SRs (F-box and SOCS-box genes), cell death pathway, chromatin remodeling, vesicle trafficking, and microtubule-related pathway genes. Using those DEGs, we created a heat map to look at hierarchical clustering among pathways that are all associated to CSN8 function (Figure 2). The gene expression profile of Hom-CKO group was significantly different from CTL and Het-CKO group and DEGs from different pathways could be successfully clustered. Compared with CTL and Het-CKO group, we identified 18 hand-curated genes that were downregulated and 19 genes that were upregulated in Hom-CKO group. Heatmap/hierarchical clustering also showed that out of 37 DEGs, 18 were microtubule-related genes, 8 were cell-death pathway genes, three were F-box genes, two were SOCS-box genes, two genes for fetal gene program and chromatin remodeling, and a single gene for vesicular trafficking and autophagy pathways (Figure 2).

### Ingenuity pathway analysis

**Figure 3:**
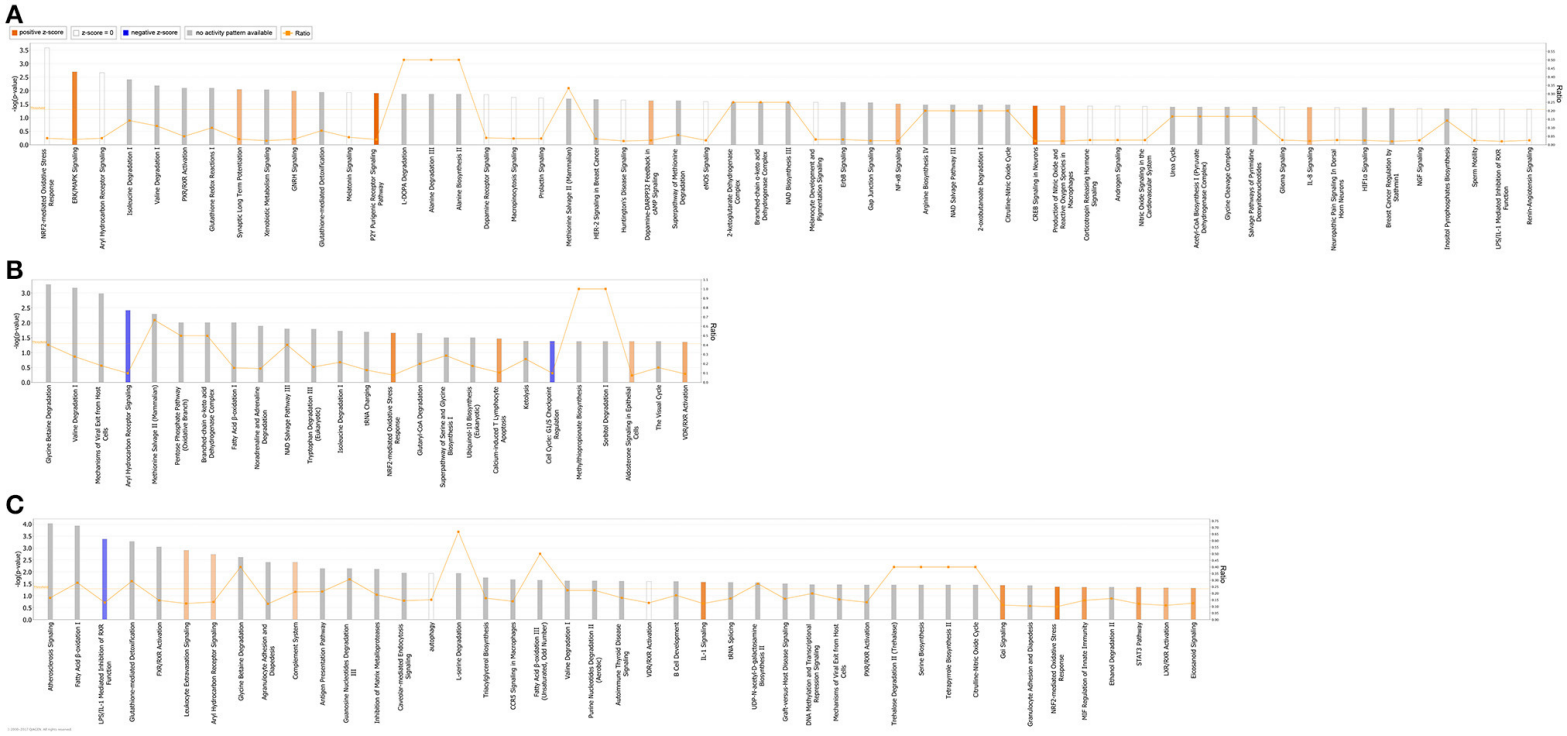
Significantly enriched (*p* < 0.05; Fisher's exact test) Ingenuity canonical pathways in Hom-CKO compared with Het-CKO at 2 weeks **(A)** and 3 weeks **(B)** of age, and in 3-week-old Hom-CKO compared with 2-week-old Hom-CKO **(C)**. The X-axis represents significant canonical pathways while the left Y-axis displays the -log of *p*-value which is calculated with Fisher's exact test right-tailed. Thus, taller bars equate to increased significance. The threshold is set at -log of 0.05 (*p*-value) so all values above or at that threshold denote significant enrichment. The overall activation/inhibition states of canonical pathways are predicted based on a Z-score algorithm. The canonical pathway bar charts are colored to indicate their activation z-scores. Orange bars predict an overall increase in the activity of the pathway while blue bars indicate a prediction of an overall decrease in activity. White bars are those with a z-score which is zero or very close to 0, predicting unchanged overall activity of the pathway. Gray bars indicate pathways that are currently ineligible for a prediction. The orange points connected by a thin line represent the Ratio (right Y-axis). The ratio is calculated as the number of genes in a given pathway that meet cutoff criteria, divided by the total number of genes that make up that pathway and that are in the reference gene set.

QIAGEN's IPA was employed to identify integral biological pathways, cellular processes and upstream regulators that are associated with the cardiac CSN8 deficiency. For reasons described earlier, the significant DEGs from the Het-CKO vs. Hom-CKO comparison best reflect the impact of CSN8 deficiency on gene expression. Thus, we will describe IPA results from this cohort of comparisons in greater detail here. IPA shows that at 2 weeks of age, DEGs in Hom-CKO compared with littermate Het-CKO are significantly enriched in a large number of pathways, some of which are predicted to have increased activity (i.e., positive z-score), including ERK/MAPK signaling, synaptic long term potentiation, GNRH signaling, P2Y purinergic receptor signaling pathway, dopamine-DARPP32 feedback in cAMP signaling, NF-κB signaling, CREB signaling, production of NO and ROS in macrophages, and IL-8 signaling (Figure 3A). Activation of these pathways likely plays a major role in mediating cardiac hypertrophy observed at this time point and/or the inflammatory responses and cell death that become apparent at a later time point (e.g., 3 weeks). Enhanced ERK/MAPK signaling is well-known to perturb cardiac development (Bezniakow et al., 2014) and to participate in both adaptive and maladaptive cardiac hypertrophy and remodeling (Rose et al., 2010). CREB is a transcription factor implicated in cardiac hypertrophy that can be activated by a variety of upstream signaling pathways including ERK/MAPK (Subedi et al., 2017). The involvement of purinergic signaling, including P2Y receptor-mediated signaling in cardiovascular pathophysiology, such as cardiac hypertrophy and heart failure, and its emergence as a therapeutic target are well-evidenced in recent literature (Burnstock, 2017). A meta-analysis of genome-wide association studies has revealed that dopamine-DARPP32 feedback in cAMP signaling is one of the signaling pathways shared by mood disorders and cardio-metabolic diseases (Amare et al., 2017). Meanwhile, NF-κB signaling, production of NO and ROS in macrophages, and IL-8 signaling are apparently involved in the regulation of inflammation and cell death (Valen, 2011). Both inflammation and cell death are clearly evidenced in Hom-CKO hearts at 3 weeks of age (Su et al., 2011a,b).

For the 3-week time point, IPA reveals that significant DEGs between Hom-CKO and Het-CKO are enriched in fewer pathways (Figure 3B) although the total number of DEGs is somewhat larger at 3-weeks (286) than at 2-weeks (263) (Figures 1A,B). Nevertheless, approximately one third of the enriched pathways at 3-weeks had shown enrichment at 2-weeks as well, with some of their activity changes being more predictable at 3-weeks. For example, NRF2-mediated oxidative stress response was statistically the most significant pathway at 2-weeks and its enrichment remained significant at 3-weeks but the pathway activity was predicted from being unchanged (z-score = 0) at 2-weeks to being increased at 3-weeks (positive z-score). Similarly, the aryl hydrocarbon receptor (AhR) signaling was predicted from being unchanged at 2-weeks to being decreased at 3-weeks (negative z-score). The AhR is a transcription factor of the basic helix-loop-helix/PER-ARNT-SIM family. It can be activated by both environmental factors (e.g., polycyclic aromatic hydrocarbons or dioxins) and endogenous ligands (e.g., tryptophan derivatives, arachidonic acid metabolites, equilenin, heme metabolites, and indigoids). Ligand-bound or activated AhR moves to the nucleus where it forms heterodimers with aryl hydrocarbon receptor nuclear translocator (ARNT). The AhR/ARNT heterodimer binds to xenobiotic responsive elements (XREs) or dioxin responsive elements (DREs) in the enhancer sequences of genes encoding phase I and II xenobiotic metabolizing enzymes, such as cytochrome P450 monooxygenases (CYP1A1, CYP1A2, and CYP1B1) and glutathione-S-transferases (GSTs), NADPH/quinone oxidoreductase (NQO1), and aldehyde dehydrogenase 3, and activates these genes (Dietrich, 2016). Decreased AhR signaling in Hom-CKO hearts may conceivably diminish the ability to detoxify exogenous and endogenous toxic factors, which may contribute to cardiac dysfunction and cardiomyocyte death. Beyond activating the canonical xenobiotic metabolism signaling, AhR can also trigger the antioxidant response through transactivating the NRF2 gene and collaborating with NRF2 to activate NRF2 target genes (Dietrich, 2016). The NRF2-mediated oxidative stress response is a well-known master pathway that enables the cell to deal with harmful stress including oxidative stress. The enrichment of DEGs in these pathways related to oxidative stress responses strongly suggests that cardiac CSN8 deficiency increases oxidative stress and something alike which in turn trigger cellular defense against these toxic stresses. This is supported by a prior transcription profiling study on *Drosophila* deficient of a CSN subunit which also indicates that CSN regulates the redox signaling pathways (Oron et al., 2007). This is further corroborated by our prior findings that increased myocardial protein carbonyls and massive cardiomyocyte necrosis are observed in mice with cardiac deficiency of CSN8 (Su et al., 2011a,b, 2013). Enrichment of DEGs in the sperm motility pathway is consistent with dysregulated microtubule functioning that is evidenced by perturbed vesicle trafficking (Table 3) and impaired autophagosome maturation in Hom-CKO mouse hearts (Su et al., 2011a). Comparisons between Hom-CKO and CTL and between Het-CKO and CTL are shown in Supplementary Figures 5, 6, respectively.

We also performed IPA analysis on DEG data generated from comparison of gene expression between 3- and 2-weeks of age within each genotype (i.e., data summarized in Figure 1C). The analysis reveals that there were 4 and 12 pathways with statistically significant enrichment of the DEGs in the CTL and Het-CKO mice, respectively but the overall activity of these pathways was either unchanged or not predictable (Supplementary Figure 7). By sharp contrast, this analysis has identified in the Hom-CKO group many more pathways with significant enrichment of DEGs, with 10 of them showing increased activity and one showing decreased activity (Figure 3C). Notably, 7 of the 10 activity-increased pathways (e.g., leukocyte extravasation signaling, complement system, IL-1 signaling, Gαi signaling, MIF regulation of innate immunity, STAT3 pathway, and eicosanoid signaling) and the only activity-decreased pathway (i.e., LPS/IL-1 mediated inhibition of RXR function) are known to fall in the upstream signaling, the execution, or the downstream signaling of necrosis. The remaining 3 activity-increased pathways (e.g., AhR signaling, NRF2-mediated oxidative stress response, and LXR/RXR activation) are known to deal with xenobiotic metabolism and oxidative stress. Hence, these longitudinal changes in pathway genes correlate extremely well to the observation that massive cardiomyocyte necrosis occurs in Hom-CKO hearts but it was not discernible until 3 weeks of age (Su et al., 2011a,b).

### Network analysis of hand-curated pathway genes

The co-expression network of hand-curated pathway genes is shown in Supplementary Figure 8. To generate this network, we used array data for hand-curated pathway genes. The co-expression network helped us to identify interactions among genes of same or different pathways. Hence, each module/cluster in the co-expression network, designated with different colors, represents genes from multiple pathways that showed similar expression patterns (Supplementary Figure 8). There were 13 different modules identified in our co-expression network with blue module and turquoise module as the two densest modules containing the majority of differentially expressed pathway genes (Table 4). The differentially expressed pathway genes usually had more interacting neighbors, indicating their pivotal roles in influencing other genes. In total, network analyses identified co-expression of 28 DEGs (Triangle) along with some genes that are missing in statistical analysis (Circle). For example, *Pam, Sqstm1, Tpm2* in the blue module, and *Traf3, Myo9b, C1qtnf4, Bcl2, Bcl10, Myl9, Dnalc4, Kif5b, Tnfsf5ip1, Eml1* in the turquoise module could be genes affected by CSN8-CKO. These genes, although not detected as significant DEGs from our gene array data, are in the same module of most of the differentially expressed pathway genes, and have larger numbers of interacting neighbors, including those differentially expressed pathway genes (Supplementary Figure 8). Table 4 shows the total number of genes in each identified module from network analysis, and the corresponding number of genes of each pathway enriched in each module. For example, in the blue module; the genes with the most interacting partners includes *Syn2* (trafficking), *ssb4* (SOCS-box), *Mical3* and *Tppp* (microtubules) with 53, 49, 47, and 46 interactions, respectively. Similarly *Tnfaip8* (cell death), *Ttll1* (microtubules), *Hist1h1c* and *Hist2h2bb* (chromatin remodeling) showed 60, 52, and 51 interactions in the turquoise module, respectively. These genes do not necessarily interact with DEGs only but also with non-DEGs genes (Supplementary Figure 8). In summary, the co-expression network map demonstrates that CSN8-CKO-induced effects on CRLs substrates of UPS, autophagy, cell death, vesicle trafficking, chromatin remodeling and microtubule-related pathways are all interlinked. CSN8-induced transcriptional effects on genes of one pathway can have direct or indirect effects on other pathways.

**Table 4:** Distribution of various pathway genes in each module of co-expression network.

**No**.	**Module**	**Total genes**	**DEGs**	**Cell death**	**Microtubules**	**Fetal genes**	**Autophagy**	**Vesicle trafficking**	**SOCS box**	**F-box**	**Chromatin remodeling**
1	Turquoise	50	10	15	18	1	4	4	2	1	5
2	Blue	46	15	15	13	3	5	3	2	2	3
3	Brown	23	0	3	15	0	1	2	1	0	1
4	Yellow	20	1	10	9	0	0	0	0	1	0
5	Green	12	0	3	6	0	2	1	0	0	0
6	Red	11	0	5	4	0	1	1	0	0	0
7	Magenta	9	1	2	5	0	0	1	0	0	1
8	Greenyellow	8	0	3	5	0	0	0	0	0	0
9	Black	8	0	4	3	0	0	1	0	0	0
10	Pink	7	0	2	5	0	0	0	0	0	0
11	Gray	6	0	2	3	0	1	0	0	0	0
12	Purple	6	0	3	3	0	0	0	0	0	0
13	Tan	5	1	2	1	0	1	0	0	1	0

### DEGs induced by CSN8 deficiency enrich in the chromatin remodeling pathway

**Figure 4:**
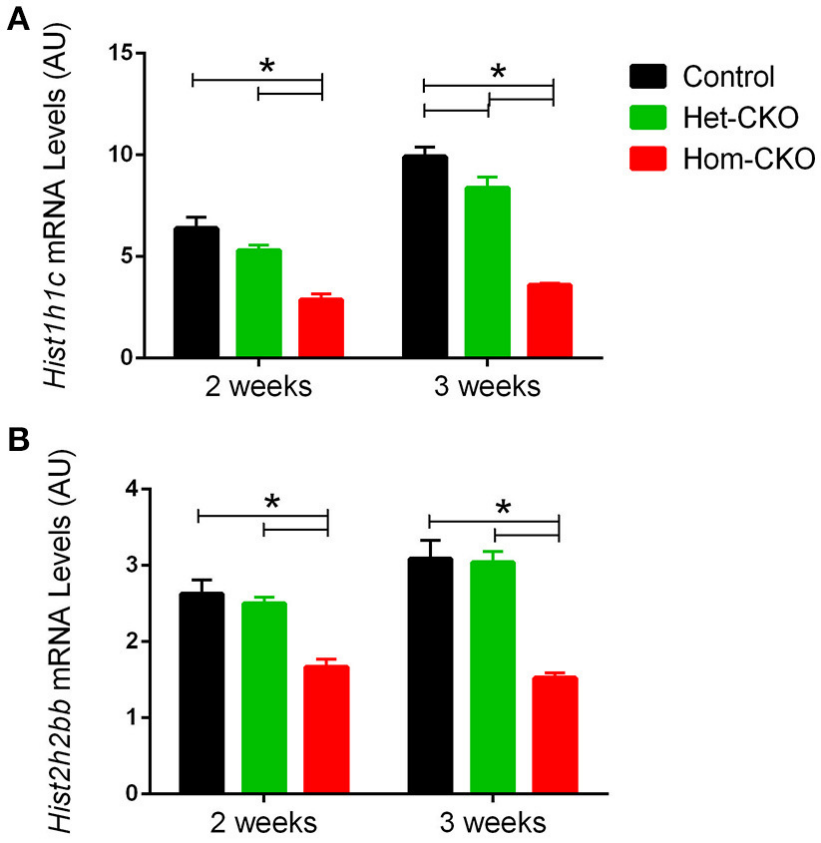
Microarray-derived mRNA expression levels of the *Hist1h1c* **(A)** and *Hist2h2bb* genes **(B)** in CTL, Het-CKO, and Hom-CKO littermate mice at 2- and 3-weeks-of-age. Mean ± SEM, *n* = 3 mice/group; ^*^p < 0.05, one way ANOVA followed by Tukey's modified Student's *t*-test. *Hist1h1c*, Histone1, H1c; *Hist2h2bb*, Histone 2, H2bb.

The eukaryotic DNA is packaged around histone proteins in a structure called chromatin. In order for transcriptional regulators to get access to genomic DNA, chromatin structure needs to be modulated via a process known as chromatin remodeling (Hughes and Rando, 2014). As shown in Table 3, chromatin remodeling is one of the processes with significant enrichment of DEGs in Hom-CKO hearts. Histone proteins act as crucial regulators of transcription through chromatin remodeling. Hence, we paid particular attention to expression changes of histone gens. In CSN8-CKO myocardium, not only was the expression of two of the histone genes *Hist1h1c* and *Hist2h2bb*, significantly affected (Figure 4), but these two DEGs were also shown to have the most number of interactions with other pathway genes (Supplementary Figure 8), indicative of a significant impact of CSN8 deficiency on transcriptional regulation.

### CSN8 deficiency decreased the transcript levels of CRL substrate receptors

**Figure 5:**
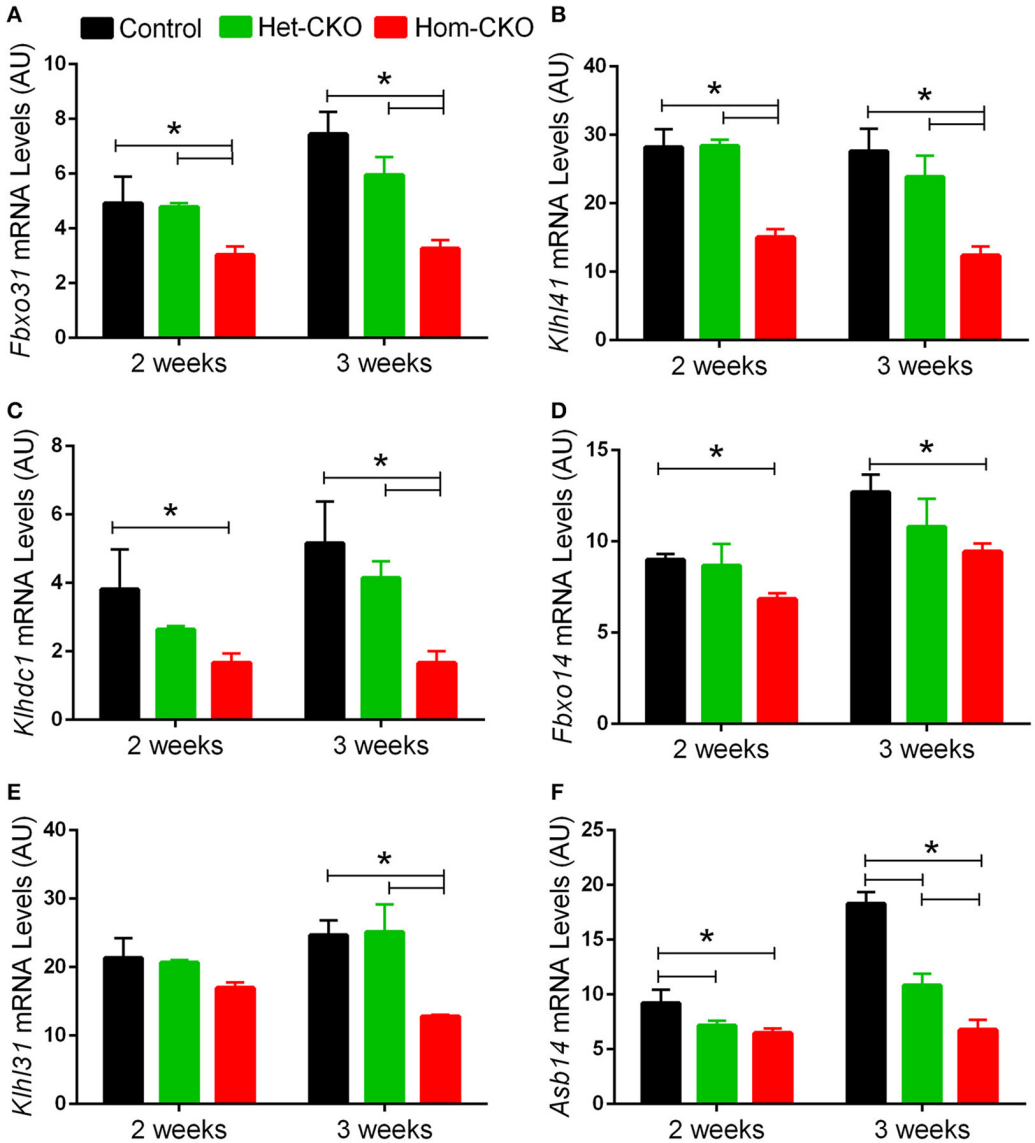
Cardiac CSN8 deficiency causes significant changes in the mRNA levels of myocardial CRL substrate receptors in mice. Shown are microarray-derived mRNA expression levels of the indicated CRL substrate receptors in CTL, Het-CKO, and Hom-CKO littermate mice at 2- and 3-weeks-of-age. **(A)** *Fbxo31*, F-box only protein 31; **(B)** *Klhl41*, Kelch like 41; **(C)** *Klhdc1*, Kelch domain containing 1; **(D)** *Fbxo14*, F-box only protein 14; **(E)** *Klhl31*, Kelch like 31; **(F)** *Asb14*, ankyrin repeat and SOCS box-containing protein 14. Mean ± SEM, *n* = 3 mice/group; ^*^p < 0.05, one way ANOVA followed by Tukey's modified Student's *t*-test

**Figure 6:**
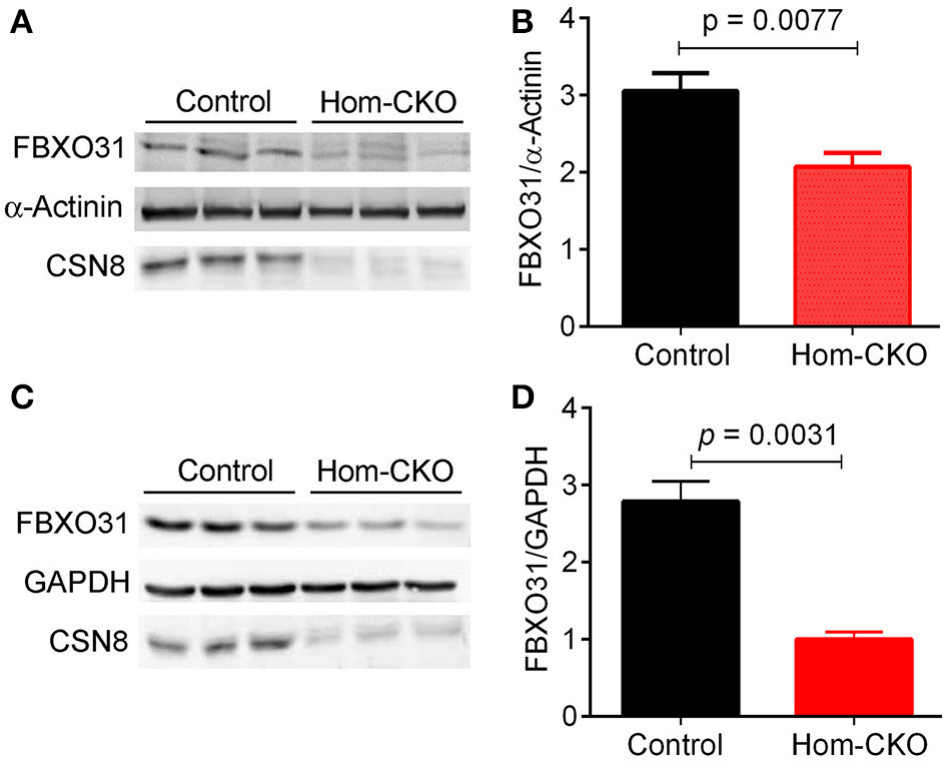
Western blot analyses for myocardial FBXO31 in control and homozygous Csn8-CKO (Hom-CKO) mice at 2 weeks **(A,B)** and 3 weeks **(C,D)** of age. α-Actinin and GAPDH were probed as loading controls. Mean ± SEM, *n* = 3 mice/group, Student's *t*-test.

**Figure 7:**
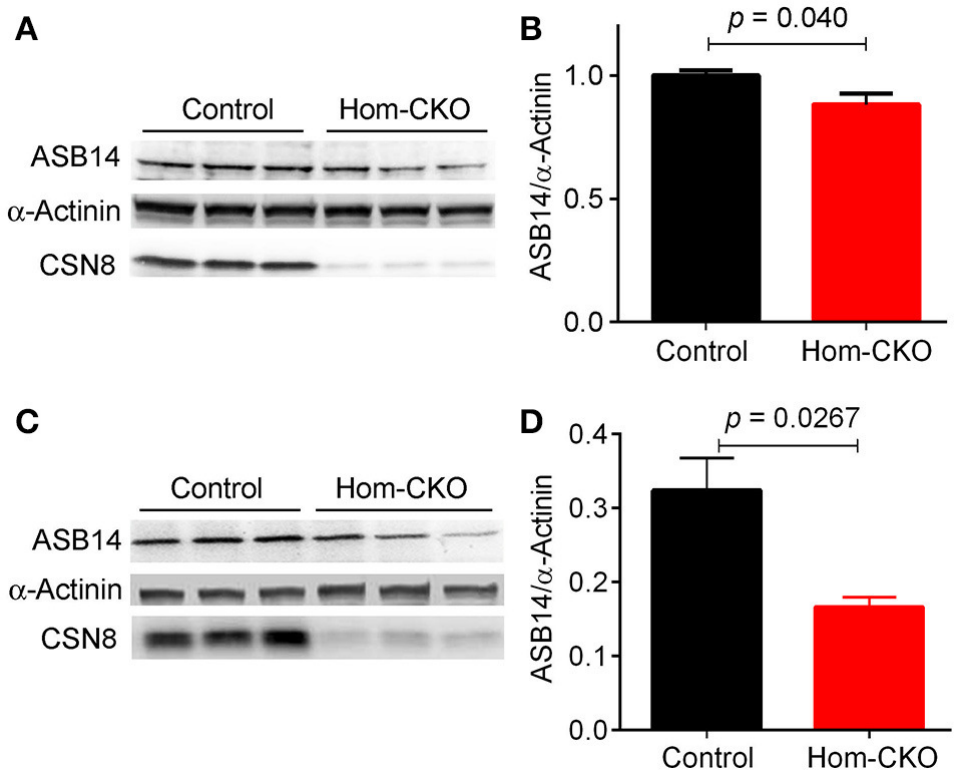
Western blot analyses for myocardial ASB14 in control and homozygous Csn8-CKO (Hom-CKO) mice at 2 weeks **(A,B)** and 3 weeks **(C,D)** of age. α-Actinin was probed as loading control. Mean ± SEM, *n* = 3 mice/group, Student's *t*-test.

A major purported role for CSN-mediated cullin deneddylation to regulate CRLs is to prevent autoubiquitination and subsequent destruction of SR proteins of the CRL complexes, thereby preserving SRs of CRLs (Wee et al., 2005; Wang and Martin, 2015). Indeed, the decreased protein levels of SRs were observed in cells and tissues deficient of CSN subunits (Wee et al., 2005; Su et al., 2011b). We have previously observed in Csn8-deficient heart and liver tissues that the deneddylase activity of the CSN is compromised, which results in significantly decreased protein levels of various SR molecules (Lei et al., 2011, 2013; Su et al., 2011b, 2013). Here, we analyzed the mRNA levels of several families of SR genes in response to CSN8-CKO (Table 3, Figure 5). The expression of SR genes *Fbxo31, Klhl41 and Klhdc1*, was significantly lower in Hom-CKO heart as compared to CTL and Het-CKO counterparts at both 2- and 3-weeks-of-age (Figures 5A–C). Similarly, we found that at 2- and 3-weeks-of-age, the expression of *Fbxo14* SR gene was significantly lower in Hom-CKO heart when compared to CTL but not to heterozygous KO heart (Figure 5D). The expression of *Klhl31* gene was significantly lower in CSN8-CKO hearts when compared to CTL and Het-CKO littermates at 3-weeks-of-age only (Figure 5E). Finally, the expression of a SOCS box containing gene *Asb-14* was significantly lower in Hom-CKO hearts at 2- and 3-weeks-of-age. We observed that loss of one CSN8 allele also led to decreased expression of *Asb-14* gene (Figure 5F). We then decided to look at the protein expression of two SR genes *Fbxo31* and *Asb-14* in CSN8-CKO heart tissue. FBXO31 protein expression following CSN8-CKO was significantly decreased in Hom-CKO hearts compared to the control group at 2-weeks (Figures 6A,B) and 3-weeks-of-age (Figures 6C,D). Similarly, Asb-14 protein levels in CSN8-CKO mouse hearts were significantly decreased at 2-weeks (Figures 7A,B) and 3-weeks-of-age (Figures 7C,D), when compared with control mice.

### DEGs induced by CSN8 deficiency enrich in microtubule-related genes

Since our analyses revealed significant enrichment of DEGs in the microtubule-related pathways in response to CSN8-CKO (Supplementary Table 9), we decided to also examine the protein level of differentially expressed microtubule-related genes. Microtubules in the cytoplasm of the cell are involved in vesicular trafficking between endoplasmic reticulum (ER) and Golgi apparatus. Microtubules are also required for chromosome segregation during cell division (Vaughan, 2005). A few studies have reported a link between the CSN-UPS pathway and the microtubule network. For example, failure to degrade MEI-1 subunit of the MEI-1/MEI-2 complex that is localized to the spindle and chromosomes during meiosis results in defects in spindle positioning and elongation (Pintard et al., 2003). Another study showed that the downregulation of the CSN led to the reduction of microtubule end-binding protein 1 (EB1) which is a regulator of microtubule dynamics (Peth et al., 2007). CSN3 or CSN5 knockdown caused disrupted meiotic spindle and misarranged chromosomes that result in failure to complete meiosis (Kim et al., 2011). Tubulin tyrosine ligase like-1 (TTLL1) belongs to the family of TTLL enzymes that catalyze the post-translational modifications of tubulin in order to modulate microtubule dynamics and function (Pathak et al., 2011). TTLL1-deficient mice were reported to develop lesions in the respiratory and male reproductive tracts (Vogel et al., 2010). Expression of *TTLL1* gene has been found to be significantly decreased in the case of pathological hypertrophy (PAH) (Song et al., 2012). Since previous findings from our lab have reported that CSN8-CKO mice also developed cardiac hypertrophy, we sought to determine the effect of CSN8 deficiency on *TTLL1* gene expression. The microarray data revealed that expression of the *TTLL1* gene in response to CSN8-CKO was significantly down at both 2 and 3-weeks-of-age (Supplementary Figure 9A). In fact, *TTLL1* was among top genes that were downregulated in CSN8-CKO heart tissue (Table 1). The expression of TTLL1 protein in CSN8-CKO heart tissues did not show discernible change at 2-weeks-of-age (Supplementary Figures 9B,C) but it was markedly decreased at 3-weeks-of-age (Supplementary Figures 9D,E), compared with the respective CTL. It will be interesting to determine the role of TTLL1 down-regulation in impaired autophagosome maturation observed in CSN8 deficient cardiomyocytes.

## Discussion

### Is CSN8 a transcriptional regulator in murine hearts?

Cullin-RING ligases (CRLs) are the largest subfamily of ubiquitin ligases, responsible for approximately 20% of the ubiquitin-dependent protein degradation in the cell (Petroski and Deshaies, 2005; Soucy et al., 2009). Although the CSN5-mediated deneddylation of CRLs is the most important activity of the CSN complex, and the loss of this activity results in lethality in most of the cases, not all CRLs are sensitive to deneddylation (Chamovitz, 2009). For example, SCF^β−*TrcP*^-mediated IκB degradation, SCF^Skp2−^mediated downregulation of p27 and SCF^Slimb^-mediated degradation of the IκB homolog Cactus and the clock protein TIM are unaffected after knockdown of different CSN subunits (Harari-Steinberg et al., 2007; Menon et al., 2007; Schweitzer et al., 2007; Panattoni et al., 2008; Knowles et al., 2009). These examples emphasize the importance of CSN's non-deneddylation activities which include deubiquitination, regulation of protein subcellular localization, associated kinase activity and transcription regulation (Chamovitz, 2009). Since the original description of the CSN was given as a repressor of *Arabidopsis* genes (Wei and Deng, 1992), the role of the CSN in regulating the activity of transcription factors has been shown in many species (Wei et al., 2008). The mammalian CSN5 was originally identified as a transcriptional co-activator for c-Jun of AP-1 regulatory protein (Claret et al., 1996). In T-lymphocytes, deficiency of CSN8 leads to elevation of cell cycle regulating genes cyclin E1, cyclin D2 and E2F1 relative to wild-type mice (Menon et al., 2007). Our group has also shown previously that down-regulation of CSN8 in HEK293 cells resulted in reduced transcript levels of cyclin kinase inhibitor p21^cip^ and p27^kip^ (Su et al., 2009). In *Drosophila*, mutation of CSN subunits 4 and 5 leads to dysregulation of the transcriptome during early larval development, which indicates that a primary effect of CSN perturbation is a change in gene expression profiles. Furthermore, these results indicate that in the absence of CSN4 and CSN5, the transcription of numerous early developmental genes is activated. On the other hand, the transcription of a large set of genes was found to be decreased in the absence of the CSN, which also illustrates the CSN as a transcriptional activator (Oron et al., 2007). Our previous studies have identified striking phenotypes in mice with cardiac deficiency of CSN8 (Su et al., 2011a,b); however, the mechanism responsible for CSN8 deficiency to cause the cardiac pathology, such as massive cardiomyocyte necrosis, remains obscure. To explore the potential involvement of altered gene expression, here we performed transcriptome analysis at postnatal 2 and 3 weeks-of-age. Gene microarray data revealed that there were more DEGs in response to CSN8 ablation at 3 weeks than at 2 weeks (Figure 1). In response to CSN8 deficiency, expression of a large number of genes was found to be upregulated, with *Prkag3, Cilp, Grm1, Cdkl1*, and *Parp6* being the top 5 upregulated genes at 2-weeks-of-age (Table 1). On the other hand, we also found a number of genes that showed decreased expression at 2 weeks in response to CSN8-CKO. Among the top down-regulated genes were *Syp, Phkg1, Aqp4, Ttll1*, and *Pfkfb1* (Table 2). These results are in agreement with a previous report that the CSN can act as both transcriptional repressor and transcriptional activator (Oron et al., 2007). However, our results are in contrast to earlier description of the CSN as transcriptional repressor only (Wei and Deng, 1992). The potential regulatory role of CSN8 was further evaluated by examining the expression of hand-curated genes of eight different pathways that we expected to be affected by CSN activity (Table 3). The eight different sets of genes include SRs of CRLs and chromatin remodeling genes (discussed in separate sections), autophagy, microtubules, vesicle trafficking, endocytosis, ATP synthases, and those of cell death pathways. All but the cell death pathways were enriched with DEGs. DEGs that show similar expression pattern were clustered together (Figure 2). Moreover, interaction among hand-curated genes is shown in co-expression network map (Supplementary Figure 8). This network shows changes in interaction of a single gene following CSN8-CKO. Synapsin II (vesicle trafficking) and TTLL1 (microtubules) were among top five DEGs in response to CSN8 ablation in mouse hearts (Tables 1, 2). Further investigation reveals that TTLL1 protein levels were significantly decreased in Hom-CKO mouse hearts at 3 weeks of age (Supplementary Figure 9). Together these data are consistent with the notion that CSN8/CSN likely regulates the gene expression of multiple pathways in murine hearts.

### Is CSN8 a chromatin-based transcriptional regulator?

Several studies have suggested the involvement of the CSN in transcriptional regulation and that the CSN regulates transcription either by regulating the protein stability of transcription factors or by directly acting on chromatin along with other components of the UPS (Chamovitz, 2009). Various transcription factors have been identified as interactors of CSN subunits (Kato and Yoneda-Kato, 2009). *Drosophila* CSN4 was reported to interact with Rbf-targeted promotor region of *Pol*α and *PCNA* genes after targeting Rbf protein (Ullah et al., 2007). CSN1 and CSN8 were reported to bind to Ccnd2, Cdk4 and Cdkn1a promotors (Menon et al., 2007). CSN5 was originally identified as a coactivator of Ap-1 transcription factor binding sites (Claret et al., 1996). CSN2 was originally identified as a corepressor of steroid hormone signaling (Dressel et al., 1999). To our knowledge, a direct effect of CSN subunits on chromatin has not been reported. Here we have shown for the first time that CSN8 deficiency leads to decreased expression of two of the histone genes; *Hist1h1c* and *Hist2h2bb* (Figure 4). These two, along with other histone proteins, are responsible for keeping the fidelity and regulating access to genomic DNA in order to modulate gene transcription (Hughes and Rando, 2014). Hence, it is tempting to hypothesize that CSN8 may regulate gene transcription through chromatin remodeling. To test this hypothesis we will need to determine whether the decreased mRNA levels are accompanied by altered protein levels and examine the interaction of CSN8 with the regulatory elements of these histone genes.

### CSN8 may regulate the transcription of CRL substrate receptors

The specificity of CRLs toward a target protein depends on the SR module binding to the amino terminus of cullin scaffold (Duda et al., 2011). It has been estimated that the human genome encodes about 200 SR's, each of which may bind with one of the eight cullins to form a specific CRL (Wang and Martin, 2015). Members of CRLs and more specifically, their SRs, play important roles in numerous physiological functions and dysfunction and thereby are involved in a wide range of diseases (Bulatov and Ciulli, 2015). Several SRs, for example, members of the F-box protein family, such as Skp2, β-TrCP, Fbw7 and Fbxl3, were shown to play significant roles in cancer and other diseases (Wang et al., 2014). Overexpression of β-TrCP has been reported in different types of cancer and during the inflammatory process (Frescas and Pagano, 2008; Skaar et al., 2013). Another SR VHL recognizes hypoxia inducible factor (HIFα) for degradation by CRL2^VHL^ and acts as a tumor suppressor protein. Mutation of VHL is reported in von Hippel–Lindau (VHL) hereditary cancer syndrome (Zhang and Yang, 2012). Similarly, Fem1b of CRL2^FEMB1^ promotes ubiquitination and suppressed transcriptional activity of Gli1 oncoprotein in humans (Gilder et al., 2013). It is for these reasons that the stability of SRs of CRLs needs to be regulated efficiently. The detachment of NEDD8 from activated CRLs is believed to prevent autoubiquitination and subsequent destruction of the SR when the substrate is running low. This is also necessary for the disassembly and remodeling of CRL complexes (Duda et al., 2011; Pierce et al., 2013; Wang and Martin, 2015). Although cells and tissues deficient of CSN subunits showed decreased protein levels of SRs (Wee et al., 2005; Cope and Deshaies, 2006; Su et al., 2011b) it has not yet been confirmed if the known reduction of SR protein levels can be caused by decreased expression at the transcription level, in addition to the proposed self-destruction of the SR proteins. Our results have clearly shown that CSN8 deficiency leads to decreases in both the mRNA levels (Figure 5, Supplementary Tables 3, 4) and the protein levels (Figures 6, 7) of a number of SRs of the F-box and SOCS-box families. To our best knowledge, this is the first study that has reported the transcript levels of SR genes are markedly down-regulated by ablation of a CSN subunit. In general, a decrease in the protein level of a gene is expected to upregulate its transcription via a negative feedback mechanism. As exemplified by FBXO31 and ASB14, we observed concomitant decreases in both the mRNA levels and the protein levels for SRs in Hom-CKO mouse hearts at both 2 and 3 weeks of age, suggesting that the decreased transcription contributes to the decreased protein levels. Autoubiquitination and resultant self-destruction of the SR in the CRL has been the predominant theory for explaining the decreased SR proteins observed in CSN deficient cells and, based on this theory, the mechanism by which the CSN regulates a CRL has been commonly believed to preserve its own SR upon the completion of ubiquitination of its substrates by the CRL. Our findings reported here obviously challenge this theory. Hence, we propose that CSN8/CSN regulates the SR protein levels and thereby the activity of CRLs through both transcriptional and post-translational mechanisms.

## Author contributions

XW, Conception and experimental design of the study, data analysis and interpretation, and manuscript preparation; AA, Data collection, analysis, interpretation, and manuscript preparation; KE, Experimental design, data collection and analyses, and preparation of the manuscript; TB, PX data collection and analyses; EZ, bioinformatics and statistical analyses of the microarray data, preparation of the related sections of the manuscript.

### Conflict of interest statement

The authors declare that the research was conducted in the absence of any commercial or financial relationships that could be construed as a potential conflict of interest.
