# Supplementary material for: Murine Myocardial Transcriptome Analysis Reveals a Critical Role of COPS8 in the Gene Expression of Cullin-RING Ligase Substrate Receptors and Redox and Vesicle Trafficking Pathways
Source: Front Physiol. 2017 Aug 17;8:594. doi: 10.3389/fphys.2017.00594 (PMC5562692; doi:10.3389/fphys.2017.00594)
Supplement: Supplementary file 1 [file DataSheet1.PDF]

Supplementary Material

Murine Myocardial Transcriptome Analysis Reveals a Critical Role of COPS8 in the Gene Expression of Cullin-RING Ligase Substrate Receptors and Redox and Vesicle Trafficking Pathways

Ammara Abdullah[1], Kathleen M. Eyster[1], Travis Bjordahl[1#], Peng Xiao[1], Erliang Zeng[1,2], Xuejun Wang[1]*

[1]Division of Basic Biomedical Sciences, Sanford School of Medicine of the University of South Dakota, Vermillion, SD, USA.

[2]Department of computer sciences and Department of Biology, University of South Dakota, Vermillion, SD, USA.

*Correspondence:

Xuejun Wang

xujeun.wang@usd.edu

Supplementary Figures 1~9

Supplementary Tables 1 ~ 10

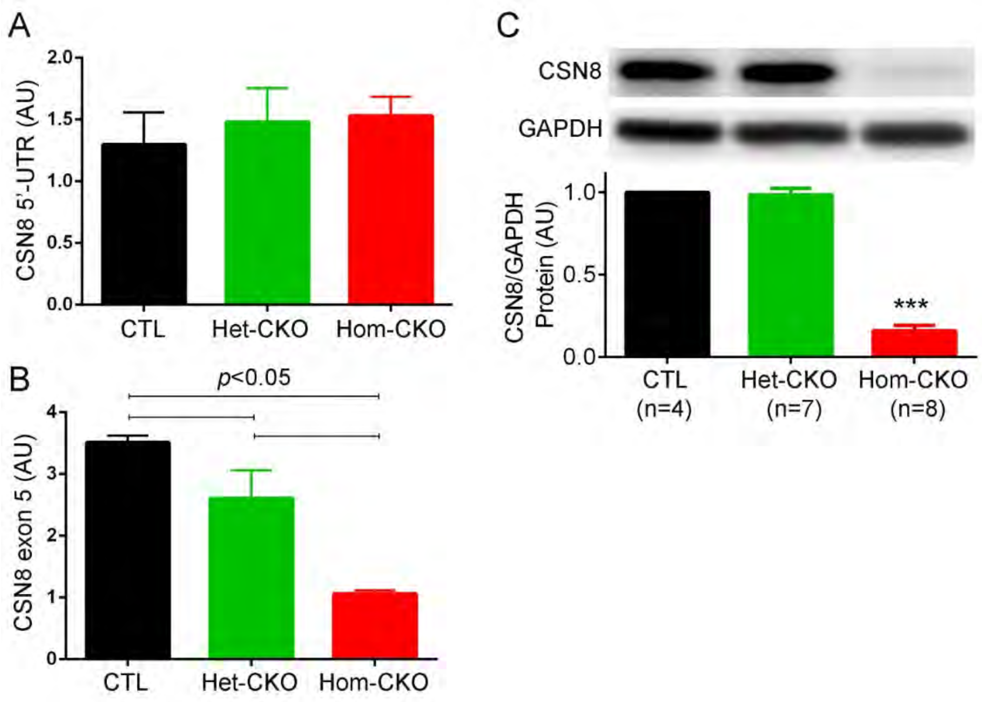

Supplementary Figure 1. Changes in myocardial CSN8 mRNA and protein levels in mice cardiomyocyte-restricted Cops8 heterozygous (Het-CKO) and homozygous (Hom-CKO) knockout compared with littermate controls (CTL). (**A**, **B**) Myocardial CSN8 transcript levels at 2 weeks of age detected with RT^2-PCR using primers/probes specific for the 5'UTR region (**A**) or the exon 5 region (**B**) of the *Cops8* gene. (**C**) Western blot analyses for myocardial CSN8 protein levels at 3 weeks of age. Shown are representative images (upper portion) and pooled densitometry data (lower portion). ***$p<0.001$ vs. both CTL and Het-CKO.

A) Fetal Genes

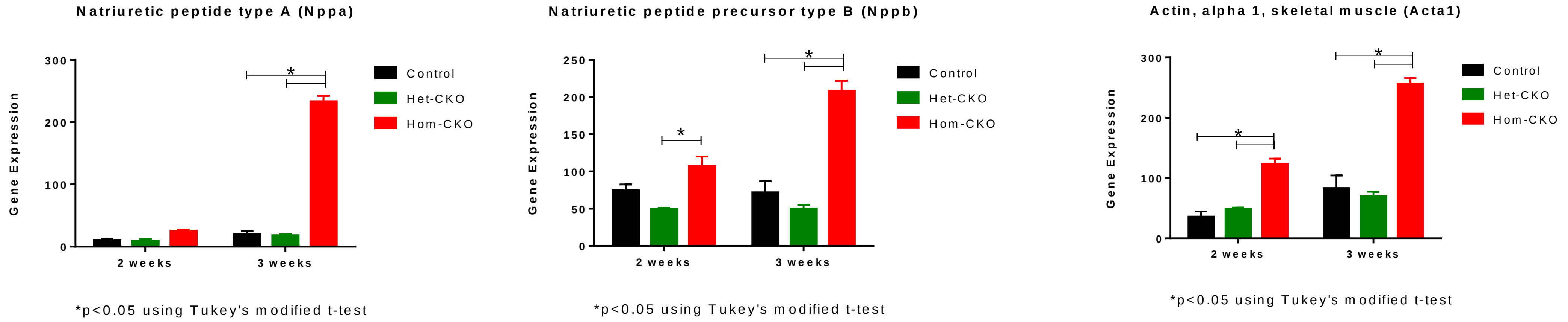

B) Post-natal Genes

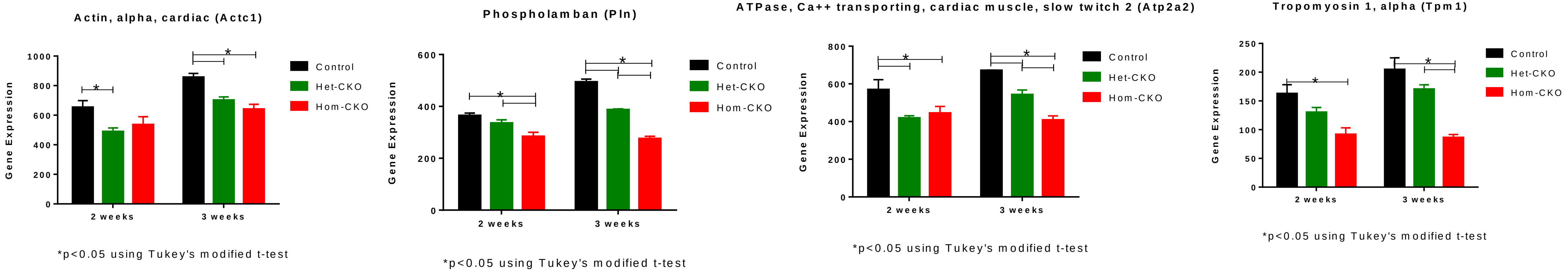

Supplementary Figure 2. Gene expression profiling reveals marked changes in the fetal gene program that are consistent with cardiac pathology in CSN8-CKO mice. Differential gene expression of (Panel A) fetal genes and (Panel B) postnatal genes CTL, Het-CKO, and Hom-CKO littermate mice at 2- and 3-weeks-of-age (n=3). The data are presented as means ± SEM. Statistical significance among the groups was determined by ANOVA followed by Tukey's modified Student's t-test as the post hoc test. All findings were considered statistically significant with *$p<0.05$.

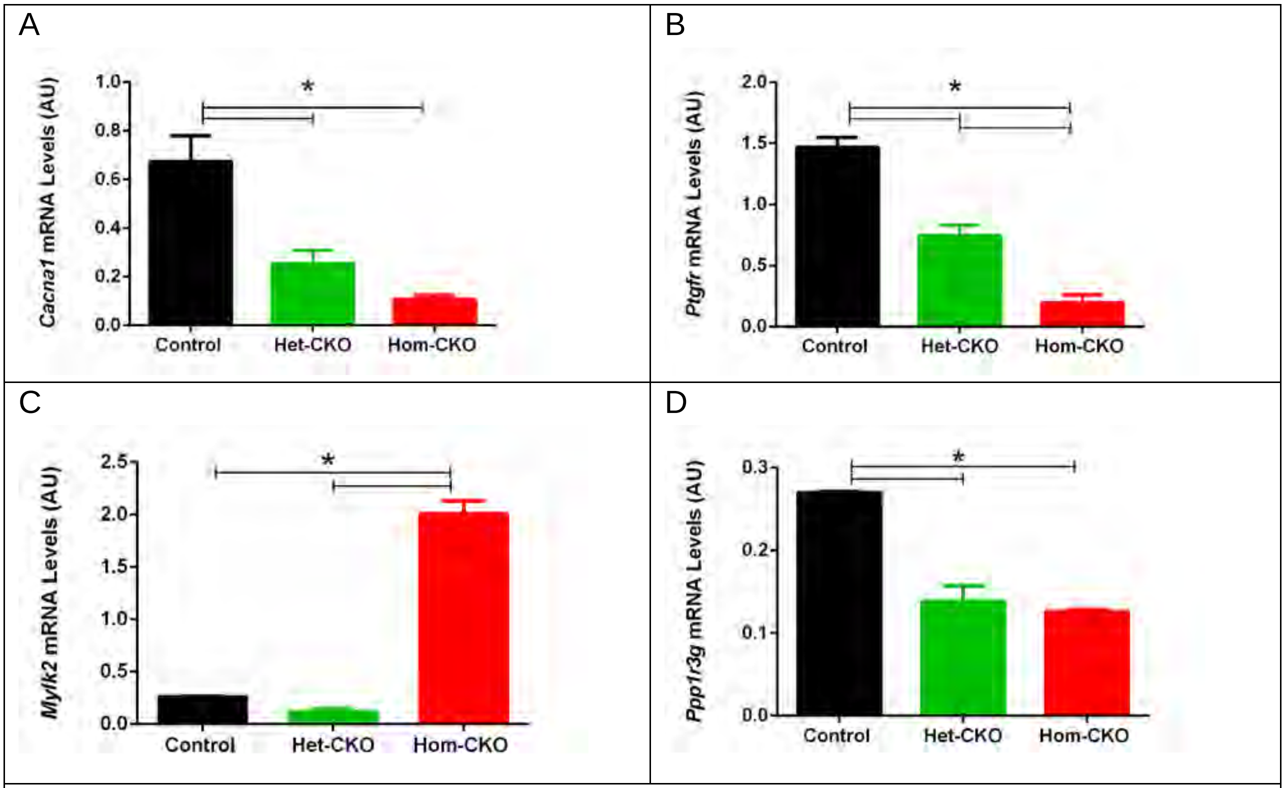

Supplementary Figure-3. Gene microarray data of differentially-expressed genes that are common among three comparisons: Control vs. Hom-CKO, Control vs. Het-CKO and Hom-CKO vs. Het-CKO at 3-weeks-of-age. **(A**) Dihydropyridine sensitive skeletal muscle calcium channel (*Cacna1*) gene. (**B**) Prostaglandin F receptor (*Ptgfr1*) gene. (**C**) Myosin light polypeptide kinase 2 (*Mylk2*) gene. (**D**) Protein phosphatase 1, regulatory (inhibitor) subunit 3 (*Ppp1r3g*) gene. Mean±SEM, n=3 mice/group; *p<0.05, one way ANOVA followed by Tukey's modified Student's *t*-test.

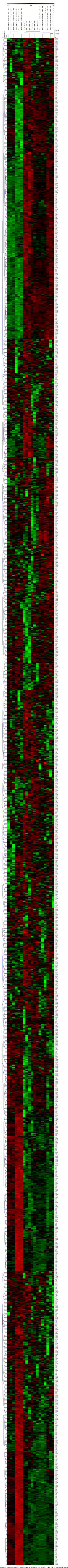

Supplementary figure 4. Heatmap/hierarchical clustering of all DEGs in microarray data. All replicates of each group i.e. Control, Het-CKO and Hom–CKO. have been shown. Similarity between clusters is shown with the help of dendograms. Vertical dendograms represent expression value from microarray data. Green and red indicate low and high levels of transcript abundances respectively.

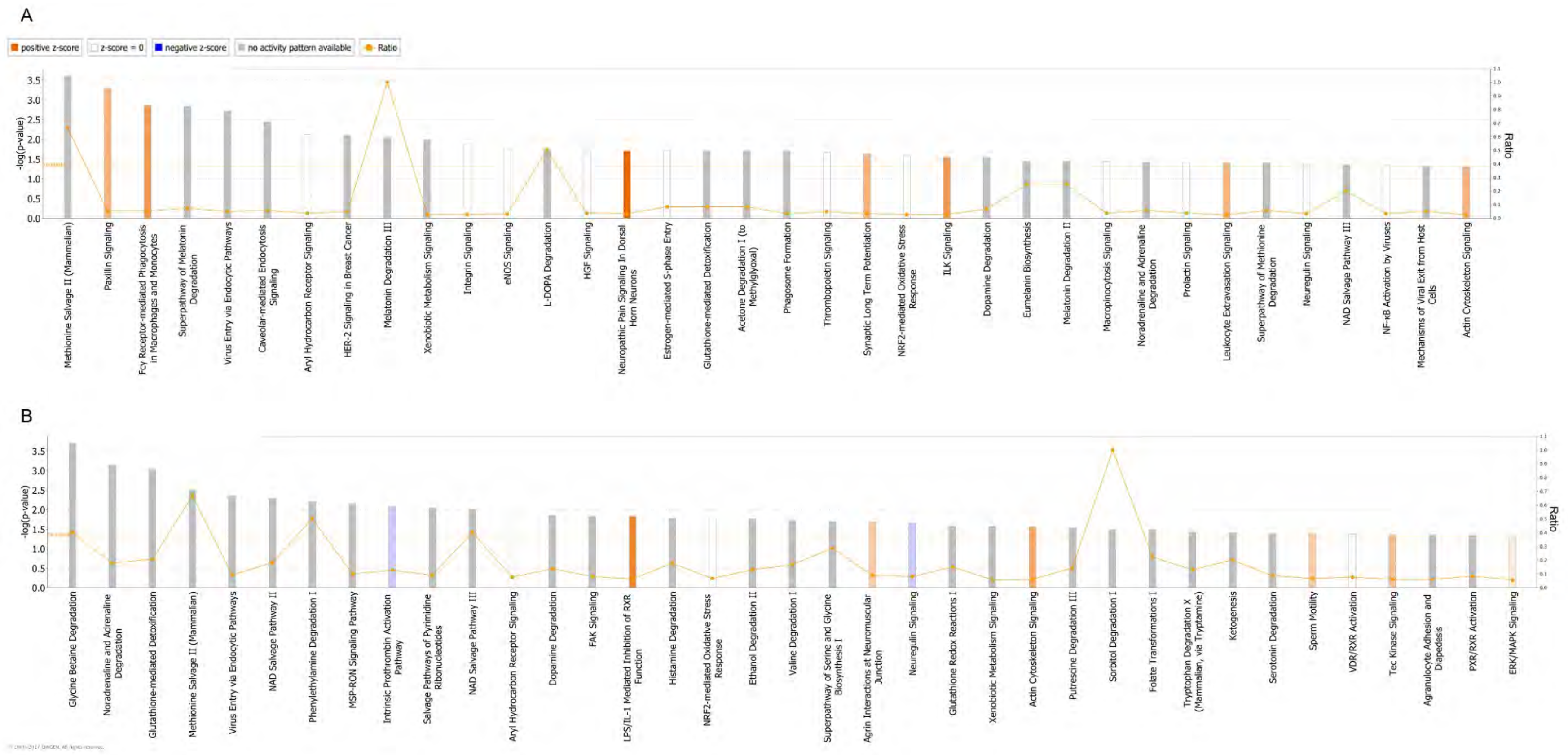

Supplementary Figure 5. Significantly enriched (*p*-value<0.05; Fisher's exact test) Ingenuity canonical pathways. The x-axis represents significant canonical pathways while the left Y-axis displays the -log of *p*-value which is calculated by Fisher's exact test right-tailed. Thus taller bars equate to increased significance. The threshold is set at -log of 0.05 (*p* value) so everything above or at that threshold denotes significant change. The overall activation/inhibition states of canonical pathways are predicted based on a Z-score algorithm. The canonical pathway bar charts are colored to indicate their activation z-scores. Orange bars predict an overall increase in the activity of the pathway while blue bars indicate a prediction of an overall decrease in activity. White bars are those with a z-score which is zero or very close to 0, predicting unchanged overall activity of the pathway. Gray bars indicate pathways that are currently ineligible for a prediction. The orange points connected by a thin line represent the Ratio (right Y-axis). The ratio is calculated as the number of genes in a given pathway that meet cutoff criteria, divided by the total number of genes that make up that pathway and that are in the reference gene set. Panel A: Hom-CKO vs. Control heart samples at 2-weeks-of-age. Panel B: Hom-CKO vs. Control heart samples at 3-weeks-of-age.

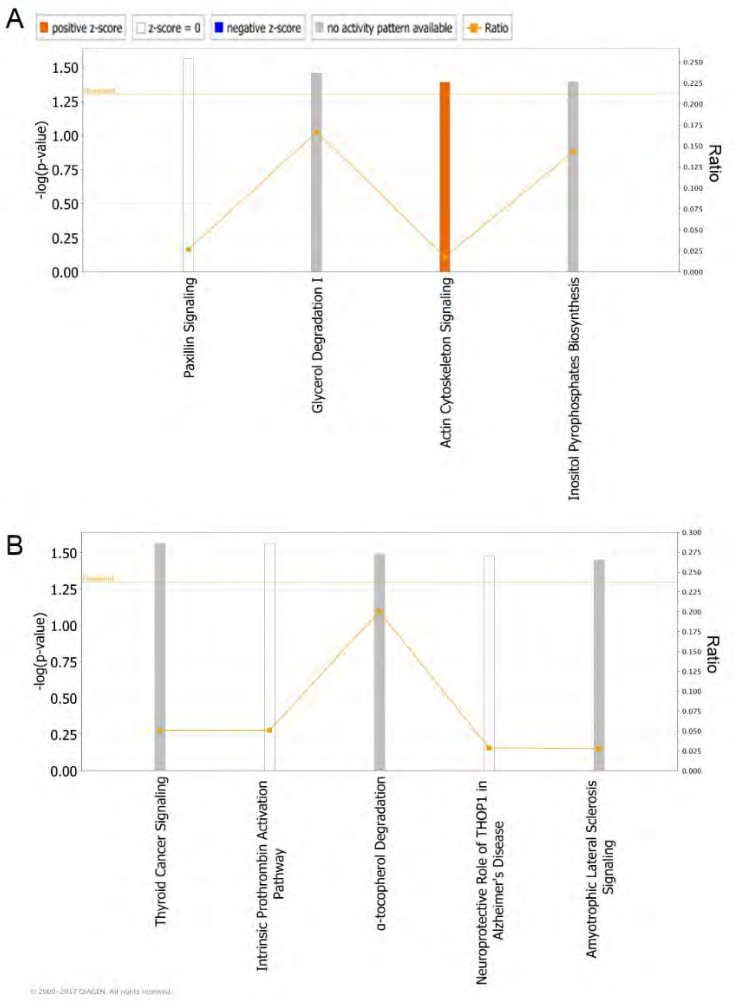

Supplementary Figure 6. Significantly enriched (p value<0.05; Fisher's exact test) Ingenuity canonical pathways. The x-axis represents significant canonical pathways while the left Y-axis displays the -log of p-value which is calculated by Fisher's exact test right-tailed. Thus taller bars equate to increased significance. The threshold is set at -log of 0.05 (p value) so everything above or at that threshold denotes significant change. The overall activation/inhibition states of canonical pathways are predicted based on a Z-score algorithm. The canonical pathway bar charts are colored to indicate their activation z-scores. Orange bars predict an overall increase in the activity of the pathway while blue bars indicate a prediction of an overall decrease in activity. White bars are those with a z-score which is zero or very close to 0, predicting unchanged overall activity of the pathway. Gray bars indicate pathways that are currently ineligible for a prediction. The orange points connected by a thin line represent the Ratio (right Y-axis). The ratio is calculated as the number of genes in a given pathway that meet cutoff criteria, divided by the total number of genes that make up that pathway and that are in the reference gene set. Panel A: Het-CKO vs. Control heart samples at 2-weeks-of-age. Panel B: Het-CKO vs. Control heart samples at 3-weeks-of-age.

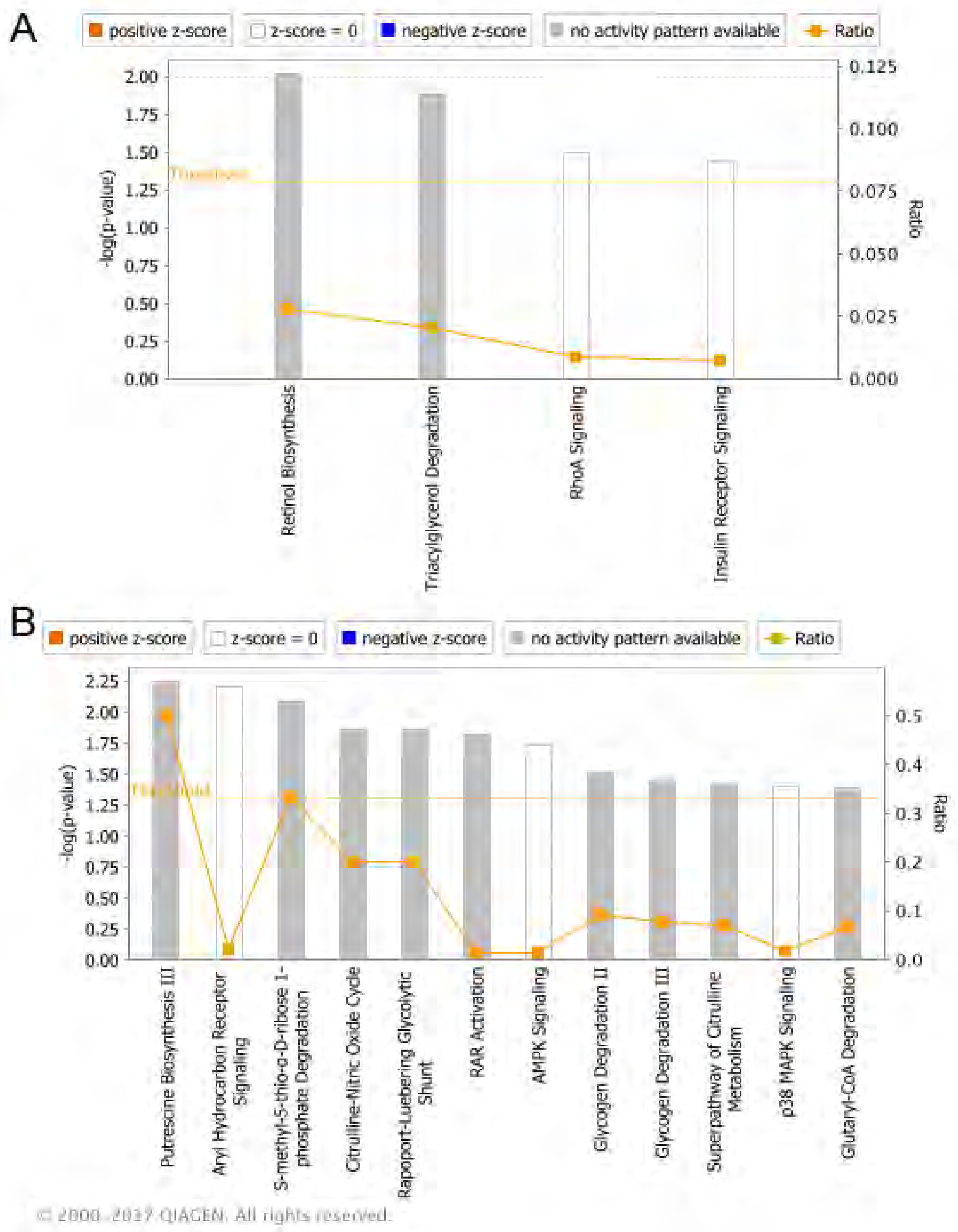

Supplementary Figure 7. Significantly enriched (*p*-value<0.05; Fisher's exact test) Ingenuity canonical pathways. The x-axis represents significant canonical pathways while the left Y-axis displays the -log of *p*-value which is calculated by Fisher's exact test right-tailed. The overall activation/inhibition states of canonical pathways are predicted based on a Z-score algorithm. The canonical pathway bar charts are colored to indicate their activation z-scores. White bars are those with a z-score which is zero or very close to 0, predicting unchanged overall activity of the pathway. Gray bars indicate pathways that are currently ineligible for a prediction. The orange points connected by a thin line represent the Ratio (right Y-axis). Panel A: Comparison between 3-weeks and 2-weeks in Control mice. Panel B: Comparison between 3-weeks and 2-weeks in Het-CKO mice.

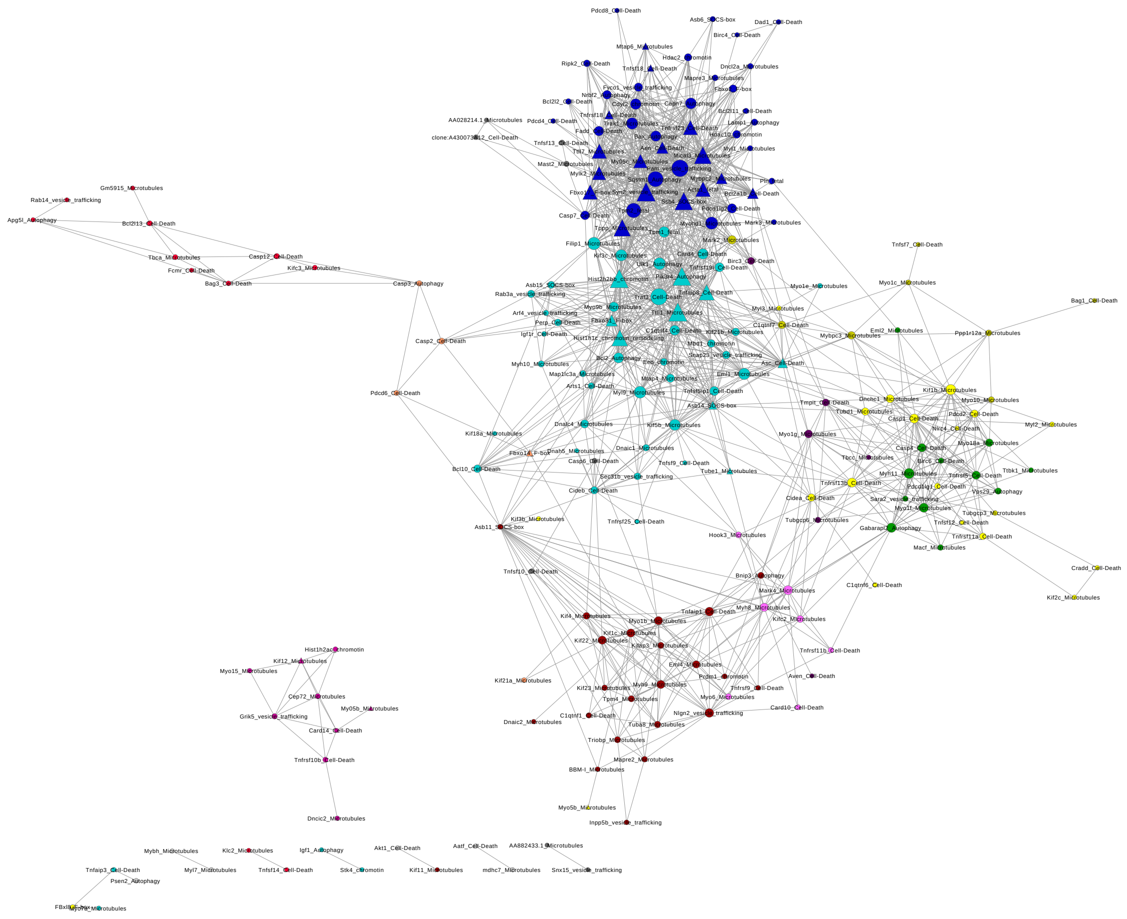

Supplementary figure 8. Pathway gene weighted co-expression network. The microarray data of hand-curated genes from seven sets of pathways was used as an input to construct a pathway genes network where mod-ules (densely connected genes) were identified using R package WGCNA. In this co-expression network, node represents a gene, and statistically significant and non-significant genes were presented as triangle and circle respectively. Node size is proportional to the number of its neighbour genes.   Genes with the same colour belong to the same module. Different modules in pathway interaction network map represents different pattern of gene expression. A pair of genes were connected by an edge if their co-expression correlation is bigger than 0.75.

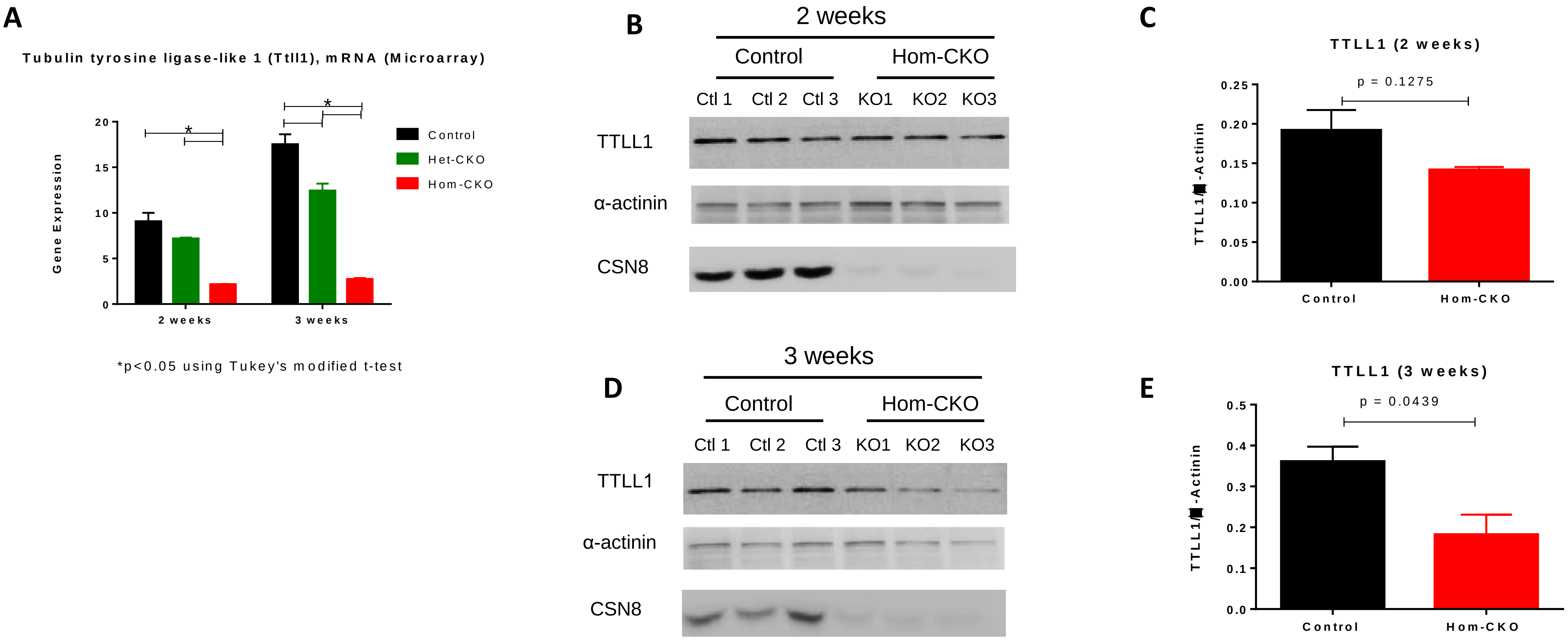

Supplementary Figure 9. Effect of CSN8-CKO on TTLL1 gene and protein expression. Panel A. Differential gene expression of TTLL1 genes in CTL, Het-CKO, and Hom-CKO littermate mice at 2- and 3-weeks-of-age (n=3). The data are presented as means ± SEM. Statistical significance among the groups was determined by ANOVA followed by Tukey's modified Student's t-test as the post hoc test. All findings were considered statistically significant with *P<0.05. Panel B. Representative western blot image for the indicated proteins in myocardium from mice at 2-weeks-of-age. α-actinin was probed as loading control. Panel C. Summary of the densitometric data of FBXO31 from the experiments as illustrated in A. *p=0.1275 using Student's t-test. Panel D. Representative western blot image for the indicated proteins in myocardium from mice at 3-weeks-of-age. α-actinin was probed as loading control. Panel E. Summary of the densitometric data of FBXO31 from the experiments as illustrated in C.*p=0.0439 using Student's t-test.

Supplementary Table 1. **List of DEGs that are common among three comparisons: Het-CKO vs. Hom-CKO, Control vs. Hom-CKO, Control vs. Het-CKO at 3-weeks-of-age**

No.	Accession #	Gene	Description	Function
1	L06234.1	*cacna1s*	calcium channel, voltage-dependent, L type, alpha 1S subunit	Calcium channel activity, Adrenergic signaling in cardiomyocytes, Cardiac muscle contraction
2	NM_008966.2	*ptgfr*	Prostaglandin F receptor	Protein encoded by this gene is a receptor for prostaglandin F2-alpha (PGF2-alpha),
3	AK079396.1	*mylk2*	Myosin light polypeptide kinase 2, skeletal muscle	This gene encodes a myosin light chain kinase, a calcium/calmodulin dependent enzyme that is expressed in adult skeletal muscle
4	AK005570.1	*ppp1r3g*	protein phosphatase 1, regulatory (inhibitor) subunit 3	Glycogen binding and protein phosphatase binding
5	AI429307.1	NA	mm45d08x1 Stratagene mouse melanoma (#937312) cDNA clone IMAGE:524463 3'.	EST

Supplementary table 2. List of hand-curated “ATP Synthase” genes used for Fisher’s Exact test

Substance	ACCN#	Description	Gene Symbol	(Gene expression) 2 weeks						(Gene expression) 3 weeks					
				CTL		Hom-CKO		Het-CKO		CTL		Hom-CKO		Het-CKO	
				Average	SD	Average	SD	Average	SD	Average	SD	Average	SD	Average	SD
GE38704	AA672840.1	vu05a09r1 Soares_mammary_gland_NbMMG cDNA clone IMAGE:1179736 5' similar to SW:ATPO_RAT Q06647 ATP SYNTHASE OLIGOMYCIN SENSITIVITY CONFERRAL PROTEIN PRECURSOR, MITOCHONDRIAL ;.	Atp5o	326.83	33.29	220.58	23.06	267.84	15.53	432.64	26.07	254.31	16.47	369.95	33.51
GE1440248	NM_175015.1	ATP synthase, H+ transporting, mitochondrial F0 complex, subunit c (subunit 9), isoform 3 (Atp5g3)	Atp5g3	457.51	39.70	360.36	45.66	371.07	23.48	539.88	34.41	378.86	7.76	461.69	26.11
GE131232	NM_145427.1	ATP synthase mitochondrial F1 complex assembly factor 2 (Atpaf2)	Atpaf2	8.73	0.64	8.59	0.50	8.21	0.70	13.99	1.20	8.83	0.59	9.33	0.15
GE42874	NM_026468.1	ATP synthase, H+ transporting, mitochondrial F0 complex, subunit c (subunit 9), isoform 2 (Atp5g2)	Atp5g2	104.90	20.37	83.08	6.56	85.79	4.84	131.22	17.09	75.64	5.90	98.69	18.75
GE121228	NM_025313.1	ATP synthase, H+ transporting, mitochondrial F1 complex, delta subunit (Atp5d)	Atp5d	256.73	24.25	225.52	22.67	230.69	9.51	347.52	30.39	261.22	13.40	321.58	35.45
GE42461	NM_007506.2	ATP synthase, H+ transporting, mitochondrial F0 complex, subunit c (subunit 9), isoform 1 (Atp5g1)	Atp5g1	217.02	44.76	167.30	10.09	191.17	3.53	277.45	37.73	186.81	1.09	251.91	20.22
GE40635	NM_020615.2	ATP synthase, H+ transporting, mitochondrial F1 complex, gamma polypeptide 1 (Atp5c1)	Atp5c1	179.62	32.31	136.84	3.06	150.12	4.65	267.02	38.65	166.42	19.36	213.69	26.11
GE1488901	NM_025983.2	ATP synthase, H+ transporting, mitochondrial F1 complex, epsilon subunit (Atp5e)	Atp5e	290.70	99.16	193.43	21.71	204.67	9.50	430.93	90.52	217.71	15.74	299.54	45.01
GE34769	NM_007505.1	ATP synthase, H+ transporting, mitochondrial F1 complex, alpha subunit, isoform 1 (Atp5a1)	Atp5a1	232.43	25.50	189.30	9.28	202.96	5.64	323.62	58.46	218.89	15.46	263.46	4.16
GE41191	AI325711.1	mm85h05y1 Stratagene mouse embryonic carcinomaRA (#937318) cDNA clone IMAGE:535257 5' similar to gb:L01062 Mouse ATP synthase alpha subunit, complete cds (MOUSE);.	Atp5a1	61.23	3.14	48.53	6.94	52.18	2.73	87.80	15.19	66.36	6.15	68.92	3.38
GE42741	NM_016774.2	ATP synthase, H+ transporting mitochondrial F1 complex, beta subunit (Atp5b)	Atp5b	161.28	16.05	135.39	9.58	138.14	4.93	237.26	76.37	140.53	17.61	159.23	10.71
GE1497595	AI506322.1	vq61b02x1 Barstead mouse irradiated colon MPLRB7 cDNA clone IMAGE:1106763 3' similar to gb:X03559 ATP SYNTHASE BETA CHAIN, MITOCHONDRIAL PRECURSOR (HUMAN);.	EST	3.00	0.52	3.26	0.25	3.80	0.18	2.43	0.30	3.02	0.54	3.32	0.18
GE102532	NM_145427.1	ATP synthase mitochondrial F1 complex assembly factor 2 (Atpaf2)	Atpaf2	9.59	0.75	9.83	0.23	9.49	0.68	13.12	1.87	12.00	0.47	10.13	0.65
GE1388523	AA672840.1	vu05a09r1 Soares_mammary_gland_NbMMG cDNA clone IMAGE:1179736 5' similar to SW:ATPO_RAT Q06647 ATP SYNTHASE OLIGOMYCIN SENSITIVITY CONFERRAL PROTEIN PRECURSOR, MITOCHONDRIAL ;.	Atp5o	0.17	0.11	0.10	0.08	0.17	0.10	0.18	0.03	0.13	0.09	0.28	0.12
GE1389716	AY517482.1	putative osteoclast-specific vacuolar ATP synthase mRNA	mRNA	0.10	0.10	0.05	0.04	0.06	0.06	0.14	0.14	0.16	0.11	0.09	0.11

Supplementary table 3. List of hand-curated "SOCS-box" genes used for Fisher's Exact test

Substance	ACCN#	Description	Gene Symbol	(Gene expression) 2 weeks						(Gene expression) 3 weeks					
				CTL		Hom-CKO		Het-CKO		CTL		Hom-CKO		Het-CKO	
				Average	SD	Average	SD	Average	SD	Average	SD	Average	SD	Average	SD
GE126471	NM_080856.1	ankyrin repeat and SOCS box-containing protein 14	Asb14	9.20	1.25	6.49	0.39	7.18	0.43	18.29	1.06	6.78	0.89	10.84	1.06
GE126271	NM_080444.2	ankyrin repeat and SOCS box-containing protein 10	Asb10	4.54	0.67	4.30	0.60	4.78	0.11	7.79	2.05	4.49	0.67	5.99	0.96
GE126442	NM_080847.1	ankyrin repeat and SOCS box-containing protein 15	Asb15	3.01	0.39	2.76	0.20	3.72	0.10	4.35	1.07	2.54	0.12	3.87	0.20
GE126482	NM_080858.1	ankyrin repeat and SOCS box-containing protein 12	Asb12	4.70	0.40	4.55	1.01	4.54	0.33	5.83	0.41	3.61	0.21	4.71	0.28
GE42191	NM_023048.4	ankyrin repeat and SOCS box-containing protein 4	Asb4	1.04	0.13	0.97	0.07	0.92	0.08	1.11	0.18	0.74	0.05	0.95	0.08
GE42879	NM_026853.1	ankyrin repeat and SOCS box-containing protein 11	Asb11	23.39	0.93	18.87	1.13	20.20	2.27	39.51	2.81	26.74	1.47	34.55	1.28
GE126475	NM_080857.1	ankyrin repeat and SOCS box-containing protein 13	Asb13	3.25	0.43	3.40	0.11	3.25	0.07	4.30	0.46	3.10	0.18	3.53	0.11
GE37607	NM_019654.2	suppressor of cytokine signaling 5	Socs5	1.21	0.24	1.33	0.19	1.15	0.08	1.54	0.52	1.13	0.07	1.01	0.02
GE41870	NM_030121.2	ankyrin repeat and SOCS box-containing protein 8	Asb8	15.26	0.90	14.38	0.24	14.21	0.99	18.70	2.73	13.99	1.35	14.92	1.52
GE35214	NM_009896.1	suppressor of cytokine signaling 1	Socs1	1.29	0.46	0.98	0.17	1.06	0.08	1.42	0.10	1.11	0.10	1.01	0.26
GE128967	NM_138657.1	suppressor of cytokine signaling 7	Socks7	5.06	1.33	4.80	0.18	6.36	0.50	6.98	1.69	5.68	0.55	8.17	2.09
GE33347	NM_023906.2	ankyrin repeat and SOCS box-containing protein 3	Asb3	3.95	0.55	3.44	0.18	3.17	0.12	4.16	0.38	3.49	0.24	3.15	0.16
GE38428	NM_023049.1	ankyrin repeat and SOCS box-containing protein 2	Asb2	51.96	13.70	55.57	2.46	51.75	5.37	58.91	13.12	51.00	4.91	50.24	10.90
GE42207	NM_080443.1	ankyrin repeat and SOCS box-containing protein 7	Asb7	0.55	0.08	0.56	0.00	0.49	0.15	0.47	0.14	0.42	0.05	0.42	0.04
GE33504	NM_007707.2	suppressor of cytokine signaling 3	Socs3	0.96	0.07	0.73	0.09	0.81	0.01	1.05	0.09	0.98	0.06	0.86	0.15
GE126427	NM_080843.2	suppressor of cytokine signaling 4, mRNA	Socs4	0.99	0.08	1.05	0.17	0.85	0.04	0.83	0.17	0.79	0.06	0.76	0.11
GE39518	NM_133346.1	ankyrin repeat and SOCS box-containing protein 6	Asb6	1.75	0.16	2.01	0.12	1.60	0.16	2.32	0.35	2.27	0.15	1.78	0.13
GE1396515	NM_021539.3	WD repeat and SOCS box-containing 2	Wsb2	27.35	4.93	24.77	3.56	22.28	1.05	29.43	5.98	31.15	1.68	28.31	5.22
GE38833	NM_027027.1	ankyrin repeat and SOCS box-containing protein 9	Asb9	0.09	0.01	0.09	0.03	0.10	0.04	0.06	0.05	0.07	0.06	0.08	0.08
GE136395	NM_148953.1	ankyrin repeat and SOCS box-containing 16	Asb16	0.19	0.09	0.16	0.07	0.22	0.09	0.13	0.05	0.14	0.03	0.24	0.04
GE125114	NM_029569.1	ankyrin repeat and SOCs box-containing protein 5	Asb5	0.13	0.10	0.10	0.01	0.16	0.04	0.17	0.17	0.19	0.05	0.19	0.09
GE42767	NM_018821.2	suppressor of cytokine signaling 6	Socs6	5.43	0.26	6.04	0.65	5.01	0.48	5.43	1.28	6.19	0.19	5.71	0.84
GE1392847	NM_178236.2	ankyrin repeat and SOCS box-containing protein 7	Asb7	0.48	0.07	0.61	0.09	0.49	0.07	0.55	0.03	0.71	0.11	0.50	0.05
GE1405061	NM_019653.2	WD repeat and SOCS box-containing 1	Wsb1	0.14	0.12	0.22	0.02	0.18	0.04	0.12	0.04	0.19	0.11	0.10	0.01
GE129313	NM_139152.1	ankyrin repeat and SOCS box-containing 18	Asb18	0.43	0.05	0.44	0.06	0.46	0.05	0.46	0.16	0.72	0.03	0.66	0.12
GE130665	NM_145134.2	SPRY domain-containing SOCS box 4	Ssb4	9.95	0.59	16.20	1.85	9.00	0.13	12.12	3.61	26.37	5.31	10.67	2.56

Supplementary table 4. List of hand-curated “F-box” genes used for Fisher’s Exact test

Substance	ACCN#	Description	Gene Symbol	(Gene expression) 2 weeks						(Gene expression) 3 weeks					
				CTL		Hom-CKO		Het-CKO		CTL		Hom-CKO		Het-CKO	
				Average	SD	Average	SD	Average	SD	Average	SD	Average	SD	Average	SD
GE38412	AK003388.1	F-Box protein 31	Fbxo31	4.92	0.97	3.04	0.29	4.78	0.14	7.45	0.81	3.26	0.32	5.95	0.65
GE127205	NM_133765.2	F-box only protein 14	Fbxo14	1.85	0.21	1.36	0.10	1.94	0.06	3.26	0.63	1.54	0.11	2.37	0.34
GE42200	NM_080428.2	F-box and WD-40 domain protein 7, archipelago homolog (Drosophila)	Fbxw7	15.23	2.96	10.75	0.76	15.89	0.77	15.33	2.07	9.85	0.49	15.35	2.09
GE33383	AF176529.1	F-box protein mRNA	FBX13	3.34	0.34	2.99	0.19	3.13	0.10	3.85	0.67	2.63	0.06	3.16	0.31
GE36605	NM_013911.1	F-box and leucine-rich repeat protein 12	Fbxl12	7.30	0.63	6.28	0.08	6.06	0.24	6.77	0.71	4.90	0.39	5.58	0.16
GE102360	NM_133765.2	F-box only protein 14	Fbxo14	9.00	0.30	6.84	0.32	8.68	1.19	12.70	0.97	9.44	0.46	10.80	1.53
GE36554	CF197790.1	maj40a04y1 McCarrey Eddy 18 day preleptotene spermatocytes cDNA clone IMAGE:6943952 5' similar to TR:Q9UKC0 Q9UKC0 F-BOX PROTEIN FBX22	Fbxo22	2.41	0.19	2.31	0.29	2.42	0.16	3.19	0.66	2.40	0.29	2.70	0.12
GE37844	NM_020593.2	F-box only protein 3, transcript variant 2	Fbxo3	20.90	1.14	18.27	1.51	17.08	0.61	21.61	2.87	16.95	0.75	18.21	0.21
GE1515174	NM_030236.1	F-box only protein 34	Fbxo34	0.34	0.06	0.21	0.15	0.31	0.06	0.41	0.18	0.32	0.11	0.17	0.04
GE1474427	NM_175530.2	F-box only protein 34-like	Fbxo34l	9.43	1.21	8.69	1.02	10.97	0.44	10.82	1.66	9.11	0.45	10.93	1.70
GE125575	NM_030236.1	F-box only protein 34	Fbxo34	5.70	0.22	5.60	0.50	5.43	0.21	5.98	0.82	5.13	0.45	4.70	0.25
GE38381	NM_133694.1	F-box only protein 37	Fbxo37	4.14	0.35	3.63	0.16	3.76	0.19	4.03	0.16	3.51	0.14	3.63	0.24
GE36884	NM_015821.1	F-box and leucine-rich repeat protein 8	Fbxl8	2.03	0.12	1.75	0.04	1.76	0.22	2.24	0.15	1.96	0.04	1.74	0.12
GE42727	NM_013890.2	F-box and WD-40 domain protein 2	Fbxw2	12.25	0.59	12.77	1.14	11.33	0.49	13.88	8.02	12.38	1.16	11.46	0.66
GE131918	NM_145564.2	F-box only protein 21	Fbxo21	14.04	1.04	12.18	0.63	12.68	0.52	12.03	0.26	10.82	0.10	10.98	0.46
GE136712	NM_153195.1	F-box only protein 7	Fbxo7	17.90	1.47	17.58	1.56	19.19	1.02	20.48	1.01	18.55	1.47	19.72	2.90
GE36606	NM_013909.1	F-box and leucine-rich repeat protein 6	Fbxl6	2.82	0.09	2.79	0.19	2.59	0.16	3.22	0.26	2.96	0.33	3.19	0.32
GE39729	NM_145564.2	F-box only protein 21	Fbxo21	7.73	1.11	6.86	0.27	7.12	0.06	5.78	0.45	5.33	0.24	5.89	0.45
GE1493140	NM_177076.2	F-box and leucine-rich repeat protein 13	Fbxl13	0.62	0.10	0.57	0.05	0.49	0.01	0.53	0.14	0.49	0.11	0.46	0.12
GE36887	NM_015797.1	F-box only protein 6b	Fbxo6b	9.05	1.40	8.61	0.72	8.78	0.18	13.92	1.78	12.87	0.61	10.73	0.34
GE33381	NM_015822.1	F-box and leucine-rich repeat protein 3a	Fbxl3a	2.70	0.41	2.40	0.35	2.34	0.34	2.46	0.58	2.32	0.20	2.28	0.20
GE127682	NM_133940.1	F-box and leucine-rich repeat protein 14	Fbxl14	3.61	0.36	3.55	0.33	4.31	0.17	4.60	0.45	4.36	0.16	4.72	0.31
GE33375	NM_015792.1	F-box only protein 18	Fbxo18	4.36	0.44	4.35	0.19	3.89	0.25	4.81	1.08	4.56	0.16	4.05	0.67
GE123880	NM_028049.1	F-box only protein 22	Fbxo22	4.65	0.96	4.20	0.87	3.80	0.22	5.61	1.13	5.41	0.26	4.90	0.45
GE36885	NM_015795.1	F-box only protein 16	Fbxo16	1.43	0.08	1.35	0.19	1.65	0.09	1.28	0.10	1.24	0.09	1.55	0.13
GE36607	NM_013908.1	F-box and WD-40 domain protein 5	Fbxw5	11.05	2.04	12.99	0.63	11.61	0.22	14.83	3.59	14.84	2.07	12.88	3.72
GE40940	NM_025785.1	F-box only protein 25	Fbxo25	4.67	0.23	5.20	0.27	4.49	0.33	5.30	0.29	5.33	0.23	4.20	0.33
GE121905	NM_025995.1	F-box only protein 5	Fbxo5	3.49	0.91	3.59	0.42	2.62	0.52	1.72	0.50	1.75	0.41	1.94	0.63
GE119869	NM_015791.2	F-box only protein 8	Fbxo8	12.93	1.74	12.51	0.15	10.16	0.59	14.94	1.61	15.65	0.21	11.63	1.32
GE40472	NM_023605.1	f-box only protein 9	Fbxo9	16.51	1.37	16.05	0.50	14.52	0.65	16.07	1.37	17.39	1.53	13.98	0.99
GE121308	NM_025386.1	F-box only protein 36	Fbxo36	1.93	0.06	2.11	0.16	2.25	0.16	2.10	0.21	2.32	0.06	1.99	0.14
GE113472	NM_134136.1	F-box protein 38	Fbxo38	7.41	0.35	7.52	0.35	6.91	0.18	6.15	0.51	6.83	0.57	5.51	0.34
GE127918	NM_134015.1	F-box and WD-40 domain protein 1B	Fbxw1b	12.73	0.66	14.42	0.58	12.61	1.08	13.12	2.37	16.00	1.18	12.30	1.18
GE39300	NM_028841.1	F-box only protein 23	Fbxo23	2.41	0.32	2.96	0.21	2.29	0.30	2.42	0.22	3.18	0.18	2.17	0.16
GE1565035	NM_172988.1	F-box and leucine-rich repeat protein 4	Fbxl4	0.50	0.18	0.43	0.01	0.40	0.08	0.39	0.03	0.52	0.09	0.42	0.11
GE114271	NM_176848.1	F-box only protein 2	Fbxo2	0.96	0.16	1.27	0.09	1.29	0.05	1.20	0.08	1.63	0.04	1.27	0.04
GE103087	NM_026791.1	F-box and WD-40 domain protein 9	Fbxw9	4.92	0.97	4.82	0.47	3.73	0.14	3.92	0.85	5.34	0.76	3.19	0.63
GE39726	NM_178624.2	F-box and leucine-rich repeat protein 2	Fbxl2	0.52	0.02	0.56	0.02	0.43	0.05	0.41	0.09	0.58	0.02	0.46	0.09
GE41204	NM_026346.1	F-box only protein 32	Fbxo32	10.59	2.00	14.37	0.77	10.38	1.35	11.98	3.41	17.66	3.55	10.26	3.47
GE36886	NM_015796.1	F-box only protein 17	Fbxo17	1.19	0.08	1.52	0.27	1.14	0.08	1.28	0.09	2.38	0.21	1.10	0.14

Supplementary-table 5. List of hand-curated “Chromatin Remodeling” genes used for Fisher’s Exact test

				(Gene expression) 2 weeks						(Gene expression) 3 weeks					
Substance	ACCN#	Description	Gene Symbol	CTL		Hom-CKO		Het-CKO		CTL		Hom-CKO		Het-CKO	
				Average	SD	Average	SD	Average	SD	Average	SD	Average	SD	Average	SD
GE1547270	NM_011417.1	SWI/SNF related, matrix associated, actin dependent regulator of chromatin, subfamily a, member 4	Smarca4	6.83	0.89	6.73	0.48	6.46	0.66	7.78	1.30	6.82	0.12	7.57	1.30
GE42858	NM_025891.2	SWI/SNF related, matrix associated, actin dependent regulator of chromatin, subfamily d, member 3	Smarcd3	23.53	0.77	20.81	0.64	22.19	1.40	23.98	1.69	19.80	2.86	20.80	3.11
GE34559	NM_008229.1	histone deacetylase 2 (Hdac2)	Hdac2	14.48	3.07	11.73	0.84	12.53	0.27	10.89	2.03	9.03	0.40	12.30	0.20
GE1575036	AF325921.1	DNA-dependent ATPase SNF2H mRNA	Snf2h	0.09	0.04	0.08	0.01	0.04	0.01	0.06	0.05	0.09	0.04	0.10	0.07
GE137741	NM_172562.1	transcriptional adaptor 2 (ADA2 homolog, yeast)-like	Tada2l	3.80	0.07	3.35	0.12	3.53	0.27	4.19	0.60	3.50	0.35	3.94	0.26
GE42692	NM_011842.2	metastasis-associated gene family, member 2	Mta2	9.66	1.00	9.05	0.45	8.07	0.27	8.42	0.92	7.19	0.05	6.64	0.39
GE42124	NM_054081.1	metastasis associated 1	Mta1	8.51	1.01	7.06	0.31	7.55	0.37	10.31	1.26	8.95	0.14	10.04	0.60
GE36686	NM_013595.1	methyl-CpG binding domain protein 3	Mbd3	13.39	1.99	12.97	0.99	11.53	0.48	16.47	3.49	12.11	0.47	13.11	0.58
GE1071700	NM_010773.1	methyl-CpG binding domain protein 2	Mbd2	17.41	1.02	15.50	0.28	13.59	0.52	19.24	0.28	15.24	1.77	14.92	1.24
GE102608	NM_144919.1	histone deacetylase 11	Hdac11	1.17	0.18	0.87	0.09	1.17	0.05	1.84	0.24	0.66	0.08	1.36	0.22
GE37631	NM_015786.1	histone 1, H1c	Hist1h1c	6.39	0.93	2.88	0.51	5.30	0.43	9.91	0.80	3.61	0.12	8.38	0.92
GE1421533	NM_013548.2	histone 1, H3f	Hist1h3f	0.30	0.01	0.25	0.07	0.24	0.07	0.42	0.08	0.16	0.12	0.18	0.06
GE1456776	NM_175666.1	histone 2, H2bb	Hist2h2bb	2.63	0.31	1.67	0.17	2.50	0.14	3.09	0.42	1.53	0.11	3.04	0.24
GE41468	NM_015787.1	histone 1, H1e	Hist1h1e	0.84	0.07	0.74	0.07	0.82	0.02	1.16	0.16	0.65	0.10	0.98	0.04
GE136691	NM_153173.1	histone 1, H4h	Hist1h4h	3.49	0.87	2.61	0.31	2.78	0.27	5.00	0.69	3.12	0.24	4.46	1.06
GE1392091	NM_199198.1	histone deacetylase 10	Hdac10	1.74	0.15	1.58	0.16	1.57	0.10	1.71	0.02	1.29	0.06	1.61	0.21
GE1543365	AF319621.1	histone acetylase complex subunit MRG15-2 mRNA, complete cds, alternatively spliced	Morf4l1	38.68	7.30	28.87	5.60	30.97	1.75	48.02	4.31	36.41	3.85	40.27	3.50
GE36646	NM_175655.1	histone 1, H4f (Hist1h4f), mRNA	Hist1h4f	0.57	0.18	0.56	0.08	0.52	0.01	0.70	0.06	0.55	0.04	0.57	0.13
GE36466	NM_010411.1	histone deacetylase 3	Hdac3	10.24	0.36	10.03	0.33	9.17	0.34	12.12	2.04	9.80	0.80	9.28	0.33
GE35519	NM_010377.1	histone 1, H1t	Hist1h1t	0.39	0.05	0.34	0.04	0.33	0.03	0.45	0.03	0.37	0.07	0.38	0.14
GE34559	NM_008229.1	histone deacetylase 2	Hdac2	14.48	3.07	11.73	0.84	12.53	0.27	10.89	2.03	9.03	0.40	12.30	0.20
GE1430202	NM_175658.1	histone 1, H2aa mRNA	Hist1h2aa	0.02	0.01	0.07	0.04	0.09	0.08	0.10	0.06	0.09	0.05	0.13	0.07
GE125459	NM_030082.1	histone 3, H2ba	Hist3h2ba	0.60	0.19	0.40	0.14	0.45	0.01	0.96	0.09	0.89	0.16	0.87	0.15
GE36368	NM_010412.2	histone deacetylase 5	Hdac5	22.17	3.85	22.86	1.04	21.92	1.64	25.42	2.69	24.31	1.48	21.64	2.50
GE112532	BC006883.1	SNF2 histone linker PHD RING helicase, mRNA (cDNA clone IMAGE:3596125)	Shprh	3.67	0.43	3.49	0.36	3.51	0.17	3.03	0.77	3.00	0.17	3.16	0.21
GE41906	NM_012015.1	H2A histone family, member Y	H2afy	13.41	1.27	13.23	1.17	12.04	0.80	17.99	2.78	18.66	0.44	14.52	1.33
GE1536668	NM_023422.1	histone 1, H2bc	Hist1h2bc	19.58	5.23	18.45	1.64	16.71	0.93	29.46	3.10	31.20	0.44	27.52	3.44
GE128840	NM_138311.1	H1 histone family, member O, oocyte-specific	H1foo	0.70	0.08	0.66	0.04	0.81	0.10	0.67	0.05	0.71	0.10	0.74	0.05
GE1456715	NM_178194.2	histone 1, H2be	Hist1h2be	0.23	0.07	0.10	0.03	0.09	0.06	0.15	0.08	0.15	0.05	0.22	0.07
GE1442879	NM_178189.2	histone 1, H2ac	Hist1h2ac	1.64	0.20	1.89	0.21	2.64	0.26	1.88	0.10	2.06	0.15	2.31	0.09
GE42763	NM_017479.1	MYST histone acetyltransferase monocytic leukemia 4	Myst4	2.41	0.55	2.29	0.58	1.96	0.32	2.10	0.14	2.34	0.22	2.23	0.23
GE125615	NM_030609.1	histone 1, H1a, mRNA	Hist1h1a	0.19	0.04	0.14	0.05	0.15	0.09	0.10	0.11	0.11	0.04	0.24	0.06
GE1400915	BC011440.1	histone 1, H2bc, mRNA (cDNA clone MGC:19269 IMAGE:3989862)	Hist2h2bc	0.18	0.02	0.21	0.03	0.14	0.07	0.14	0.03	0.16	0.06	0.14	0.02
GE1411041	NM_178196.2	histone 1, H2bg	Hist1h2bg	0.21	0.10	0.11	0.10	0.09	0.09	0.19	0.10	0.23	0.08	0.14	0.06
GE130606	NM_145073.1	histone 1, H3g	Hist1h3g	0.28	0.12	0.48	0.10	0.43	0.14	0.48	0.08	0.59	0.06	0.57	0.14
GE37450	NM_019572.2	histone deacetylase 7A	Hdac7a	2.79	0.32	2.99	0.37	2.93	0.15	1.85	0.25	2.35	0.16	1.82	0.28
GE36369	NM_010413.2	histone deacetylase 6	Hdac6	3.14	0.39	3.29	0.36	3.13	0.25	2.52	0.28	3.23	0.47	2.46	0.23
GE1424326	BC057955.1	histone 1, H4h, mRNA (cDNA clone IMAGE:5359141), with apparent retained intron	Hist1h4c	0.14	0.06	0.17	0.00	0.07	0.02	0.13	0.02	0.20	0.05	0.13	0.02
GE1507826	BC055904.1	histone 2, H3c2, mRNA (cDNA clone IMAGE:4913108)	Hist2h3c2	0.64	0.14	0.76	0.07	0.52	0.05	0.74	0.24	1.44	0.23	0.99	0.20
GE36278	NM_011714.1	bromodomain adjacent to zinc finger domain, 1B	Baz1b	2.45	0.39	2.48	0.22	2.56	0.08	2.29	0.43	1.90	0.06	2.12	0.28
GE35203	NM_009881.1	chromodomain protein, Y chromosome-like	Cdyl	2.03	0.09	2.16	0.16	1.77	0.18	1.93	0.25	1.93	0.25	1.53	0.03
GE1419445	NM_054078.1	bromodomain adjacent to zinc finger domain, 2A	Baz2a	1.89	0.18	1.80	0.20	1.57	0.14	1.10	0.17	1.48	0.14	1.34	0.17
GE42644	NM_010788.1	methyl CpG binding protein 2	Mecp2	5.06	0.51	4.78	0.51	4.36	0.42	3.76	0.36	4.06	0.94	3.90	0.37
GE41921	NM_033566.1	AT rich interactive domain 1A (Swi1 like)	Arid1a	7.34	0.59	8.36	0.49	7.46	0.60	7.19	0.92	7.24	0.19	6.10	0.44
GE39850	NM_175251.1	AT rich interactive domain 2 (Arid-rfx like)	Arid2	23.88	1.50	23.89	1.91	21.16	1.76	19.32	1.16	19.53	0.93	18.79	4.96
GE40030	NM_053124.2	SWI/SNF related, matrix associated, actin dependent regulator of chromatin, subfamily a, member 5	Smarca5	7.25	2.92	5.61	0.94	6.18	0.26	6.63	0.64	6.43	1.80	6.56	0.56
GE42708	NM_013502.1	C-terminal binding protein 1 (Ctbp1)	Ctbp1	38.29	2.75	35.87	1.83	34.48	1.71	43.26	2.50	33.43	1.60	37.64	4.89
GE38087	NM_021876.1	embryonic ectoderm development	Eed	6.14	0.96	5.39	0.30	5.53	0.22	7.03	0.63	5.62	0.17	6.50	0.24
GE1546389	NM_007971.1	enhancer of zeste homolog 2 (Drosophila)	Ezh2	1.98	0.48	1.71	0.47	1.52	0.17	1.38	0.12	1.07	0.26	1.36	0.19
GE34989	NM_007905.1	polyhomeotic-like 1 (Drosophila)	Phc1	2.83	0.09	3.58	0.55	2.71	0.19	3.02	0.27	3.04	0.15	2.71	0.07
GE1562545	NM_011277.1	ring finger protein 2	Rnf2	1.10	0.21	1.03	0.07	1.05	0.14	0.85	0.15	0.92	0.18	0.98	0.11
GE33563	NM_009054.1	tripartite motif protein 27	Trim27	2.29	0.27	2.24	0.30	1.95	0.08	2.28	0.23	2.07	0.12	2.07	0.32
GE33477	NM_007622.2	chromobox homolog 1 (Drosophila HP1 beta)	Cbx1	12.09	0.68	13.31	1.63	11.27	0.54	11.34	0.08	9.89	0.59	12.66	0.54

Supplementary-table 5. Cont'd….

Substance	ACCN#	Description	Gene Symbol	(Gene expression) 2 weeks						(Gene expression) 3 weeks					
				CTL		Hom-CKO		Het-CKO		CTL		Hom-CKO		Het-CKO	
				Average	SD	Average	SD	Average	SD	Average	SD	Average	SD	Average	SD
GE33475	NM_007623.2	chromobox homolog 2 (Drosophila Pc class)	Cbx2	0.41	0.04	0.28	0.19	0.41	0.02	0.36	0.09	0.18	0.04	0.30	0.11
GE34817	NM_007625.1	chromobox homolog 4 (Drosophila Pc class)	Cbx4	3.15	0.66	3.19	0.21	3.26	0.13	2.88	0.56	3.06	0.33	2.76	0.52
GE33476	NM_007626.2	chromobox homolog 5 (Drosophila HP1a)	Cbx5	0.60	0.12	0.45	0.06	0.44	0.06	0.64	0.18	0.37	0.07	0.61	0.05
GE36278	NM_011714.1	bromodomain adjacent to zinc finger domain, 1B	Baz1b	2.45	0.39	2.48	0.22	2.56	0.08	2.29	0.43	1.90	0.06	2.12	0.28
GE1419445	NM_054078.1	bromodomain adjacent to zinc finger domain, 2A	Baz2a	1.89	0.18	1.80	0.20	1.57	0.14	1.10	0.17	1.48	0.14	1.34	0.17
GE35428	NM_010238.1	bromodomain containing 2	Brd2	18.12	1.70	20.20	0.44	15.86	1.24	15.76	0.43	19.83	1.24	15.86	1.50
GE40429	NM_023336.2	bromodomain containing 3	Brd3	27.62	1.32	26.55	0.88	25.72	2.02	21.84	3.25	17.62	0.93	19.66	2.62
GE1405107	NM_198094.1	bromodomain containing 4	Brd4	3.12	0.31	2.84	0.33	3.32	0.09	3.57	0.23	3.57	0.15	3.72	0.52
GE35966	NM_012047.1	bromodomain containing 7	Brd7	16.42	1.02	17.46	0.70	16.43	0.50	19.83	2.32	19.47	0.79	17.49	0.61
GE130152	NM_030147.1	bromodomain containing 8	Brd8	6.57	0.57	6.60	0.31	5.72	0.39	7.26	1.01	5.46	0.45	6.18	0.45
GE42116	NM_054054.1	bromodomain, testis-specific	Brdt	0.08	0.07	0.08	0.01	0.05	0.03	0.08	0.05	0.10	0.03	0.12	0.10
GE115742	NM_172255.1	WD repeat domain 11	Wdr11	26.43	1.06	24.43	2.22	23.32	1.22	26.55	1.62	23.90	0.77	20.92	1.16
GE35203	NM_009881.1	chromodomain protein, Y chromosome-like	Cdyl	2.03	0.09	2.16	0.16	1.77	0.18	1.93	0.25	1.93	0.25	1.53	0.03
GE125009	NM_029441.1	chromodomain protein, Y chromosome-like 2	Cdyl2	6.52	0.53	5.06	0.24	5.43	0.22	5.17	0.37	3.98	0.11	5.20	0.10
GE34848	NM_007690.1	chromodomain helicase DNA binding protein 1	Chd1	3.44	0.59	3.71	0.27	3.16	0.09	2.61	0.32	2.69	0.13	2.50	0.41
GE34130	NM_008739.1	nuclear receptor-binding SET-domain protein 1	Nsd1	0.40	0.05	0.46	0.03	0.39	0.05	0.47	0.14	0.42	0.05	0.37	0.04
GE33779	NM_009343.1	PHD finger protein 1	Phf1	2.31	0.29	2.55	0.18	2.26	0.11	2.92	0.32	2.69	0.18	2.38	0.15
GE35890	NM_011078.2	PHD finger protein 2	Phf2	1.56	0.10	1.39	0.22	1.29	0.04	1.29	0.14	1.22	0.12	1.22	0.13
GE38419	NM_026737.1	PHD finger protein 5A	Phf5a	23.43	3.09	19.57	1.04	19.07	0.80	27.10	5.44	18.97	0.77	21.38	3.36
GE39972	NM_027642.1	PHD finger protein 6	Phf6	1.36	0.03	1.47	0.18	1.33	0.10	1.46	0.13	1.21	0.09	1.16	0.04
GE38824	NM_027949.1	PHD finger protein 7	Phf7	1.44	0.34	1.23	0.04	1.05	0.10	1.72	0.13	1.37	0.11	1.38	0.27
GE1565223	NM_172705.1	PHD finger protein 13	Phf13	0.63	0.04	0.54	0.12	0.52	0.05	0.50	0.11	0.43	0.06	0.41	0.09
GE35608	NM_011919.3	inhibitor of growth family, member 1	Ing1	8.95	0.80	8.35	0.54	8.08	0.03	9.60	0.69	8.11	0.49	7.91	0.12
GE121477	NM_023626.3	inhibitor of growth family, member 3	Ing3	0.22	0.09	0.26	0.06	0.30	0.10	0.29	0.08	0.23	0.02	0.32	0.06
GE1390960	NM_133345.1	inhibitor of growth family, member 4	Ing4	10.90	0.55	9.84	1.00	9.79	0.40	11.26	1.74	9.96	0.29	9.60	1.44
GE39125	NM_025454.1	inhibitor of growth family, member 5	Ing5	0.75	0.10	0.68	0.08	0.72	0.08	0.84	0.08	0.64	0.10	0.77	0.01
GE33574	NM_009066.2	ring finger protein 1	Ring1	2.40	0.36	2.04	0.24	2.11	0.11	2.54	0.18	2.20	0.01	2.20	0.07
GE35725	NM_010774.1	methyl-CpG binding domain protein 4	Mbd4	0.73	0.04	0.74	0.03	0.68	0.03	0.48	0.08	0.49	0.08	0.40	0.09
GE36685	NM_013594.1	methyl-CpG binding domain protein 1	Mbd1	9.41	0.24	10.04	0.39	8.82	0.96	8.74	0.61	10.54	0.61	8.65	1.15
GE42644	NM_010788.1	methyl CpG binding protein 2	Mecp2	5.06	0.51	4.78	0.51	4.36	0.42	3.76	0.36	4.06	0.94	3.90	0.37
GE34909	NM_007794.1	CCCTC-binding factor	Ctcf	7.57	0.63	7.57	0.26	7.51	0.09	9.56	0.70	6.98	0.78	8.40	0.83
GE39492	NM_026115.2	histone aminotransferase 1	Hat1	9.68	1.56	8.67	0.09	7.96	0.31	10.36	0.93	7.11	0.32	8.52	1.14
GE36164	NM_011514.1	suppressor of variegation 3-9 homolog 1 (Drosophila)	Suv39h1	1.99	0.06	2.36	0.16	2.14	0.20	1.90	0.16	1.81	0.12	1.77	0.09
GE124544	NM_022724.2	suppressor of variegation 3-9 homolog 2 (Drosophila)	Suv39h2	0.31	0.10	0.39	0.06	0.38	0.03	0.32	0.09	0.37	0.03	0.33	0.09
GE137716	NM_172545.1	euchromatic histone methyltransferase 1	Ehmt1	14.53	2.31	14.27	1.07	13.80	0.88	14.89	0.56	12.21	1.73	14.84	1.97
GE34130	NM_008739.1	nuclear receptor-binding SET-domain protein 1	Nsd1	0.40	0.05	0.46	0.03	0.39	0.05	0.47	0.14	0.42	0.05	0.37	0.04
GE133641	AY555193.1	Suv4-20h2 mRNA	mRNA	18.97	1.64	15.74	1.78	17.87	0.93	16.03	2.05	11.90	0.32	13.18	0.45
GE1546389	NM_007971.1	enhancer of zeste homolog 2 (Drosophila)	Ezh2	1.98	0.48	1.71	0.47	1.52	0.17	1.38	0.12	1.07	0.26	1.36	0.19
GE34782	NM_007548.1	PR domain containing 1, with ZNF domain	Prdm1	1.59	0.14	2.17	0.19	2.09	0.06	1.68	0.28	1.15	0.08	1.44	0.11
GE123518	NM_027547.1	PR domain containing 5	Prdm5	2.45	0.11	2.32	0.20	2.35	0.07	2.92	0.25	2.15	0.25	2.76	0.19
GE40146	NM_027504.2	PR domain containing 16	Prdm16	0.69	0.04	0.64	0.03	0.55	0.02	0.80	0.03	0.75	0.05	0.66	0.08
GE112553	NM_172132.1	jumonji domain containing 2B	Jmjd2b	9.19	0.49	10.04	0.42	9.58	0.64	9.67	1.18	9.15	0.33	8.54	0.19
GE129769	NM_144787.1	jumonji domain containing 2C	Jmjd2c	4.21	0.23	4.47	0.25	4.36	0.21	4.41	0.34	4.30	0.30	4.09	0.40
GE38027	NM_021531.2	coactivator-associated arginine methyltransferase 1, mRNA	Carm1	6.34	0.68	7.43	0.53	6.53	0.33	6.76	0.52	9.13	0.88	6.52	0.84
GE1420935	NM_182999.1	ring finger protein 20	Rnf20	9.30	0.57	8.69	0.68	8.83	0.56	8.02	4.63	8.17	0.42	7.55	0.38
GE36389	NM_007788.2	casein kinase II, alpha 1 polypeptide	Csnk2a1	4.52	0.51	4.34	0.27	4.40	0.34	6.30	1.30	4.78	0.78	4.96	0.82
GE38218	NM_021420.2	serine/threonine kinase 4	Stk4	2.02	0.25	2.34	0.21	1.70	0.03	1.93	0.20	2.00	0.26	1.72	0.07

Supplementary table 6. List of hand-curated "Endocytosis genes" used for Fisher's Exact test

				(Gene expression) 2 weeks						(Gene expression) 3 weeks					
Substance	ACCN#	Description	Gene Symbol	CTL		Hom-CKO		Het-CKO		CTL		Hom-CKO		Het-CKO	
				Average	SD	Average	SD	Average	SD	Average	SD	Average	SD	Average	SD
GE34813	NM_007616.2	caveolin, caveolae protein	cav	88.96	3.49	79.47	2.14	80.54	5.28	71.31	4.80	40.27	3.48	58.45	7.89
GE38539	NM_016900.2	caveolin 2	cav2	29.43	8.04	22.46	1.51	24.51	0.64	26.14	10.30	15.64	0.84	23.32	5.25
GE41787	AB049605.1	caveolin-2 isoform	cav2	0.86	0.06	0.84	0.03	0.81	0.12	0.83	0.19	0.64	0.06	0.65	0.19
GE34812	NM_007617.2	caveolin 3	cav3	41.06	3.68	36.45	6.30	35.67	2.72	48.52	3.26	43.16	2.87	44.86	3.63
GE41276	NM_026514.1	CDC42 effector protein (Rho GTPase binding) 3	cdc42ep3	8.67	1.27	7.50	0.48	7.65	0.19	10.54	1.60	9.47	0.70	8.51	0.96
GE37649	NM_020006.1	CDC42 effector protein (Rho GTPase binding) 4	cdc42ep4	1.77	0.16	1.68	0.09	1.52	0.17	1.75	0.17	1.64	0.14	1.43	0.16
GE33178	NM_172395.1	small protein effector 1 of Cdc42	spec1	6.93	0.02	7.78	0.71	6.42	0.25	7.35	0.67	6.88	0.72	5.53	0.94
GE37762	NM_020260.1	Cdc42 GTPase-activating protein	cdgap	4.78	0.21	5.41	0.49	5.18	0.25	5.07	0.23	4.85	0.81	4.58	0.66
GE39014	NM_152801.1	Rac/Cdc42 guanine nucleotide exchange factor (GEF) 6	arhgef6	0.74	0.08	0.66	0.05	0.63	0.04	0.70	0.15	0.69	0.02	0.62	0.09
GE123266	NM_027219.1	CDC42 effector protein (Rho GTPase binding) 1	cdc42ep1	2.53	0.82	3.19	0.20	2.67	0.16	2.68	0.42	2.83	0.44	2.23	0.32
GE102285	NM_027219.1	CDC42 effector protein (Rho GTPase binding) 1	cdc42ep1	14.38	0.90	13.10	FALSE	12.26	0.91	12.49	0.60	13.51	1.94	11.48	1.02
GE1557652	AA798249.1	vy05e09r1 Stratagene mouse macrophage (#937306) cDNA clone IMAGE:1294600 5' similar to gb:U37720 Mus musculus CDC42 mRNA, complete cds (MOUSE);.	EST	2.33	0.18	2.83	0.40	2.79	0.19	2.66	0.15	3.11	0.31	2.71	0.22
GE34757	NM_007476.2	ADP-ribosylation factor 1	arf1	78.98	2.11	72.41	1.60	69.46	4.49	92.78	4.91	76.59	8.86	83.38	6.82
GE38672	NM_029702.2	ADP-ribosylation factor related protein 1	arfrp1	11.41	0.71	10.89	0.40	9.73	0.12	14.57	0.79	12.04	1.00	11.05	0.73
GE34759	D87903.1	ADP-ribosylation factor 6	arf6	13.49	0.82	11.63	0.77	9.90	0.18	12.26	0.73	10.28	0.76	9.23	0.92
GE132386	NM_145760.2	ADP-ribosylation factor GTPase activating protein 1	arfgap1	3.86	1.13	3.88	0.25	3.47	0.15	4.00	0.52	3.45	0.15	3.02	0.73
GE137813	NM_172595.1	ADP-ribosylation factor related protein 2	arfrp2	8.82	1.14	8.30	0.03	8.10	0.11	7.84	0.82	6.88	0.77	6.27	0.21
GE33430	NM_007477.2	ADP-ribosylation factor 2	arf2	1.43	0.27	1.62	0.23	1.53	0.19	1.50	0.57	1.42	0.04	1.28	0.07
GE34758	NM_007479.2	ADP-ribosylation factor 4	arf4	43.19	10.45	43.13	1.62	36.25	2.02	43.96	3.47	42.62	1.37	36.97	1.46
GE42460	NM_007480.1	ADP-ribosylation factor 5	arf5	20.03	2.72	21.81	1.72	17.46	0.62	21.63	2.04	21.37	0.24	16.42	1.76
GE39327	NM_029802.2	ADP-ribosylation factor interacting protein 2	arfip2	8.53	1.42	8.38	0.29	7.27	0.57	7.10	0.25	7.28	0.50	5.92	0.30
GE121372	NM_025445.2	ADP-ribosylation factor GTPase activating protein 3	arfgap3	4.21	0.30	4.16	0.17	3.47	0.17	4.31	0.63	4.85	0.44	3.77	0.11
GE34304	NM_008986.1	polymerase I and transcript release factor	ptrf	4.92	0.76	4.95	0.10	4.77	0.11	4.80	0.84	3.45	0.27	3.51	0.40
GE34407	NM_008028.1	flotillin 2	flot2	3.24	0.58	2.96	0.25	3.13	0.20	3.76	0.68	2.91	0.07	3.51	1.12
GE34406	NM_008027.1	flotillin 1	flot1	16.30	1.81	15.66	0.32	15.43	0.56	27.43	7.06	18.95	1.89	21.16	0.40
GE41457	NM_030113.1	Rho GTPase activating protein 10	arhgap10	1.81	0.22	2.02	0.25	1.72	0.09	2.32	0.23	2.30	0.03	2.08	0.49
GE137149	NM_153800.2	Rho GTPase activating protein 22	arhgap22	0.79	0.12	0.57	0.03	0.52	0.02	0.90	0.13	0.92	0.14	0.86	0.10
GE1451690	NM_029277.1	Rho GTPase activating protein 12	arhgap12	7.62	0.48	6.63	0.29	6.49	0.26	7.23	1.14	7.56	0.49	6.14	0.18
GE35087	NM_009706.1	Rho GTPase activating protein 5	arhgap5	2.14	0.42	2.14	0.18	2.29	0.07	1.91	0.38	2.49	0.69	2.23	0.27
GE39887	NM_176837.1	Rho GTPase activating protein 18	arhgap18	6.90	0.78	6.07	0.80	5.92	0.24	5.09	0.28	3.39	0.43	3.93	0.24
GE35088	NM_009707.1	Rho GTPase activating protein 6	arhgap6	0.22	0.14	0.25	0.06	0.23	0.02	0.28	0.03	0.19	0.04	0.21	0.03
GE1433043	NM_175476.2	Rho GTPase activating protein 25	arhgap25	0.64	0.05	0.59	0.07	0.64	0.05	0.71	0.06	0.56	0.03	0.57	0.03
GE133577	NM_029270.1	Rho GTPase activating protein 24	arhgap24	11.80	0.21	9.99	0.49	9.82	0.41	12.72	1.65	10.19	0.28	9.90	2.26
GE128907	NM_138630.1	Rho GTPase activating protein 4	arhgap4	0.41	0.16	0.46	0.06	0.45	0.04	0.61	0.26	0.52	0.04	0.49	0.12
GE133429	NM_146124.2	Rho GTPase activating protein 1	arhgap1	5.02	0.06	5.36	0.41	4.51	0.21	5.58	0.58	4.95	0.55	5.00	0.06
GE41604	NM_144529.1	Rho GTPase activating protein 17	arhgap17	2.88	0.36	2.53	0.39	2.25	0.01	2.30	0.16	2.18	0.17	2.00	0.12
GE42652	NM_011035.1	p21 (CDKN1A)-activated kinase 1	pak1	0.06	0.04	0.07	0.05	0.06	0.11	0.12	0.14	0.18	0.02	0.10	0.07
GE122508	NM_026550.1	PAK1 interacting protein 1	pak1ip1	23.10	3.46	19.35	1.31	18.21	0.68	27.58	4.08	26.33	2.32	23.55	1.30
GE33401	NM_007380.1	abl-interactor 1	abi1	18.93	3.26	18.56	1.39	16.06	0.11	16.35	2.92	20.78	1.08	15.27	1.27
GE42708	NM_013502.1	C-terminal binding protein 1	ctbp1	38.29	2.75	35.87	1.83	34.48	1.71	43.26	2.50	33.43	1.60	37.64	4.89
GE37606	NM_019727.1	sorting nexin 1	snx1	3.25	0.38	2.94	0.16	2.73	0.10	4.61	1.16	2.79	0.23	3.14	0.38
GE102589	NM_026912.1	sorting nexin 15	snx15	33.41	0.48	30.08	2.31	28.30	1.51	37.74	6.55	26.46	2.42	29.56	3.16
GE37206	NM_017472.2	sorting nexin 3	snx3	30.59	2.80	31.71	1.50	28.83	1.09	40.89	3.41	34.64	3.91	34.31	2.10
GE124661	NM_028965.2	sorting nexin 11	snx11	3.07	0.80	3.32	0.06	3.19	0.11	4.00	0.70	3.45	0.49	2.96	0.32
GE121081	NM_024225.2	sorting nexin 5	snx5	13.18	1.92	12.93	1.19	10.89	1.07	14.73	1.58	13.55	0.77	11.10	1.19
GE137075	NM_153680.1	sorting nexin 17	snx17	13.47	0.40	13.58	0.50	14.38	0.16	15.87	0.61	15.17	1.28	13.56	0.36
GE39839	NM_028874.1	sorting nexin 19	snx19	1.73	0.25	1.80	0.14	1.65	0.14	1.95	0.04	1.90	0.17	1.93	0.44
GE1503708	NM_025664.2	sorting nexin 9	snx9	0.18	0.03	0.12	0.01	0.16	0.04	0.09	0.05	0.09	0.07	0.14	0.03
GE39951	NM_029068.2	sorting nexin 16	snx16	3.19	0.69	3.08	0.31	2.73	0.20	3.51	0.24	3.55	0.57	3.47	0.24
GE123010	NM_026998.2	sorting nexin 6	snx6	10.70	1.61	10.61	0.08	9.04	0.50	9.73	1.12	9.98	0.44	8.49	0.81
GE138366	NM_172926.1	sorting nexin 14	snx14	4.00	0.50	4.66	0.12	4.52	0.19	4.26	0.47	4.41	0.43	4.06	0.39
GE123850	NM_028035.2	sorting nexin 10	snx10	3.71	1.24	3.52	0.26	2.78	0.16	3.84	0.41	4.01	0.21	3.24	0.53
GE124937	NM_029394.2	sorting nexing 24	snx24	9.69	0.37	8.76	0.41	8.46	0.39	9.09	0.90	9.52	0.36	8.99	0.99

Supplementary table 6. Cont'd….

Substance	ACCN#	Description	Gene Symbol	(Gene expression) 2 weeks						(Gene expression) 3 weeks					
				CTL		Hom-CKO		Het-CKO		CTL		Hom-CKO		Het-CKO	
				**Average**	**SD**	**Average**	**SD**	**Average**	**SD**	**Average**	**SD**	**Average**	**SD**	**Average**	**SD**
GE102398	NM_028035.2	sorting nexin 10	snx10	3.72	1.19	3.46	0.39	2.85	0.15	3.85	0.82	4.06	0.22	3.18	0.78
GE37338	NM_018875.1	sorting nexin 12	snx12	7.40	0.85	8.07	0.31	6.36	0.16	9.45	1.84	11.35	1.06	7.17	0.38
GE137374	NM_172277.1	sorting nexin 8	snx8	2.07	0.32	2.65	0.03	2.25	0.15	2.75	0.27	3.70	0.16	2.37	0.07
GE1409382	NM_178252.2	sorting nexin 26	snx26	0.79	0.14	1.01	0.09	0.66	0.07	0.55	0.12	0.80	0.11	0.40	0.02
GE39161	NM_080557.1	sorting nexin 4	snx4	0.17	0.05	0.19	0.04	0.28	0.07	0.12	0.15	0.22	0.04	0.09	0.05
GE34962	NM_007871.1	dynamin 2	dnm2	6.91	1.48	7.00	0.51	6.85	0.18	8.02	1.89	7.00	0.81	6.74	1.10
GE39451	NM_028029.1	dynamin binding protein	dnmbp	1.82	0.14	1.77	0.11	1.62	0.21	2.07	0.22	1.86	0.04	1.81	0.10
GE42423	NM_152816.1	dynamin 1-like	dnm1l	9.19	1.34	9.79	1.00	10.65	3.22	11.25	1.80	10.91	1.19	11.21	3.03
GE33022	NM_010065.1	dynamin	dnm	1.73	0.12	1.84	0.11	1.88	0.06	1.60	0.31	2.22	0.10	1.70	0.10
GE34227	NM_008876.1	phospholipase D2	pld2	1.77	0.13	1.73	0.13	1.73	0.21	1.57	0.13	1.36	0.15	1.58	0.12
GE33206	NM_008875.3	phospholipase D1	pld1	1.96	0.18	1.98	0.09	1.98	0.13	2.40	0.10	2.23	0.12	2.32	0.28
GE36515	NM_011116.1	phospholipase D3	pld3	8.21	0.44	8.17	0.21	7.64	0.69	9.31	1.62	8.79	0.78	7.16	1.01
GE34507	NM_008156.1	glycosylphosphatidylinositol specific phospholipase D1	gpld1	0.36	0.03	0.43	0.01	0.29	0.05	0.37	0.11	0.67	0.10	0.41	0.08
GE34235	NM_008888.1	paired-like homeobox 2b	phox2b	12.95	1.07	11.08	1.45	11.94	0.63	15.43	3.63	12.46	0.60	13.17	1.44
GE42549	NM_008887.1	paired-like homeobox 2a	phox2a	1.34	0.09	1.21	0.06	1.28	0.05	1.49	0.01	1.24	0.10	1.23	0.10
GE37950	NM_021429.1	HS1 binding protein 3	hs1bp3	1.37	0.24	1.64	0.14	1.44	0.03	1.80	0.09	2.29	0.13	1.52	0.31
GE34081	NM_008677.1	neutrophil cytosolic factor 4	ncf4	0.73	0.22	0.63	0.02	0.63	0.09	1.33	0.19	1.39	0.33	0.89	0.23
GE116569	NM_010877.2	neutrophil cytosolic factor 2	ncf2	0.16	0.03	0.17	0.07	0.21	0.05	0.28	0.16	0.14	0.10	0.17	0.07
GE32989	NM_010876.2	neutrophil cytosolic factor 1	ncf1	0.55	0.11	0.47	0.04	0.44	0.01	0.90	0.12	0.68	0.03	0.65	0.16
GE38217	NM_022656.1	nischarin	nisch	24.29	4.63	25.33	0.76	25.21	1.14	21.08	3.32	20.78	2.47	20.35	3.11
GE35892	NM_011083.1	phosphatidylinositol 3-kinase, C2 domain containing, alpha polypeptide	pik3c2a	1.22	0.19	1.36	0.15	1.28	0.10	1.08	0.17	0.98	0.05	1.04	0.03
GE35893	NM_011084.1	phosphatidylinositol 3-kinase, C2 domain containing, gamma polypeptide, transcript variant 2	pik3c2g	13.10	1.76	12.28	0.84	12.86	0.77	14.47	1.04	12.90	0.67	13.07	1.52
GE131376	NM_145458.1	PX domain containing serine/threonine kinase	pxk	2.82	0.30	2.97	0.13	2.83	0.28	3.55	0.09	3.41	0.32	3.12	0.16
GE42284	NM_130796.1	sorting nexin associated golgi protein 1	snag1	5.90	1.11	5.57	0.42	4.82	0.66	3.62	0.54	3.80	0.23	3.08	0.15
GE37023	NM_016760.1	clathrin, light polypeptide (Lca)	clta	0.07	0.05	0.16	0.06	0.15	0.05	0.12	0.09	0.18	0.09	0.05	0.05
GE39399	NM_028870.1	clathrin, light polypeptide (Lcb)	cltb	23.26	0.74	25.04	1.21	21.63	1.32	29.52	2.92	32.08	0.41	22.56	0.31
GE39838	NM_028715.2	FCH domain only 1	fcho1	0.77	0.03	1.10	0.28	1.31	0.17	0.87	0.10	0.91	0.06	0.87	0.05
GE38622	NM_027915.1	adaptor-related protein complex 2, beta 1 subunit	ap2b1	2.50	0.24	2.95	0.19	3.06	0.21	2.52	0.24	2.90	0.20	2.57	0.06
GE34348	NM_007943.1	epidermal growth factor receptor pathway substrate 15	eps15	3.29	0.38	3.55	0.40	2.97	0.20	3.00	0.50	3.57	0.08	3.01	0.26
GE36776	NM_013669.1	synaptosomal-associated protein 91	snap91	3.89	0.98	5.63	0.42	4.03	0.23	3.69	0.89	5.16	0.43	2.69	0.52
GE133724	NM_146194.2	phosphatidylinositol binding clathrin assembly protein	picalm	36.99	1.54	33.56	2.68	31.20	2.47	30.54	3.32	32.81	3.84	25.60	1.22
GE33251	NM_010148.1	epsin 2	epn2	3.77	0.64	3.62	0.21	3.69	0.12	3.97	0.13	3.51	0.15	3.16	0.38
GE39369	NM_027984.1	epsin 3	epn3	5.41	0.61	4.71	0.03	6.10	0.26	11.23	4.15	9.13	0.69	8.70	2.40
GE42599	NM_009668.1	bridging integrator 1	bin1	2.65	0.13	2.69	0.21	2.60	0.16	3.37	0.14	2.41	0.36	2.64	0.33
GE39696	NM_198412.1	DnaJ (Hsp40) homolog, subfamily C, member 6	dnajc6	0.06	0.02	0.10	0.04	0.04	0.03	0.09	0.03	0.09	0.06	0.09	0.05
GE40365	NM_023118.1	disabled homolog 2 (Drosophila)	dab2	10.10	3.38	8.59	0.08	7.54	0.01	13.11	2.29	8.68	0.45	11.19	2.44
GE35834	NM_010949.1	numb gene homolog (Drosophila)	numb	6.17	0.86	6.62	0.48	6.26	0.41	6.56	1.39	5.03	0.42	5.53	0.86
GE131239	NM_145429.1	arrestin, beta 2	arrb2	1.96	0.47	1.72	0.18	1.59	0.11	2.58	0.51	1.87	0.25	2.17	0.49
GE1431218	NM_177231.1	arrestin, beta 1, transcript variant a	arrb1	1.13	0.10	1.13	0.09	1.15	0.03	1.24	0.05	1.05	0.15	1.14	0.04
GE1414816	NM_178220.2	arrestin, beta 1, transcript variant b	arrb1	0.90	0.06	0.90	0.02	0.94	0.15	0.74	0.05	0.85	0.09	0.82	0.09
GE119398	NM_011523.1	synaptojanin 2	synj2	0.54	0.17	0.60	0.07	0.63	0.04	0.78	0.13	0.92	0.10	0.75	0.07
GE117766	NM_007890.1	dual-specificity tyrosine-(Y)-phosphorylation regulated kinase 1a	dyrk1a	4.09	0.42	4.60	0.29	4.23	0.15	4.19	0.51	6.82	0.23	3.82	0.40
GE132915	NM_146001.1	huntingtin interacting protein 1	hip1	3.00	0.18	3.29	0.36	2.97	0.17	2.84	0.30	3.01	0.14	2.82	0.10
GE1507560	AI465422.1	mu84b05x1 Stratagene mouse melanoma (#937312) cDNA clone IMAGE:652209 3'.	EST	0.79	0.03	0.93	0.11	0.82	0.07	0.51	0.16	0.80	0.13	0.74	0.03
GE36768	NM_013664.1	SH3-domain GRB2-like 1	sh3gl1	6.69	0.19	6.70	0.28	5.77	0.52	6.83	0.06	6.27	0.52	5.37	0.62
GE37424	NM_019535.2	SH3-domain GRB2-like 2	sh3gl2	0.07	0.09	0.08	0.04	0.06	0.03	0.10	0.06	0.08	0.01	0.22	0.11
GE127349	NM_133816.1	SH3-domain binding protein 4	sh3bp4	18.20	1.39	18.53	1.88	18.08	1.55	20.31	1.49	18.65	0.62	17.84	1.15

Supplementary table 7. List of hand-curated "Autophagy genes" used for Fisher's Exact test

				(Gene expression) 2 weeks						(Gene expression) 3 weeks					
Substance	ACCN#	Description	Gene Symbol	CTL		Hom-CKO		Het-CKO		CTL		Hom-CKO		Het-CKO	
				Average	SD	Average	SD	Average	SD	Average	SD	Average	SD	Average	SD
GE33867	NM_009469.3	Unc-51 like kinase 1 (C elegans)	Ulk1	3.74	0.67	4.78	0.38	3.69	0.29	3.64	0.09	4.91	0.42	3.45	0.17
GE37442	NM_019584.2	beclin 1 (coiled-coil, myosin-like BCL2-interacting protein)	Becn1	11.66	1.65	11.69	0.59	10.30	0.53	12.58	0.69	11.27	1.24	9.89	0.55
GE42785	NM_019749.3	gamma-aminobutyric acid receptor associated protein	Gabarap	39.03	8.00	30.62	0.95	29.74	1.16	50.85	8.05	37.82	2.43	39.00	1.65
GE37839	NM_020590.3	gamma-aminobutyric acid (GABA(A)) receptor-associated protein-like 1	Gabarapl1	0.54	0.09	0.59	0.05	0.55	0.09	0.70	0.13	0.72	0.09	0.50	0.06
GE41343	NM_026693.2	GABA(A) receptor-associated protein like 2	Gabarapl2	38.39	9.00	32.34	1.02	29.16	1.13	56.61	8.83	40.47	1.01	43.11	8.80
GE35850	NM_011018.1	sequestosome 1	Sqstm1	65.79	0.35	67.07	3.00	55.29	0.96	87.52	8.33	88.37	1.39	64.83	3.01
GE33996	NM_008562.2	myeloid cell leukemia sequence 1	Mcl1	24.84	2.36	21.99	3.29	23.81	0.91	29.28	5.15	24.62	1.53	26.17	2.75
GE32991	NM_009713.1	arylsulfatase A	Arsa	3.40	0.46	3.38	0.09	3.30	0.23	3.92	0.97	2.73	0.06	2.82	0.08
GE37871	NM_021281.1	cathepsin S	Ctss	11.25	4.18	9.19	0.43	8.38	0.73	17.88	3.95	23.13	5.42	13.37	4.55
GE42492	NM_007956.2	estrogen receptor 1 (alpha)	Esr1	0.12	0.02	0.09	0.07	0.11	0.06	0.15	0.04	0.05	0.09	0.13	0.02
GE34440	NM_008064.2	glucosidase, alpha, acid	Gaa	4.45	2.06	4.84	0.02	5.20	0.24	8.34	2.98	6.69	0.34	6.28	1.95
GE34567	NM_008244.2	HGF-regulated tyrosine kinase substrate	Hgs	2.51	0.75	3.12	0.14	2.96	0.25	3.16	0.53	2.85	0.10	2.29	0.41
GE1413075	NM_181414.3	phosphoinositide-3-kinase, class 3	Pik3c3	4.51	0.23	3.98	0.19	3.98	0.25	4.57	0.42	3.99	0.01	3.69	0.23
GE39293	AK008703.1	Phosphatidylinositol 3 kinase, regulatory subunit, polypeptide 4, p150	Pik3r4	6.74	0.73	4.23	0.49	5.78	0.43	8.76	0.83	3.75	0.16	6.86	0.28
GE36815	NM_013881.3	Unc-51 like kinase 2 (C elegans)	Ulk2	9.53	0.70	9.71	0.73	8.96	0.24	7.52	1.12	7.86	0.81	7.55	0.60
GE1496640	NM_178635.2	UV radiation resistance associated gene	Uvrag1	12.96	1.00	10.44	0.47	12.01	1.07	12.95	0.83	11.18	0.53	11.27	1.64
GE1396310	NM_175029.2	APG4 (ATG4) autophagy-related homolog C (S cerevisiae)	Apg4c	0.49	0.03	0.56	0.06	0.50	0.03	0.32	0.11	0.39	0.05	0.45	0.07
GE104096	NM_174874.1	APG4 (ATG4) autophagy-related homolog B (S cerevisiae)	Apg4b	6.79	0.51	7.31	0.36	5.94	0.40	7.42	1.39	9.71	0.25	6.54	0.80
GE37442	NM_019584.2	beclin 1 (coiled-coil, myosin-like BCL2-interacting protein)	Becn1	11.66	1.65	11.69	0.59	10.30	0.53	12.58	0.69	11.27	1.24	9.89	0.55
GE121691	NM_025735.1	microtubule-associated protein 1 light chain 3 alpha	Map1lc3a	154.98	25.90	115.61	6.01	131.39	3.05	217.00	21.81	135.63	11.35	179.85	21.84
GE41120	NM_026160.1	microtubule-associated protein 1 light chain 3 beta	Map1lc3b	58.89	1.43	54.57	6.36	50.64	3.74	70.76	1.80	49.26	3.08	53.54	1.71
GE41247	NM_026446.2	regulator of G-protein signaling 19	Rgs19	2.37	0.32	2.50	0.14	2.24	0.03	2.87	0.64	2.57	0.14	2.35	0.56
GE39135	NM_009000.2	RAB24, member RAS oncogene family	Rab24	8.35	1.04	8.05	1.13	7.96	0.02	11.41	1.46	8.89	1.28	9.26	0.66
GE35054	NM_009652.1	thymoma viral proto-oncogene 1	Akt1	15.11	0.71	15.15	0.63	12.93	0.49	16.44	1.49	12.86	1.77	13.97	0.93
GE42459	NM_007471.2	amyloid beta (A4) precursor protein	App	12.18	3.96	13.89	1.23	13.44	1.35	16.70	4.18	13.02	1.14	10.35	1.47
GE33446	NM_007522.1	Bcl-associated death promoter	Bad	1.71	0.29	1.61	0.11	1.35	0.13	1.65	0.20	1.74	0.11	1.70	0.36
GE33447	NM_007523.1	BCL2-antagonist/killer 1	Bak1	2.50	0.08	3.01	0.28	2.76	0.21	3.00	0.44	2.84	0.18	2.29	0.13
GE34777	NM_007527.2	Bcl2-associated X protein	Bax	9.61	0.60	12.40	1.35	8.31	0.21	13.73	2.05	21.01	0.91	11.14	1.97
GE35110	NM_009741.2	B-cell leukemia/lymphoma 2	Bcl2	4.07	0.77	5.24	0.96	3.42	0.23	2.95	0.13	4.75	0.47	2.75	0.06
GE1438110	NM_029410.1	BCL2-like 12 (proline rich)	Bcl2l12	6.86	0.75	5.50	0.16	5.61	0.15	6.89	0.94	4.17	0.19	5.45	0.87
GE35123	NM_009760.2	BCL2/adenovirus E1B 19kDa-interacting protein 1, NIP3	Bnip3	37.57	8.86	30.90	0.79	31.06	3.23	51.54	1.29	47.15	2.00	43.99	0.68
GE35157	NM_009810.1	caspase 3, apoptosis related cysteine protease	Casp3	2.25	0.68	2.18	0.12	1.90	0.13	1.60	0.41	1.63	0.16	1.48	0.30
GE35201	NM_009877.1	cyclin-dependent kinase inhibitor 2A	Cdkn2a	0.23	0.10	0.30	0.05	0.37	0.05	0.15	0.08	0.24	0.05	0.25	0.08
GE35226	NM_009907.2	ceroid lipofuscinosis, neuronal 3, juvenile (Batten, Spielmeyer-Vogt disease)	Cln3	3.44	0.03	3.29	0.28	3.14	0.28	4.01	0.17	3.57	0.43	3.07	0.23
GE34911	NM_007798.1	cathepsin B	Ctsb	8.52	0.57	8.07	0.65	7.83	0.06	10.62	2.37	10.41	0.96	9.40	1.17
GE1453040	NM_009911.2	chemokine (C-X-C motif) receptor 4	Cxcr4	8.64	2.67	7.11	0.35	7.66	1.19	6.71	1.78	3.88	0.69	5.09	0.38
GE39382	NM_029653.1	death associated protein kinase 1	Dapk1	0.80	0.02	0.91	0.07	0.74	0.09	1.00	0.21	0.91	0.03	0.79	0.08
GE36337	NM_010121.1	eukaryotic translation initiation factor 2 alpha kinase 3	Eif2ak3	6.00	0.63	5.63	0.23	5.07	0.45	4.45	0.40	4.34	0.17	3.76	0.31
GE35383	NM_010175.2	Fas (TNFRSF6)-associated via death domain	Fadd	5.80	0.58	5.86	0.23	5.51	0.28	6.51	0.87	7.98	0.76	5.27	0.76
GE42450	NM_008337.1	interferon gamma	Ifng	0.08	0.03	0.05	0.03	0.05	0.07	0.13	0.10	0.05	0.04	0.19	0.08
GE35590	NM_010512.2	insulin-like growth factor 1	Igf1	3.52	0.69	4.79	0.60	2.75	0.14	3.39	0.11	3.49	0.55	2.68	0.56
GE36985	NM_016700.2	mitogen activated protein kinase 8	Mapk8	2.67	0.46	2.36	0.11	2.46	0.13	2.05	0.47	1.78	0.04	2.14	0.25
GE34091	NM_008689.1	nuclear factor of kappa light chain gene enhancer in B-cells 1, p105	Nfkb1	11.19	0.38	10.29	1.04	9.42	1.00	10.60	0.80	8.59	0.02	8.82	0.54
GE37748	NM_020272.1	phosphoinositide-3-kinase, catalytic, gamma polypeptide	Pik3cg	1.56	0.19	1.53	0.09	1.38	0.10	1.62	0.15	1.74	0.14	1.55	0.34
GE42554	NM_008960.1	phosphatase and tensin homolog	Pten	8.17	0.70	8.67	0.68	7.33	0.39	8.31	0.73	11.07	1.49	8.48	0.90
GE33695	NM_009221.1	synuclein, alpha	Snca	8.94	2.62	8.10	1.48	8.22	1.02	20.04	3.16	11.77	0.54	13.97	2.27
GE36197	NM_011577.1	transforming growth factor, beta 1	Tgfb1	4.51	0.15	5.55	0.39	4.13	0.27	3.77	0.67	5.11	0.46	3.56	0.43

Supplementary table 7. Cont'd….

Substance	ACCN#	Description	Gene Symbol	(Gene expression) 2 weeks						(Gene expression) 3 weeks					
				CTL		Hom-CKO		Het-CKO		CTL		Hom-CKO		Het-CKO	
				Average	SD	Average	SD	Average	SD	Average	SD	Average	SD	Average	SD
GE33805	NM_009373.2	transglutaminase 2, C polypeptide	Tgm2	4.49	1.01	4.45	0.32	5.11	0.04	6.89	1.15	7.71	0.55	5.92	1.39
GE36806	NM_013693.1	tumor necrosis factor	Tnf	0.16	0.07	0.33	0.04	0.27	0.07	0.29	0.05	0.33	0.16	0.29	0.01
GE33843	NM_009425.1	tumor necrosis factor (ligand) superfamily, member 10	Tnfsf10	3.06	0.48	2.94	0.06	2.90	0.21	2.83	0.24	2.60	0.14	2.34	0.26
GE36238	NM_011640.1	transformation related protein 53	Trp53	9.20	0.97	10.17	0.49	8.70	0.43	10.84	2.13	7.54	1.53	7.63	0.56
GE1432611	NM_011642.1	transformation related protein 73	Trp73	0.06	0.06	0.09	0.03	0.03	0.04	0.10	0.06	0.15	0.09	0.09	0.06
GE33542	NM_009029.1	retinoblastoma 1	Rb1	3.70	0.34	3.66	0.08	3.54	0.24	3.40	0.33	3.70	0.73	3.34	0.43
GE118948	NM_010504.1	interferon alpha family, gene 4, mRNA	Ifna4	0.61	0.21	0.62	0.04	0.77	0.12	0.66	0.10	0.67	0.06	0.57	0.11
GE35667	NM_010685.2	lysosomal membrane glycoprotein 2	Lamp2	19.88	3.35	15.40	0.19	15.56	1.17	17.67	1.71	18.20	0.88	13.70	1.19
GE38101	NM_021897.1	transformation related protein 53 inducible nuclear protein 1	Trp53inp1	10.84	0.76	10.66	0.81	10.45	0.19	11.23	2.09	10.97	0.40	9.63	0.35
GE40675	NM_025307.1	nuclear receptor binding factor 2	Nrbf2	1.53	0.22	1.67	0.18	1.38	0.09	1.62	0.23	2.16	0.06	1.55	0.25
GE38728	NM_028162.2	TBC1 domain family, member 5	Tbc1d5	1.14	0.18	1.17	0.10	1.09	0.07	1.05	0.12	1.37	0.05	1.00	0.08
GE41811	NM_009826.3	RB1-inducible coiled-coil 1	Rb1cc1	11.17	0.44	9.37	0.55	9.98	0.07	9.40	2.44	9.93	0.23	9.31	0.40
GE34298	NM_008979.1	protein tyrosine phosphatase, non-receptor type 8	Ptpn8	0.09	0.07	0.07	0.03	0.08	0.03	0.10	0.10	0.10	0.02	0.07	0.03
GE37425	NM_019464.1	SH3-domain GRB2-like B1 (endophilin)	Sh3glb1	6.76	0.62	7.66	0.54	7.21	0.32	9.29	2.42	7.21	0.13	7.08	1.26
GE37442	NM_019584.2	beclin 1 (coiled-coil, myosin-like BCL2-interacting protein)	Becn1	11.66	1.65	11.69	0.59	10.30	0.53	12.58	0.69	11.27	1.24	9.89	0.55
GE35600	NM_010556.2	interleukin 3	Il3	2.64	0.16	3.04	0.18	2.99	0.15	3.02	0.30	3.19	0.31	3.27	0.37
GE34809	NM_007601.1	calpain 3	Capn3	0.84	0.12	0.58	0.15	0.88	0.10	1.28	0.31	0.63	0.15	1.24	0.28
GE35147	NM_009796.1	calpain 7	Capn7	20.44	1.14	17.26	0.55	18.39	0.83	19.92	2.72	14.03	0.83	18.11	0.36
GE34810	NM_007602.2	calpain 5	Capn5	0.44	0.10	0.52	0.08	0.56	0.03	0.58	0.19	0.46	0.03	0.54	0.08
GE33464	NM_007600.2	calpain 1	Capn1	1.63	0.29	1.68	0.26	1.63	0.13	1.76	0.23	1.47	0.09	1.67	0.09
GE39288	NM_023709.2	calpain 9 (nCL-4)	Capn9	13.63	0.81	13.24	1.10	13.55	0.27	13.48	0.88	13.78	0.78	11.77	0.22
GE33465	NM_007603.2	calpain 6	Capn6	0.92	0.30	0.72	0.11	0.64	0.12	0.80	0.12	0.82	0.07	0.68	0.02
GE126639	NM_130890.1	calpain 8	Capn8	0.42	0.03	0.50	0.06	0.50	0.08	0.45	0.08	0.48	0.02	0.47	0.07
GE42689	NM_011796.1	calpain 10	Capn10	4.25	0.64	4.02	0.03	4.04	0.41	4.35	0.48	4.64	0.37	3.91	0.20
GE35145	NM_009794.1	calpain 2	Capn2	8.73	1.45	8.98	0.98	7.94	0.68	8.38	1.79	9.27	0.56	8.16	1.13
GE102904	NM_008100.2	glucagon	Gcg	0.09	0.08	0.08	0.07	0.10	0.01	0.14	0.03	0.07	0.06	0.04	0.02
GE33863	NM_009462.1	ubiquitin specific protease 10	Usp10	7.95	1.44	9.50	0.78	8.07	0.73	9.38	1.44	9.92	0.77	7.78	1.40
GE39929	NM_181848.3	optineurin	Optn	0.31	0.12	0.43	0.03	0.35	0.08	0.46	0.02	0.49	0.06	0.38	0.15
GE39775	NM_024448.1	RAB12, member RAS oncogene family	Rab12	24.44	1.79	18.95	0.63	21.33	0.40	31.48	7.28	21.25	2.90	22.54	1.00
GE42450	NM_008337.1	interferon gamma	Ifng	0.08	0.03	0.05	0.03	0.05	0.07	0.13	0.10	0.05	0.04	0.19	0.08
GE1487545	AK015883.1	ubiquitin-conjugating enzyme E2D N-terminal like 2	Ube2dnl2	0.08	0.04	0.04	0.05	0.07	0.04	0.13	0.04	0.06	0.06	0.11	0.02
GE127231	AK003078.1	ubiquitin-conjugating enzyme E2	Ube2s	20.96	1.55	18.33	1.35	16.92	1.49	20.94	0.65	18.03	2.49	17.90	1.48
GE39110	NM_028835.1	autophagy 7-like (S cerevisiae)	Apg7l	3.80	0.28	3.36	0.08	3.59	0.53	4.11	0.42	3.45	0.26	3.45	0.14
GE41146	NM_026217.1	autophagy 12-like (S cerevisiae)	Apg12l	10.92	1.74	10.69	0.96	9.49	0.16	13.10	0.11	11.06	0.54	10.13	0.58
GE41996	NM_053069.2	autophagy 5-like (S cerevisiae)	Apg5l	11.00	3.48	9.04	0.68	8.80	0.47	11.02	0.86	9.61	0.11	10.08	1.02
GE38675	NM_029846.2	APG16 autophagy 16-like (S cerevisiae), transcript variant b	Apg16l	7.43	0.28	6.87	0.34	6.59	0.46	7.51	0.50	7.16	0.17	7.23	0.57
GE1396310	NM_175029.2	APG4 (ATG4) autophagy-related homolog C (S cerevisiae)	Apg4c	0.49	0.03	0.56	0.06	0.50	0.03	0.32	0.11	0.39	0.05	0.45	0.07
GE104096	NM_174874.1	APG4 (ATG4) autophagy-related homolog B (S cerevisiae)	Apg4b	6.79	0.51	7.31	0.36	5.94	0.40	7.42	1.39	9.71	0.25	6.54	0.80
GE34080	NM_008676.1	neighbor of Brca1 gene 1	Nbr1	13.99	1.32	13.60	2.17	13.30	1.38	13.19	1.72	11.27	0.68	11.90	0.74
GE34317	NM_009005.1	RAB7, member RAS oncogene family	Rab7	55.45	3.18	49.76	3.37	48.73	2.79	77.79	13.04	55.06	7.09	60.80	3.41
GE35949	NM_011183.1	presenilin 2	Psen2	1.94	0.11	2.09	0.14	1.76	0.09	2.63	0.55	2.18	0.22	1.77	0.18
GE35271	NM_009983.2	cathepsin D	Ctsd	68.22	2.88	60.24	2.03	59.99	2.15	104.40	9.92	93.19	9.37	72.94	6.78
GE35270	NM_009982.2	cathepsin C	Ctsc	10.01	2.25	6.57	0.48	7.88	0.45	17.89	3.60	10.27	1.67	14.05	3.49
GE36391	NM_007801.1	cathepsin H	Ctsh	9.09	3.25	4.63	0.15	6.74	0.96	10.32	1.40	5.99	0.45	9.08	1.24
GE1568879	NM_029636.2	cathepsin Q	Ctsq	0.13	0.05	0.11	0.06	0.10	0.01	0.17	0.13	0.11	0.09	0.05	0.05
GE38157	NM_022326.2	cathepsin M	Ctsm	0.09	0.06	0.07	0.06	0.05	0.03	0.12	0.08	0.09	0.05	0.17	0.08
GE120174	NM_019541.2	cathepsin 8	Cts8	0.22	0.03	0.14	0.10	0.14	0.08	0.27	0.11	0.22	0.05	0.20	0.03
GE35272	NM_009984.2	cathepsin L	Ctsl	27.88	3.53	21.36	2.40	22.85	1.75	31.37	6.61	27.09	0.83	27.12	2.68
GE34913	NM_007800.1	cathepsin G	Ctsg	3.48	0.38	3.67	0.10	3.75	0.08	4.32	0.41	3.84	0.04	3.67	2.12
GE36392	NM_009985.2	cathepsin W	Ctsw	0.21	0.03	0.15	0.06	0.10	0.09	0.20	0.11	0.18	0.08	0.17	0.04
GE34912	NM_007799.2	cathepsin E	Ctse	0.78	0.21	0.64	0.07	0.58	0.09	1.00	0.11	0.94	0.07	0.62	0.21
GE37629	NM_019861.1	cathepsin F	Ctsf	5.28	0.13	5.40	0.07	5.31	0.49	7.57	1.17	7.60	0.53	5.92	1.06

Supplementary table 7. Cont'd….

Substance	ACCN#	Description	Gene Symbol	(Gene expression) 2 weeks CTL Average	SD	Hom-CKO Average	SD	Het-CKO Average	SD	(Gene expression) 3 weeks CTL Average	SD	Hom-CKO Average	SD	Het-CKO Average	SD
GE122943	NM_026906.2	cathepsin 3	Cts3	0.85	0.49	0.96	0.56	1.18	0.10	0.94	0.05	1.00	0.06	1.09	0.06
GE1499721	NM_177662.1	cathepsin O	Ctso	0.24	0.03	0.23	0.13	0.30	0.04	0.30	0.04	0.32	0.06	0.22	0.10
GE37963	NM_021445.1	cathepsin 6	Cts6	1.36	0.31	1.28	0.14	1.50	0.13	1.55	0.19	1.84	0.08	1.61	0.10
GE38185	NM_022325.3	cathepsin Z	Ctsz	9.21	1.00	9.52	0.31	8.44	0.69	10.54	1.65	13.41	1.11	8.31	1.58
GE119616	NM_012007.1	cathepsin J	Ctsj	0.20	0.11	0.31	0.05	0.35	0.03	0.20	0.10	0.29	0.05	0.25	0.10
GE120164	NM_019539.2	cathepsin 7	Cts7	0.16	0.02	0.15	0.05	0.19	0.09	0.10	0.06	0.15	0.06	0.26	0.03
GE37739	NM_020284.1	cathepsin R	Ctsr	0.15	0.13	0.18	0.06	0.13	0.02	0.11	0.11	0.23	0.06	0.10	0.07
GE36415	NM_010684.1	lysosomal membrane glycoprotein 1	Lamp1	46.44	1.26	41.60	1.08	42.84	3.26	54.06	4.07	53.42	1.88	44.02	1.55
GE35667	NM_010685.2	lysosomal membrane glycoprotein 2	Lamp2	19.88	3.35	15.40	0.19	15.56	1.17	17.67	1.71	18.20	0.88	13.70	1.19
GE1394674	NM_177356.2	lysosomal-associated membrane protein 3	Lamp3	1.34	0.55	1.24	0.08	1.46	0.23	1.46	0.20	1.30	0.29	1.55	0.14
GE38234	NM_022887.1	tuberous sclerosis 1	Tsc1	0.49	0.01	0.59	0.02	0.51	0.02	0.60	0.11	0.70	0.02	0.44	0.23
GE36243	NM_011647.1	tuberous sclerosis 2	Tsc2	17.25	0.90	18.00	0.13	16.21	1.68	19.84	1.01	18.08	1.95	19.98	3.63
GE42001	NM_053075.2	RAS-homolog enriched in brain	Rheb	62.58	5.53	56.39	1.78	52.20	1.97	77.92	9.97	62.19	1.98	66.27	1.96
GE102631	NM_026967.3	Ras homolog enriched in brain like 1	Rhebl1	1.08	0.33	0.73	0.05	0.87	0.04	1.39	0.51	1.03	0.20	1.36	0.32
GE42743	NM_016787.2	BCL2/adenovirus E1B 19kDa-interacting protein 1, NIP2	Bnip2	40.03	4.44	32.74	1.86	32.13	1.28	40.65	2.36	35.71	3.21	37.48	3.03
GE35123	NM_009760.2	BCL2/adenovirus E1B 19kDa-interacting protein 1, NIP3	Bnip3	37.57	8.86	30.90	0.79	31.06	3.23	51.54	1.29	47.15	2.00	43.99	0.68
GE128775	NM_134253.1	BCL2/adenovirus E1B 19kD interacting protein like	Bnipl	0.10	0.03	0.10	0.01	0.13	0.03	0.11	0.00	0.12	0.10	0.10	0.06
GE40971	NM_025842.1	vacuolar protein sorting 28 (yeast)	Vps28	34.53	5.12	28.35	1.54	27.92	0.13	48.11	8.85	32.86	2.82	34.80	5.28
GE102713	NM_126165.1	vacuolar protein sorting 4a (yeast)	Vps4a	4.31	0.09	3.88	0.33	3.80	0.30	6.09	1.66	4.32	0.18	4.68	0.75
GE126955	NM_133672.1	vacuolar protein sorting 26 (yeast)	Vps26	19.58	2.82	17.44	0.93	16.07	1.32	21.29	1.71	15.86	0.78	17.33	1.26
GE40989	NM_030559.2	vacuolar protein sorting 16 (yeast)	Vps16	2.46	0.04	2.72	0.38	2.50	0.03	3.13	0.44	2.50	0.18	2.35	0.25
GE102627	NM_145633.2	ankyrin repeat domain 27 (VPS9 domain)	Ankrd27	3.58	0.96	3.12	0.04	3.91	0.32	3.98	0.52	3.18	0.09	3.86	0.36
GE1415166	NM_178070.2	vacuolar protein sorting 33B (yeast)	Vps33b	2.01	0.16	2.09	0.38	1.91	0.13	2.53	0.24	2.05	0.10	2.07	0.29
GE1534364	NM_147153.2	vacuolar protein sorting 39 (yeast)	Vps39	1.23	0.14	1.09	0.13	0.90	0.05	1.25	0.40	1.03	0.22	1.09	0.34
GE33667	NM_009190.1	vacuolar protein sorting 4b (yeast)	Vps4b	3.15	0.29	3.18	0.28	2.74	0.08	3.22	0.34	2.72	0.36	2.74	0.14
GE42789	NM_019780.1	vacuolar protein sorting 29 (S pombe)	Vps29	49.55	13.59	46.04	3.28	37.09	1.61	63.52	10.11	53.76	3.30	57.18	12.00
GE137259	NM_172120.2	vacuolar protein sorting 41 (yeast)	Vps41	8.85	0.35	8.44	0.21	8.23	0.18	8.82	0.60	7.52	0.54	7.23	0.48
GE120817	NM_022997.2	vacuolar protein sorting 35	Vps35	34.82	8.66	32.78	0.65	30.45	1.24	38.74	2.45	35.05	0.39	31.49	2.38
GE39898	NM_025783.2	vacuolar protein sorting 24 (yeast)	Vps24	14.68	3.10	12.50	0.50	10.96	0.21	15.90	0.89	14.63	0.77	11.97	1.92
GE42173	NM_025696.1	sortilin-related VPS10 domain containing receptor 3	Sorcs3	0.41	0.10	0.27	0.05	0.42	0.07	0.36	0.06	0.34	0.12	0.31	0.15
GE40268	NM_029929.2	vacuolar protein sorting 33A (yeast)	Vps33a	1.37	0.11	1.57	0.12	1.66	0.06	1.53	0.16	1.54	0.14	1.28	0.08
GE41987	NM_139061.2	vacuolar protein sorting 54 (yeast)	Vps54	8.20	1.93	8.00	0.73	6.73	0.29	7.80	0.24	8.31	0.67	7.67	0.17
GE41556	NM_030889.1	sortilin-related VPS10 domain containing receptor 2	Sorcs2	2.53	0.28	2.76	0.34	3.50	0.15	3.20	0.43	3.47	0.36	3.64	0.75
GE137354	NM_172269.2	vacuolar protein sorting 18 (yeast)	Vps18	4.10	0.03	3.62	0.34	3.66	0.36	4.39	0.51	4.77	0.38	4.28	0.65
GE1392741	NM_139061.2	vacuolar protein sorting 54 (yeast)	Vps54	1.89	0.28	2.03	0.18	2.06	0.20	1.68	0.28	1.86	0.25	1.91	0.16
GE132274	NM_145633.2	ankyrin repeat domain 27 (VPS9 domain)	Ankrd27	0.65	0.02	0.71	0.14	0.56	0.03	0.54	0.13	0.73	0.10	0.55	0.17
GE36822	NM_013841.1	vacuolar protein sorting 45 (yeast)	Vps45	4.51	0.63	5.38	0.51	4.06	0.12	4.47	0.84	6.93	0.58	4.18	0.44
GE38200	NM_021377.1	VPS10 domain receptor protein SORCS 1	Sorcs1	0.25	0.10	0.23	0.03	0.30	0.03	0.17	0.03	0.35	0.06	0.29	0.03
GE136626	NM_153139.3	solute carrier family 36 (proton/amino acid symporter), member 1	Slc36a1	0.64	0.03	0.69	0.06	0.63	0.07	0.80	0.07	0.75	0.06	0.69	0.02
GE137404	NM_172289.3	solute carrier family 36 (proton/amino acid symporter), member 4	Slc36a4	0.89	0.02	0.83	0.02	0.73	0.04	0.68	0.11	0.70	0.11	0.67	0.08

Supplementary table 8. List of hand-curated “Vesicle trafficking” genes used for Fisher’s Exact test

Substance	ACCN#	Description	Gene Symbol	(Gene expression) 2 weeks						(Gene expression) 3 weeks					
				CTL		Hom-CKO		Het-CKO		CTL		Hom-CKO		Het-CKO	
				Average	SD	Average	SD	Average	SD	Average	SD	Average	SD	Average	SD
GE42665	NM_011342.2	SEC22 vesicle trafficking protein-like 1 (S cerevisiae)	Sec22l	31.39	3.95	25.92	0.98	25.14	1.24	32.46	3.59	30.56	1.88	27.26	2.22
GE1429758	NM_009204.1	solute carrier family 2 (facilitated glucose transporter), member 4	Slc2a	26.48	6.54	18.68	1.04	27.31	0.19	51.61	10.68	28.76	16.63	36.36	5.96
GE118555	NM_009498.3	vesicle-associated membrane protein 3	Vamp	13.89	0.58	12.34	0.20	12.28	0.41	15.19	0.06	11.76	1.11	12.60	0.64
GE33013	NM_010414.1	Huntington disease gene homolog	Hdh	1.00	0.07	1.29	0.16	1.01	0.10	1.13	0.15	1.48	0.15	1.09	0.20
GE39079	NM_133704.3	SEC22 vesicle trafficking protein-like 2 (S cerevisiae)	Sec22l	7.48	1.71	6.12	0.27	5.84	0.13	9.11	0.67	6.97	0.96	6.68	1.01
GE37073	NM_016800.2	vesicle transport through interaction with t-SNAREs 1B homolog	Vti1b	36.00	3.42	33.25	1.66	30.90	2.24	45.73	5.19	37.50	3.72	36.87	3.42
GE35083	NM_009696.2	apolipoprotein E	Apoe	35.85	11.10	27.97	1.63	26.44	3.83	58.95	3.46	43.33	4.65	40.22	9.97
GE37072	NM_016794.2	vesicle-associated membrane protein 8	Vamp	13.40	4.68	11.82	1.34	9.80	0.44	16.68	2.98	13.75	1.19	13.78	3.70
GE34995	NM_007912.3	epidermal growth factor receptor , transcript variant 2	Egfr	0.75	0.10	0.81	0.01	0.68	0.03	0.94	0.03	0.90	0.06	0.85	0.24
GE33696	NM_009222.2	synaptosomal-associated protein 23	Snap	8.23	1.12	7.13	0.22	7.96	1.14	12.04	0.88	7.49	0.61	8.68	1.06
GE137019	NM_153579.3	synaptic vesicle glycoprotein 2 b	Sv2b	0.14	0.07	0.10	0.02	0.13	0.05	0.27	0.10	0.09	0.07	0.14	0.03
GE34275	NM_008943.1	presenilin 1	Psen	4.59	0.63	5.36	0.21	4.33	0.11	5.74	0.74	4.91	0.34	4.22	0.27
GE34131	NM_008740.2	N-ethylmaleimide sensitive fusion protein	Nsf	2.21	0.38	2.45	0.20	2.31	0.20	2.38	0.40	1.95	0.25	1.83	0.29
GE35778	NM_010864.1	myosin Va	Myo5a	0.60	0.11	0.65	0.01	0.52	0.01	0.69	0.27	0.72	0.06	0.62	0.07
GE42898	NM_010578.1	integrin beta 1 (fibronectin receptor beta)	Itgb	10.79	1.64	9.57	2.39	8.64	0.35	10.08	1.28	8.69	0.38	8.94	1.06
GE35054	NM_009652.1	thymoma viral proto-oncogene 1	Akt	15.11	0.71	15.15	0.63	12.93	0.49	16.44	1.49	12.86	1.77	13.97	0.93
GE109052	NM_009170.2	sonic hedgehog	Shh	0.10	0.09	0.06	0.04	0.07	0.02	0.10	0.07	0.11	0.02	0.06	0.05
GE34315	NM_009001.2	RAB3A, member RAS oncogene family	Rab3a	11.43	1.86	9.35	0.30	11.51	0.89	16.33	3.58	8.40	0.62	14.19	3.06
GE1453040	NM_009911.2	chemokine (C-X-C motif) receptor 4	Cxcr	8.64	2.67	7.11	0.35	7.66	1.19	6.71	1.78	3.88	0.69	5.09	0.38
GE36528	NM_013454.2	ATP-binding cassette, sub-family A (ABC1), member 1	Abca	3.41	0.56	2.98	0.29	3.33	0.11	1.99	0.32	1.74	0.24	1.94	0.34
GE42383	NM_023126.2	RAB8A, member RAS oncogene family	Rab8a	4.28	0.73	4.45	0.23	3.85	0.31	4.71	0.65	4.25	0.28	3.93	0.75
GE42757	NM_017382.2	RAB11a, member RAS oncogene family	Rab11a	67.66	11.71	60.45	1.64	54.26	1.18	68.62	8.93	57.92	2.40	52.81	8.51
GE40996	NM_025887.2	RAB5A, member RAS oncogene family	Rab5a	9.00	2.12	8.95	0.38	8.37	0.50	8.67	0.39	9.31	1.19	7.65	0.13
GE40540	NM_023635.2	RAB27A, member RAS oncogene family	Rab27a	0.25	0.08	0.12	0.01	0.14	0.08	0.36	0.05	0.23	0.05	0.20	0.04
GE102589	NM_026912.1	sorting nexin 15	Snx	33.41	0.48	30.08	2.31	28.30	1.51	37.74	6.55	26.46	2.42	29.56	3.16
GE38220	NM_022813.2	secretory carrier membrane protein 2	Scamp	5.93	0.28	6.84	0.13	5.67	0.51	7.06	1.66	6.80	0.37	5.34	0.48
GE131355	NM_145450.2	cDNA sequence BC022687	BC	0.67	0.21	0.55	0.06	0.50	0.03	0.69	0.10	0.89	0.12	0.70	0.11
GE42460	NM_007480.1	ADP-ribosylation factor 5	Arf	20.03	2.72	21.81	1.72	17.46	0.62	21.63	2.04	21.37	0.24	16.42	1.76
GE39161	NM_080557.1	sorting nexin 4	Snx	0.17	0.05	0.19	0.04	0.28	0.07	0.12	0.15	0.22	0.04	0.09	0.05
GE138183	NM_172804.1	synaptotagmin 14-like	Syt14l	1.27	0.07	1.50	0.03	1.67	0.08	1.44	0.02	1.51	0.11	1.53	0.18
GE109411	NM_175122.2	RAB39B, member RAS oncogene family	Rab39b	1.40	0.04	1.37	0.12	1.32	0.04	1.42	0.07	1.41	0.12	1.41	0.05
GE37153	NM_016899.2	RAB25, member RAS oncogene family	Rab	0.16	0.07	0.19	0.09	0.19	0.03	0.20	0.13	0.19	0.04	0.24	0.07
GE34758	NM_007479.2	ADP-ribosylation factor 4	Arf	43.19	10.45	43.13	1.62	36.25	2.02	43.96	3.47	42.62	1.37	36.97	1.46
GE42213	NM_021710.1	adaptor-related protein complex AP-4, sigma 1	Ap4s	31.44	7.30	25.44	2.50	24.16	2.40	46.25	7.00	33.10	3.71	33.53	4.16
GE39363	NM_027135.2	SEC24 related gene family, member D (S cerevisiae)	Sec24d	8.17	0.72	7.73	0.19	7.23	0.12	8.30	0.83	6.05	0.61	6.51	0.42
GE37107	NM_016857.1	exocyst complex component 7	Exoc	3.09	0.60	3.20	0.16	3.00	0.08	4.98	1.43	3.41	0.41	3.25	0.53
GE35073	NM_009682.2	adaptor-related protein complex 3, sigma 2 subunit	Ap3s	2.59	0.12	2.94	0.22	2.48	0.18	3.01	0.47	2.93	0.06	2.77	0.04
GE102627	NM_145633.2	ankyrin repeat domain 27 (VPS9 domain)	Ankrd	3.58	0.96	3.12	0.04	3.91	0.32	3.98	0.52	3.18	0.09	3.86	0.36
GE1421993	NM_178278.2	calcyphosphine 2	Caps	0.09	0.05	0.07	0.03	0.10	0.05	0.07	0.01	0.07	0.04	0.07	0.03
GE136342	NM_148925.1	FYVE and coiled-coil domain containing 1	Fyco	16.68	3.14	14.01	0.42	17.94	0.91	18.59	2.24	11.90	0.45	18.01	1.88
GE41226	NM_026405.2	RAB32, member RAS oncogene family	Rab	4.65	0.71	4.82	0.33	4.82	0.11	5.70	0.36	5.38	0.08	5.48	0.30
GE102713	NM_126165.1	vacuolar protein sorting 4a (yeast)	Vps4a	4.31	0.09	3.88	0.33	3.80	0.30	6.09	1.66	4.32	0.18	4.68	0.75
GE132621	NM_145929.1	golgi associated, gamma adaptin ear containing, ARF binding protein 1	Gga	5.99	0.11	6.06	0.28	5.50	0.65	5.73	0.38	5.53	0.43	6.44	1.91
GE37010	NM_010261.1	Rab acceptor 1 (prenylated)	Rabac	90.54	12.47	74.98	1.77	73.33	2.29	105.62	2.89	88.17	7.25	87.39	8.92
GE36869	NM_015803.1	ATPase, aminophospholipid transporter-like, class I, type 8A, member 2	Atp8a	0.30	0.10	0.27	0.03	0.36	0.02	0.20	0.10	0.46	0.05	0.24	0.07
GE38259	NM_023051.1	calsyntenin 1	Clstn	0.45	0.14	0.44	0.10	0.38	0.06	0.42	0.17	0.42	0.13	0.48	0.12
GE137075	NM_153680.1	sorting nexin 17	Snx	13.47	0.40	13.58	0.50	14.38	0.16	15.87	0.61	15.17	1.28	13.56	0.36
GE37606	NM_019727.1	sorting nexin 1	Snx	3.25	0.38	2.94	0.16	2.73	0.10	4.61	1.16	2.79	0.23	3.14	0.38
GE33146	NM_009938.2	coatomer protein complex subunit alpha	Copa	15.19	0.45	14.63	0.76	15.14	0.64	15.82	0.84	12.71	0.75	12.35	0.04
GE41897	NM_033523.2	sprouty protein with EVH-1 domain 2, related sequence	Spred	4.52	0.32	5.09	0.53	6.07	0.40	5.20	0.53	5.34	0.34	6.16	1.42
GE40023	NM_026959.1	syntaxin 18	Stx	3.71	0.36	3.38	0.16	3.42	0.02	4.85	0.69	3.84	0.18	3.71	0.39
GE37771	NM_008385.3	inositol polyphosphate-5-phosphatase B	Inpp5b	2.01	0.05	2.18	0.13	2.03	0.20	2.10	0.07	1.84	0.09	1.54	0.05
GE1463744	NM_024454.1	RAB21, member RAS oncogene family	Rab	9.35	0.48	8.22	0.39	8.72	0.19	10.46	0.45	8.27	0.46	8.10	0.31
GE1427678	NM_145219.2	leucine-rich repeat LGI family, member 3	Lgi	0.84	0.10	0.67	0.07	0.83	0.04	0.93	0.20	0.83	0.11	1.08	0.28
GE42323	NM_026677.1	RAB13, member RAS oncogene family	Rab	2.45	0.32	2.18	0.21	2.11	0.28	2.27	0.30	1.76	0.18	1.65	0.25
GE41510	NM_030695.1	LPS-responsive beige-like anchor	Lrba	2.65	0.22	2.39	0.12	2.37	0.25	2.92	0.43	3.10	0.37	2.61	0.53

Supplementary table 8. Cont'd….

				(Gene expression) 2 weeks						(Gene expression) 3 weeks					
Substance	ACCN#	Description	Gene Symbol	CTL		Hom-CKO		Het-CKO		CTL		Hom-CKO		Het-CKO	
				Average	SD	Average	SD	Average	SD	Average	SD	Average	SD	Average	SD
GE42558	NM_009003.1	RAB4A, member RAS oncogene family	Rab4a	2.06	0.22	1.80	0.10	1.78	0.10	2.23	0.49	1.38	0.07	1.77	0.24
GE1437572	BC046444.1	myosin Vb, mRNA (cDNA clone IMAGE:3600554)	Myo5b	0.29	0.08	0.43	0.03	0.63	0.08	0.55	0.05	0.54	0.05	0.56	0.06
GE37843	NM_020578.1	EH-domain containing 3	Ehd	14.16	1.33	15.77	0.44	15.13	0.62	14.62	0.80	15.46	0.82	15.35	0.85
GE41552	NM_028733.1	protein kinase C and casein kinase substrate in neurons 3	Pacsin	27.89	4.35	25.46	0.87	27.22	0.57	41.13	8.13	28.35	2.50	29.58	3.62
GE130309	NM_144895.1	spastic paraplegia 20, spartin (Troyer syndrome) homolog (human)	Spg	2.29	0.41	2.11	0.31	2.07	0.05	2.92	0.65	1.98	0.16	2.08	0.32
GE127423	NM_133838.2	EH-domain containing 4	Ehd	81.64	7.79	77.66	6.17	74.53	1.43	104.80	13.07	115.07	18.56	88.43	11.45
GE33324	NM_009618.1	a disintegrin and metalloprotease domain 2	Adam	0.09	0.08	0.11	0.02	0.07	0.01	0.09	0.05	0.12	0.04	0.13	0.08
GE40857	NM_181072.2	myosin IE	Myo1e	0.55	0.28	0.88	0.08	0.75	0.03	0.48	0.25	0.57	0.06	0.44	0.10
GE42861	NM_025898.1	N-ethylmaleimide sensitive fusion protein attachment protein alpha	Napa	4.72	0.73	4.62	0.81	4.18	0.29	7.85	3.30	5.39	0.24	5.69	0.39
GE36399	NM_010119.3	EH-domain containing 1	Ehd	26.11	1.39	19.40	1.06	22.73	0.85	27.10	4.13	20.74	1.23	22.82	1.22
GE1413075	NM_181414.3	phosphoinositide-3-kinase, class 3	Pik3c	4.51	0.23	3.98	0.19	3.98	0.25	4.57	0.42	3.99	0.01	3.69	0.23
GE37093	NM_016887.2	claudin 7	Cldn	0.37	0.07	0.52	0.05	0.43	0.02	0.52	0.07	0.63	0.37	0.42	0.04
GE34406	NM_008027.1	flotillin 1	Flot	16.30	1.81	15.66	0.32	15.43	0.56	27.43	7.06	18.95	1.89	21.16	0.40
GE122705	NM_026697.2	RAB14, member RAS oncogene family	Rab	40.03	1.78	35.13	4.07	36.47	3.96	44.80	3.15	38.91	0.23	37.43	1.13
GE42785	NM_019749.3	gamma-aminobutyric acid receptor associated protein	Gabarap	39.03	8.00	30.62	0.95	29.74	1.16	50.85	8.05	37.82	2.43	39.00	1.65
GE41486	NM_010623.2	kinesin family member 17	Kif	0.01	0.04	0.05	0.02	0.08	0.00	0.12	0.08	0.13	0.05	0.08	0.08
GE38187	NM_007379.1	ATP-binding cassette, sub-family A (ABC1), member 2	Abca	10.79	1.50	10.27	0.73	10.47	0.19	13.42	3.28	10.93	1.42	10.80	1.70
GE36997	NM_016676.2	RAB10, member RAS oncogene family	Rab	8.98	0.77	9.57	0.61	8.58	0.56	12.08	3.03	9.83	0.44	8.72	0.86
GE34518	NM_008168.1	glutamate receptor, ionotropic, kainate 5 (gamma 2)	Grik	5.87	0.35	7.74	1.16	10.05	1.06	7.55	0.82	9.09	0.79	11.22	0.51
GE42599	NM_009668.1	bridging integrator 1	Bin	2.65	0.13	2.69	0.21	2.60	0.16	3.37	0.14	2.41	0.36	2.64	0.33
GE36635	NM_013540.1	glutamate receptor, ionotropic, AMPA2 (alpha 2)	Gria	2.17	0.34	2.13	0.20	1.76	0.37	1.65	0.27	1.94	0.31	2.19	0.36
GE42449	NM_011400.1	solute carrier family 2 (facilitated glucose transporter), member 1	Slc2a	0.40	0.04	0.48	0.12	0.39	0.05	0.75	0.17	1.31	0.34	0.56	0.06
GE38076	NM_021789.2	trafficking protein particle complex 4	Trappc	17.86	3.12	17.38	0.64	15.07	1.47	19.79	3.47	19.40	0.81	15.91	1.56
GE36561	NM_013718.2	trafficking protein particle complex 3	Trappc	11.36	0.27	12.80	0.63	10.64	0.24	13.23	0.67	12.62	0.65	10.35	0.37
GE38135	NM_022017.1	transient receptor potential cation channel, subfamily V, member 4	Trpv	0.60	0.08	0.56	0.06	0.65	0.06	0.79	0.11	0.68	0.02	0.75	0.09
GE38539	NM_016900.2	caveolin 2	Cav	29.43	8.04	22.46	1.51	24.51	0.64	26.14	10.30	15.64	0.84	23.32	5.25
GE34911	NM_007798.1	cathepsin B	Ctsb	8.52	0.57	8.07	0.65	7.83	0.06	10.62	2.37	10.41	0.96	9.40	1.17
GE33744	NM_009294.2	syntaxin 4A (placental)	Stx4a	24.44	0.73	21.78	1.29	19.21	1.56	29.35	2.08	27.41	2.13	21.81	1.71
GE40898	NM_025701.2	trafficking protein particle complex 5	Trappc	20.21	3.25	17.28	0.99	15.25	0.62	23.53	2.77	19.98	0.68	18.06	0.80
GE37719	NM_019972.2	sortilin 1	Sort	0.91	0.10	0.85	0.04	0.69	0.04	0.67	0.05	0.70	0.03	0.66	0.11
GE36101	NM_011392.1	solute carrier family 34 (sodium phosphate), member 1	Slc34a	0.62	0.06	0.73	0.13	0.79	0.09	0.69	0.03	0.75	0.06	0.78	0.15
GE36107	NM_012030.1	solute carrier family 9 (sodium/hydrogen exchanger), isoform 3 regulator 1	Slc9a3r	2.29	1.14	1.54	0.05	1.59	0.15	2.37	0.39	1.48	0.09	1.69	0.31
GE34056	NM_008642.1	microsomal triglyceride transfer protein	Mttp	0.66	0.08	0.92	0.17	0.66	0.07	0.77	0.13	0.78	0.07	0.61	0.08
GE33671	NM_008732.1	solute carrier family 11 (proton-coupled divalent metal ion transporters), member 2	Slc11a	3.36	0.64	4.10	0.25	3.59	0.34	4.63	1.31	4.48	0.65	3.52	0.81
GE38067	NM_021712.1	solute carrier family 18 (vesicular monoamine), member 3	Slc18a	0.12	0.04	0.06	0.07	0.14	0.02	0.10	0.10	0.10	0.10	0.08	0.06
GE34313	NM_008996.2	RAB1, member RAS oncogene family	Rab	51.95	3.79	45.66	2.60	41.10	2.44	58.63	6.39	45.48	2.44	53.46	2.05
GE33737	NM_009287.2	stromal interaction molecule 1	Stim	1.39	0.07	1.54	0.12	1.50	0.12	1.54	0.08	1.55	0.31	1.32	0.09
GE36523	NM_011246.2	RAS guanyl releasing protein 1	Rasgrp	0.09	0.09	0.10	0.02	0.10	0.03	0.13	0.02	0.08	0.06	0.10	0.13
GE34317	NM_009005.1	RAB7, member RAS oncogene family	Rab	55.45	3.18	49.76	3.37	48.73	2.79	77.79	13.04	55.06	7.09	60.80	3.41
GE33754	NM_009308.2	synaptotagmin 4	Syt	0.30	0.20	0.26	0.11	0.31	0.02	0.25	0.15	0.25	0.06	0.18	0.06
GE34757	NM_007476.2	ADP-ribosylation factor 1	Arf	78.98	2.11	72.41	1.60	69.46	4.49	92.78	4.91	76.59	8.86	83.38	6.82
GE120774	NM_022025.3	solute carrier family 5 (choline transporter), member 7	Slc5a	0.09	0.07	0.23	0.10	0.20	0.04	0.20	0.10	0.21	0.10	0.18	0.06
GE37381	NM_019488.2	solute carrier family 2, (facilitated glucose transporter), member 8	Slc2a	1.87	0.42	2.00	0.28	1.81	0.14	2.37	0.56	2.27	0.08	1.97	0.32
GE36159	NM_011504.1	syntaxin binding protein 3	Stxbp	5.27	0.63	4.69	0.13	4.48	0.22	5.06	0.34	4.74	0.09	4.71	0.20
GE33064	NM_011502.1	syntaxin 3, transcript variant C	Stx3	0.52	0.12	0.46	0.14	0.41	0.11	0.35	0.09	0.38	0.02	0.44	0.20
GE35138	NM_012061.1	Ca<2+>dependent activator protein for secretion	Cadps	0.13	0.08	0.11	0.05	0.11	0.06	0.12	0.11	0.13	0.06	0.13	0.02
GE1542522	NM_173413.2	RAB8B, member RAS oncogene family	Rab8b	0.04	0.03	0.06	0.03	0.05	0.04	0.07	0.06	0.07	0.06	0.07	0.03
GE36098	NM_011977.2	solute carrier family 27 (fatty acid transporter), member 1	Slc27a	10.46	3.01	9.23	1.00	10.75	1.07	11.38	3.52	8.64	0.62	8.99	1.59
GE41394	NM_028238.5	Rab38, member of RAS oncogene family	Rab	0.64	0.05	0.56	0.06	0.63	0.02	0.74	0.09	0.68	0.02	0.73	0.06
GE121691	NM_025735.1	microtubule-associated protein 1 light chain 3 alpha	Map1lc3a	154.98	25.90	115.61	6.01	131.39	3.05	217.00	21.81	135.63	11.35	179.85	21.84
GE1571047	NM_198862.1	neuroligin 2	Nlgn	7.54	0.72	7.34	1.20	6.72	0.45	4.89	0.59	5.03	0.41	3.56	0.18
GE35117	NM_009748.2	blocked early in transport 1 homolog (S cerevisiae)	Bet	0.98	0.31	0.71	0.03	0.82	0.18	0.96	0.19	0.86	0.16	0.94	0.15
GE36723	NM_013626.1	peptidylglycine alpha-amidating monooxygenase	Pam	56.76	2.39	64.46	1.80	44.22	4.89	74.98	8.45	90.80	9.30	55.18	1.88
GE119531	NM_011861.1	protein kinase C and casein kinase substrate in neurons 1	Pacsin	1.40	0.23	1.71	0.19	2.16	0.12	1.46	0.27	1.43	0.03	1.36	0.05
GE36043	NM_011326.1	sodium channel, nonvoltage-gated 1 gamma	Scnn1g	0.31	0.08	0.41	0.10	0.43	0.04	0.36	0.05	0.51	0.02	0.40	0.06
GE35325	NM_009955.2	dihydropyrimidinase-like 2	Dpysl	0.82	0.08	0.84	0.11	0.78	0.06	0.76	0.04	0.81	0.14	0.79	0.13

Supplementary table 8. Cont'd….

Substance	ACCN#	Description	Gene Symbol	(Gene expression) 2 weeks						(Gene expression) 3 weeks					
				CTL		Hom-CKO		Het-CKO		CTL		Hom-CKO		Het-CKO	
				Average	SD	Average	SD	Average	SD	Average	SD	Average	SD	Average	SD
GE34223	NM_008869.2	phospholipase A2, group IVA (cytosolic, calcium-dependent)	Pla2g4a	4.88	2.76	4.47	0.21	3.55	0.38	5.54	2.13	7.26	0.28	4.78	1.07
GE137683	NM_172523.1	solute carrier family 18 (vesicular monoamine), member 2	Slc18a	1.12	0.03	1.19	0.30	1.31	0.02	1.30	0.05	1.36	0.03	1.41	0.06
GE36327	NM_011792.3	beta-site APP cleaving enzyme 1	Bace	4.25	0.38	4.95	0.38	4.72	0.41	3.92	0.76	5.05	0.13	3.98	0.48
GE34520	NM_008170.2	glutamate receptor, ionotropic, NMDA2A (epsilon 1)	Grin2a	0.10	0.04	0.07	0.03	0.14	0.02	0.10	0.07	0.02	0.07	0.10	0.05
GE34494	NM_008138.2	guanine nucleotide binding protein, alpha inhibiting 2	Gnai	50.19	1.35	46.38	1.67	41.33	3.03	50.10	9.04	38.03	4.42	41.36	1.71
GE42716	NM_013681.1	synapsin II	Syn	0.46	0.06	2.08	0.17	0.49	0.05	0.69	0.07	4.06	0.66	0.55	0.07
GE40371	NM_023129.3	phospholamban	Pln	364.19	17.64	283.70	28.88	335.20	22.53	493.85	20.11	275.30	16.13	386.61	5.89
GE34056	NM_008642.1	microsomal triglyceride transfer protein	Mttp	0.66	0.08	0.92	0.17	0.66	0.07	0.77	0.13	0.78	0.07	0.61	0.08
GE33080	NM_010306.1	guanine nucleotide binding protein, alpha inhibiting 3	Gnai	5.04	0.39	4.98	0.92	4.56	0.26	4.34	1.14	3.60	0.13	3.93	0.23
GE1569336	NM_173440.1	nuclear receptor interacting protein 1	Nrip	5.04	1.16	5.23	0.42	5.30	0.51	4.56	0.30	4.41	0.21	4.25	1.10
GE35250	NM_009946.1	complexin 2	Cplx	5.96	0.67	5.54	0.59	4.86	0.20	5.48	0.50	5.49	0.46	4.98	0.34
GE37300	NM_018801.1	synaptotagmin 7	Syt	0.13	0.06	0.24	0.01	0.21	0.10	0.28	0.08	0.33	0.06	0.41	0.06
GE34962	NM_007871.1	dynamin 2	Dnm	6.91	1.48	7.00	0.51	6.85	0.18	8.02	1.89	7.00	0.81	6.74	1.10
GE40657	NM_025285.1	stathmin-like 2	Stmn	1.15	0.04	1.08	0.05	1.10	0.06	1.18	0.07	0.86	0.09	0.95	0.20
GE37596	NM_019787.2	SEC23B (S cerevisiae)	Sec23b	2.65	0.24	2.61	0.20	2.36	0.17	2.96	0.38	2.10	0.05	2.52	0.13
GE33634	NM_009147.1	SEC23A (S cerevisiae)	Sec23a	0.48	0.13	0.48	0.07	0.41	0.16	0.61	0.09	0.42	0.10	0.42	0.04
GE39363	NM_027135.2	SEC24 related gene family, member D (S cerevisiae)	Sec24d	8.17	0.72	7.73	0.19	7.23	0.12	8.30	0.83	6.05	0.61	6.51	0.42
GE118339	NM_009120.1	SAR1a gene homolog 1 (S cerevisiae)	Sara	34.77	2.88	32.87	2.99	31.71	0.86	37.03	2.11	32.58	1.38	32.24	1.72
GE121432	NM_025535.1	SAR1a gene homolog 2 (S cerevisiae)	Sara	99.17	29.23	72.18	3.18	69.73	3.40	121.84	10.20	92.05	1.65	95.18	8.57
GE1435201	BC042701.1	Similar to secretory pathway component Sec31B-1, clone IMAGE:4485426, mRNA	Sec31b	2.06	0.06	1.85	0.11	1.97	0.13	2.55	0.25	2.03	0.24	2.41	0.18
GE35977	NM_011228.1	RAB33A, member of RAS oncogene family	Rab33a	1.13	0.13	1.07	0.02	1.07	0.11	0.98	0.22	1.31	0.08	1.22	0.23
GE37154	NM_016858.1	RAB33B, member of RAS oncogene family	Rab33b	12.07	1.56	13.29	1.59	13.88	0.22	14.69	0.62	13.06	1.96	14.47	3.34
GE38031	BC057328.1	coatomer protein complex, subunit epsilon, mRNA (cDNA clone IMAGE:6815918)	Cope	73.80	5.72	60.13	2.25	59.64	3.23	84.17	13.41	57.82	2.64	64.36	6.63
GE37628	NM_019877.1	coatomer protein complex, subunit zeta 2	Copz	3.40	0.87	2.68	0.36	2.77	0.17	4.06	0.81	2.80	0.26	2.96	0.36
GE42442	NM_015827.1	coatomer protein complex, subunit beta 2 (beta prime)	Copb	37.00	3.37	32.47	0.43	32.85	2.25	44.51	1.65	34.29	3.48	36.90	4.57
GE41872	NM_033370.2	coatomer protein complex, subunit beta 1	Copb	10.10	0.85	9.26	1.22	8.37	0.44	10.02	0.34	8.37	0.22	8.03	0.50
GE37486	NM_019817.1	coatomer protein complex, subunit zeta 1	Copz	9.95	0.94	12.19	0.36	9.72	0.39	14.13	1.49	13.68	0.72	10.78	1.79

Supplementary-table 9. List of hand-curated “Microtubule-related” genes used for Fisher’s Exact test.

				(Gene expression) 2 weeks						(Gene expression) 3 weeks					
Substance	ACCN#	Description	Gene Symbol	CTL		Hom-CKO		Het_CKO		CTL		Hom-CKO		Het_CKO	
				Average	SD	Average	SD	Average	SD	Average	SD	Average	SD	Average	SD
GE1549347	NM_178869.2	tubulin tyrosine ligase-like 1 (Ttll1), mRNA	Ttll1	9.04	1.69	2.14	0.11	7.16	0.23	17.49	1.97	2.70	0.26	12.41	1.38
GE1536765	NM_182839.1	Tubulin polymerization promoting protein	Tppp	5.04	0.80	2.18	0.12	4.35	0.37	3.82	0.30	1.80	0.06	4.31	0.52
GE1535637	NM_009446.1	tubulin, alpha 3	Tuba3	0.22	0.05	0.24	0.04	0.21	0.05	0.38	0.18	0.21	0.02	0.24	0.12
GE36449	NM_011655.2	tubulin, beta 5	Tubb5	35.19	2.54	37.87	0.94	28.14	1.86	43.82	5.47	25.23	1.82	33.05	3.44
GE42593	AA028214.1	mi17b02r1 Soares mouse p3NMF19.5 cDNA clone IMAGE:463755 5' similar to gb:M13446 Mouse alpha-tubulin isotype M-alpha-2 mRNA, complete cds (MOUSE);.	EST	61.69	7.21	51.08	2.96	46.84	2.59	66.75	9.66	43.82	4.98	50.00	7.02
GE40631	NM_009451.3	tubulin, beta 4	Tubb4	1.61	0.24	1.59	0.06	1.33	0.08	1.68	0.24	1.11	0.09	1.33	0.13
GE112664	NM_178869.2	tubulin tyrosine ligase-like 1, mRNA	Ttll1	2.13	0.14	1.75	0.12	2.02	0.11	2.58	0.28	1.85	0.06	1.96	0.22
GE127947	NM_134024.1	tubulin, gamma 1	Tubg1	8.61	0.31	8.80	0.23	8.39	0.83	11.38	1.47	8.16	0.21	11.70	1.79
GE37537	NM_019756.1	tubulin, delta 1	Tubd1	3.38	0.70	3.10	0.23	3.11	0.06	4.33	0.03	3.21	0.18	3.42	0.47
GE38926	NM_133755.1	tubulin, gamma complex associated protein 2	Tubgcp2	5.91	0.13	6.49	0.18	6.02	0.75	7.51	0.68	5.58	0.14	6.12	0.21
GE1423089	AK077097.1	coiled-coil domain containing 18	Ccdc185	2.90	0.47	2.69	0.19	3.03	0.34	3.32	0.62	2.54	0.28	2.80	0.11
GE1422462	AA896196.1	tubulin, alpha 1B	Tuba1b	0.61	0.05	0.63	0.06	0.82	0.05	0.70	0.06	0.54	0.12	0.63	0.08
GE102276	NM_133755.1	tubulin, gamma complex associated protein 2	Tubgcp2	5.29	0.49	5.06	0.03	5.16	0.19	6.04	1.23	4.75	0.15	5.22	0.11
GE37180	NM_017379.1	tubulin, alpha 8	Tuba8	13.35	1.92	13.25	2.30	11.53	0.70	28.69	8.69	23.17	3.42	23.51	6.19
GE1402949	NM_178385.2	tubulin-specific chaperone c	Tbcc	10.20	0.96	9.18	0.43	9.17	0.62	12.69	1.14	11.41	0.39	9.65	1.06
GE123224	NM_027192.1	tubulin tyrosine ligase	Ttl	2.46	0.31	2.61	0.08	2.32	0.21	3.01	0.53	2.71	0.32	2.47	0.25
GE1542538	BC057626.1	tubulin, gamma complex associated protein 6, mRNA	Tubgcp6	7.64	0.67	7.21	0.28	6.94	0.68	6.72	0.52	6.10	0.36	6.43	0.27
GE127966	NM_134028.1	tubulin, gamma 2	Tubg2	1.39	0.11	1.48	0.09	1.58	0.21	2.12	1.01	2.02	0.11	1.38	0.14
GE133705	NM_146190.2	tubulin, gamma complex associated protein 5	Tubgcp5	4.85	0.81	4.56	0.54	4.61	0.16	4.71	0.55	4.54	0.07	4.72	0.32
GE113066	NM_027192.1	tubulin tyrosine ligase	Ttl	2.80	0.15	2.96	0.18	2.34	0.16	3.51	0.24	3.49	0.17	3.00	0.38
GE33763	NM_009321.1	tubulin cofactor a	Tbca	29.04	13.97	22.71	3.44	21.44	1.78	24.91	3.54	25.02	1.64	22.80	5.81
GE1512638	NM_198031.1	tubulin, gamma complex associated protein 3	Tubgcp3	16.53	0.25	17.28	1.08	17.60	1.76	14.84	0.44	15.38	0.37	14.62	0.31
GE40417	NM_023279.2	tubulin, beta 3	Tubb3	0.49	0.07	0.35	0.06	0.37	0.03	0.39	0.07	0.41	0.09	0.45	0.05
GE42230	NM_080788.1	tau tubulin kinase 1	Ttbk1	0.72	0.12	0.71	0.03	0.68	0.22	0.53	0.08	0.60	0.04	0.62	0.20
GE1559506	AK083236.1	Tubulin tyrosine ligase-like family, member 7	Ttll7	1.01	0.11	1.45	0.17	0.86	0.03	1.06	0.01	2.60	0.37	1.07	0.20
GE39414	AK010005.1	epsilon-tubulin 1	Tube1	0.39	0.08	0.47	0.12	0.40	0.04	0.18	0.06	0.45	0.10	0.24	0.12
GE118528	NM_009446.1	tubulin, alpha 3	Tuba3	0.19	0.09	0.10	0.05	0.09	0.04	0.21	0.12	2.67	4.25	0.22	0.08
GE121691	NM_025735.1	microtubule-associated protein 1 light chain 3 alpha	Map1lc3a	154.98	25.90	115.61	6.01	131.39	3.05	217.00	21.81	135.63	11.35	179.85	21.84
GE41120	NM_026160.1	microtubule-associated protein 1 light chain 3 beta	Map1lc3b	58.89	1.43	54.57	6.36	50.64	3.74	70.76	1.80	49.26	3.08	53.54	1.71
GE36398	NM_007896.2	microtubule-associated protein, RP/EB family, member 1	Mapre1	10.45	1.60	8.80	0.74	8.53	0.48	9.60	1.69	7.43	0.61	8.49	1.14
GE38746	AK005562.1	echinoderm microtubule associated protein like 2	Eml2	9.45	2.15	8.76	1.11	8.99	0.57	9.78	1.37	7.63	0.19	7.54	0.61
GE112178	NM_172279.1	MAP/microtubule affinity-regulating kinase 4	Mark4	1.80	0.41	1.91	0.06	2.23	0.08	5.26	1.38	4.10	0.50	4.21	1.34
GE136452	NM_153058.2	microtubule-associated protein, RP/EB family, member 2	Mapre2	26.16	2.28	24.96	1.03	23.06	0.61	27.35	2.90	22.10	0.37	19.50	2.28
GE36359	NM_010838.2	microtubule-associated protein tau	Mapt	1.73	0.15	1.79	0.40	1.44	0.28	2.26	0.74	1.87	0.37	1.97	0.55
GE34048	NM_008632.1	microtubule-associated protein 2	Mtap2	0.22	0.04	0.15	0.11	0.17	0.00	0.16	0.06	0.14	0.02	0.10	0.04
GE102868	AF150755.1	microtubule-actin crosslinking factor mRNA	Macf	30.95	1.05	29.00	3.51	30.61	3.19	27.42	1.92	26.88	0.96	28.41	4.32
GE1417275	NM_199466.1	echinoderm microtubule associated protein like 4	Eml4	3.40	0.74	3.38	0.27	2.99	0.14	2.10	0.39	2.08	0.07	2.11	0.13
GE36422	NM_008633.1	microtubule-associated protein 4	Mtap4	20.00	1.10	21.90	0.72	17.72	1.00	20.61	1.01	20.38	1.23	18.76	1.31
GE34050	NM_008635.1	microtubule-associated protein 7	Mtap7	0.70	0.11	0.84	0.07	0.75	0.11	0.81	0.22	0.81	0.03	0.73	0.11
GE137377	NM_172279.1	MAP/microtubule affinity-regulating kinase 4	Mark4	1.48	0.25	1.67	0.21	1.99	0.15	3.20	0.97	3.21	0.44	3.17	0.47
GE1511208	BC053094.1	echinoderm microtubule associated protein like 1, mRNA , containing frame-shift s	Eml1	29.78	0.34	30.87	1.32	27.95	1.50	29.62	2.90	32.29	1.63	26.42	3.01
GE41042	NM_025979.2	microtubule associated serine/threonine kinase-like	Mastl	0.17	0.02	0.27	0.04	0.17	0.09	0.16	0.03	0.18	0.03	0.01	0.07
GE122917	AK048193.1	microtubule associated serine/threonine kinase 2	Mast2	2.31	0.14	2.67	0.35	2.55	0.23	3.06	0.66	3.52	0.46	2.46	0.38
GE131655	NM_145515.1	MAP/microtubule affinity-regulating kinase 1	Mark1	0.51	0.06	0.46	0.13	0.48	0.04	0.36	0.11	0.41	0.08	0.35	0.12
GE38216	NM_021516.1	MAP/microtubule affinity-regulating kinase 3	Mark3	22.07	0.95	20.24	0.96	18.01	0.71	20.55	1.28	24.96	1.44	21.39	1.82
GE40009	AY252101.1	testicular microtubules-related protein 3 mRNA	Tekt3	0.12	0.03	0.09	0.04	0.08	0.02	0.08	0.01	0.10	0.02	0.10	0.04
GE1484683	NM_133350.1	microtubule-associated protein, RP/EB family, member 3	Mapre3	4.35	0.44	4.80	0.21	3.49	0.72	4.54	0.77	5.77	0.32	4.48	0.77
GE1409648	AK084594.1	hook microtubule tethering protein 3	Hook3	6.15	0.11	6.76	0.26	5.69	0.35	3.82	0.91	4.99	0.83	4.69	0.81
GE34055	NM_008641.1	microtubule associated serine/threonine kinase 2	Mast2	5.91	0.36	6.30	0.37	5.94	0.51	6.24	1.35	8.52	0.50	6.99	1.67
GE42488	NM_007928.1	MAP/microtubule affinity-regulating kinase 2	Mark2	2.94	0.51	3.80	0.07	2.86	0.35	2.69	0.47	4.42	0.50	3.17	1.01
GE35766	NM_010837.1	microtubule-associated protein 6	Mtap6	0.42	0.01	0.53	0.05	0.31	0.18	0.51	0.16	0.86	0.05	0.41	0.02
GE136755	BE629164.1	Microtubule associated monoxygenase, calponin and LIM domain containing 3	Mical3	2.15	0.22	3.57	0.17	2.23	0.25	3.32	0.49	8.75	0.68	2.78	0.12

Supplementary-table 9. Cont'd….

Substance	ACCN#	Description	Gene Symbol	(Gene expression) 2 weeks						(Gene expression) 3 weeks					
				CTL		Hom-CKO		Het_CKO		CTL		Hom-CKO		Het_CKO	
				Average	SD	Average	SD	Average	SD	Average	SD	Average	SD	Average	SD
GE1509678	NM_175114.2	Trafficking protein, kinesin binding 1	Trak1	66.62	1.84	52.19	4.53	59.32	3.13	82.19	9.98	38.06	1.30	65.39	4.54
GE1395625	AK030188.1	kinesin family member 2B	Kif2b	0.15	0.01	0.08	0.05	0.06	0.03	0.13	0.05	0.07	0.04	0.04	0.04
GE35649	NM_010628.1	kinesin family member 9	Kif9	0.26	0.09	0.23	0.07	0.19	0.06	0.21	0.11	0.15	0.04	0.23	0.15
GE35651	NM_010630.1	kinesin family member C2	Kifc2	1.28	0.32	1.28	0.10	1.49	0.07	2.46	0.49	1.85	0.11	1.97	0.48
GE36981	NM_016705.1	kinesin family member 21A	Kif21a	2.24	0.17	2.08	0.25	2.24	0.11	2.82	0.64	2.35	0.30	2.59	0.46
GE136547	NM_153103.1	kinesin family member 1C	Kif1c	11.79	0.78	10.80	0.54	10.84	0.19	15.73	0.60	13.55	0.48	16.01	1.01
GE40597	NM_024245.3	kinesin family member 23	Kif23	0.54	0.12	0.59	0.12	0.50	0.05	0.16	0.10	0.15	0.16	0.25	0.19
GE1416173	AK033819.1	Rho GTPase activating protein 20, opposite strand	Arhgap20os	0.04	0.05	0.04	0.04	0.03	0.02	0.08	0.07	0.08	0.04	0.13	0.08
GE34692	NM_008446.1	kinesin family member 4	Kif4	0.87	0.19	0.92	0.14	0.70	0.12	0.40	0.30	0.36	0.19	0.30	0.20
GE42530	NM_008451.1	kinesin light chain 2	Klc2	14.38	0.94	14.46	0.75	13.79	0.85	17.65	3.16	16.39	2.31	16.64	2.99
GE34693	NM_008447.2	kinesin family member 5A	Kif5a	0.09	0.07	0.10	0.08	0.00	0.04	0.08	0.03	0.08	0.02	0.11	0.02
GE1390499	NM_008449.2	kinesin family member 5C	Kif5c	8.08	0.47	7.70	0.57	7.19	0.64	8.23	1.37	7.78	0.59	6.90	1.10
GE1554163	NM_010615.1	kinesin family member 11	Kif11	2.22	0.23	2.53	0.31	1.86	0.09	1.85	0.59	1.76	0.38	1.80	0.24
GE37675	NM_019962.2	kinesin family member 21B	Kif21b	1.77	0.05	1.83	0.15	2.06	0.24	1.91	0.33	1.84	0.21	1.88	0.11
GE42529	NM_008450.1	kinesin 2	Kns2	27.44	2.67	26.09	2.83	24.92	1.72	30.82	3.74	29.70	1.62	28.26	3.96
GE133661	NM_146182.1	kinesin light chain 3	Klc3	0.42	0.07	0.48	0.08	0.46	0.07	0.45	0.04	0.43	0.14	0.45	0.03
GE34691	NM_008444.2	kinesin family member 3B	Kif3b	12.42	1.40	12.96	0.96	12.80	0.75	12.15	1.30	11.91	0.55	10.96	0.29
GE38239	NM_010631.1	kinesin family member C3	Kifc3	9.60	1.16	9.83	0.35	9.74	0.34	12.05	2.12	12.19	1.04	11.31	2.59
GE1550906	AK029721.1	kinesin family member 13A	Kif13a	2.17	0.53	1.92	0.36	2.12	0.03	2.39	0.57	2.44	0.47	2.17	0.33
GE1454574	NM_177052.2	kinesin family member 6	Kif6	0.89	0.12	0.79	0.01	0.93	0.03	0.97	0.10	0.99	0.13	0.94	0.13
GE1554592	NM_019962.2	kinesin family member 21B	Kif21b	8.92	0.31	9.55	0.35	8.92	0.33	9.55	0.56	9.76	0.10	8.10	0.64
GE1422468	NM_008441.1	kinesin family member 1B, transcript variant 1	Kif1b	31.37	6.21	33.53	1.12	31.55	1.23	30.48	6.04	31.27	1.53	29.09	6.08
GE35650	NM_010629.1	kinesin-associated protein 3	Kifap3	5.75	0.35	6.13	0.45	5.39	0.10	4.86	0.27	5.09	0.65	3.91	0.31
GE41486	NM_010623.2	kinesin family member 17	Kif17	0.01	0.04	0.05	0.02	0.08	0.00	0.12	0.08	0.13	0.05	0.08	0.08
GE132039	NM_145588.1	kinesin family member 22	Kif22	3.87	0.85	4.62	0.57	3.49	0.62	1.65	0.44	1.75	0.74	1.50	0.65
GE1570569	NM_010616.1	kinesin family member 12	Kif12	2.32	0.16	3.24	0.49	5.15	0.44	3.07	0.33	3.61	0.23	4.06	0.38
GE1512445	AV255430.1	RIKEN adult male testis (DH10B) cDNA clone 4921520B19 3' similar to U44979 Rattus norvegicus kinesin-related protein 2 mRNA	EST	0.11	0.08	0.03	0.01	0.06	0.01	0.07	0.07	0.08	0.06	0.13	0.01
GE102253	NM_134471.2	kinesin family member 2C	Kif2c	1.38	0.10	1.78	0.07	1.67	0.17	1.10	0.12	1.47	0.33	1.47	0.08
GE34694	NM_008448.1	kinesin family member 5B	Kif5b	30.77	2.14	32.88	0.67	27.63	0.36	24.53	5.41	33.11	2.04	23.82	1.37
GE42528	NM_008445.1	kinesin family member 3C	Kif3c	0.88	0.18	1.26	0.12	0.95	0.07	0.94	0.06	1.30	0.11	0.87	0.11
GE129460	NM_139303.1	kinesin family member 18A	Kif18a	1.28	0.37	1.27	0.13	1.16	0.20	0.74	0.22	1.03	0.10	0.94	0.19
GE33326	NM_010615.1	kinesin family member 11	Kif11	1.35	0.20	1.61	0.17	1.20	0.03	0.39	0.21	0.58	0.15	0.59	0.21
GE34316	NM_009004.2	kinesin family member 20A	Kif20a	0.26	0.07	0.33	0.12	0.22	0.11	0.13	0.04	0.23	0.08	0.18	0.10
GE38492	NM_175138.2	dynein, axonemal, intermediate chain 1	Dnaic1	1.82	0.27	1.35	0.06	1.84	0.19	2.66	0.46	1.77	0.09	1.84	0.09
GE37188	NM_017470.1	dynein, axonemal, light chain 4	Dnalc4	3.04	0.12	2.65	0.04	3.12	0.14	4.21	0.37	2.98	0.37	3.43	0.36
GE1522945	AK039057.1	dynein, axonemal, heavy chain 9	Dnah9	0.15	0.07	0.16	0.04	0.10	0.04	0.18	0.08	0.13	0.04	0.19	0.03
GE35316	NM_010064.1	dynein, cytoplasmic, intermediate chain 2	Dncic2	21.66	0.81	20.95	0.18	17.59	0.29	23.32	1.35	18.63	0.59	17.05	0.97
GE39243	NM_025947.1	dynein, cytoplasmic, light chain 2A	Dncl2a	73.43	10.49	61.56	3.82	56.29	0.76	100.81	18.40	84.99	3.02	75.03	7.52
GE39854	AK014500.1	dynein cytoplasmic 2 heavy chain 1	Dync2h1	1.32	0.07	1.09	0.10	1.08	0.18	0.86	0.22	0.73	0.05	0.92	0.09
GE35315	NM_010063.1	dynein, cytoplasmic, intermediate chain 1	Dncic1	0.14	0.02	0.13	0.04	0.16	0.03	0.09	0.05	0.08	0.04	0.14	0.08
GE38483	AK004235.1	dynein cytoplasmic 1 light intermediate chain 1	Dync1li1	17.70	2.05	17.39	2.04	16.89	0.36	21.41	4.72	19.25	0.67	17.72	0.81
GE35313	NM_010060.1	dynein, axonemal, heavy chain 11	Dnahc11	0.11	0.01	0.08	0.04	0.10	0.01	0.09	0.14	0.08	0.05	0.12	0.02
GE1528713	AK081229.1	dynein, axonemal, intermediate chain 2	Dnaic2	3.20	0.30	2.98	0.07	3.21	0.17	3.44	0.14	3.15	0.15	4.08	0.12
GE1509015	AK047928.1	dynein cytoplasmic 2 heavy chain 1	Dync2h1	0.20	0.13	0.35	0.05	0.30	0.06	0.22	0.14	0.20	0.04	0.21	0.09
GE1439006	NM_146229.1	dynein, cytoplasmic, light intermediate chain 1	Dnclic1	0.84	0.01	0.78	0.16	0.81	0.08	1.07	0.22	1.09	0.25	0.89	0.04
GE1552109	Z83815.2	Mmusculus mRNA for axonemal dynein heavy chain (partial, ID mdhc7)	Dnah1	0.10	0.03	0.07	0.06	0.08	0.01	0.09	0.07	0.10	0.04	0.15	0.08
GE1543165	AK034892.1	centrosomal protein 72	Cep72	2.34	0.14	2.88	0.21	3.17	0.20	2.85	0.05	3.07	0.06	3.22	0.03
GE40705	NM_030238.1	dynein, cytoplasmic, heavy chain 1	Dnchc1	13.53	2.58	13.72	0.87	13.55	0.73	11.75	0.74	13.56	1.30	11.84	1.79
GE1453519	AA673709.1	dynein, axonemal, heavy chain 7A	Dnah7a	0.16	0.05	0.03	0.00	0.10	0.04	0.08	0.03	0.11	0.04	0.08	0.05
GE38806	NM_029297.1	dynein, cytoplasmic, light chain 2B	Dncl2b	0.14	0.05	0.21	0.05	0.20	0.03	0.13	0.12	0.18	0.06	0.20	0.05
GE126915	AK034139.1	dynein, axonemal, heavy chain 5	Dnah5	0.20	0.08	0.32	0.06	0.26	0.06	0.17	0.15	0.34	0.10	0.27	0.16
GE125652	NM_030727.3	prestin (motor protein)	Pres	0.14	0.07	0.10	0.04	0.19	0.07	0.26	0.12	0.17	0.12	0.18	0.05

Supplementary table 9. Cont'd….

Substance	ACCN#	Description	Gene Symbol	(Gene expression) 2 weeks						(Gene expression) 3 weeks					
				CTL		Hom-CKO		Het_CKO		CTL		Hom-CKO		Het_CKO	
				Average	SD	Average	SD	Average	SD	Average	SD	Average	SD	Average	SD
GE1474761	AA655504.1	vs25c09r1 Barstead mouse myotubes MPLRB5 cDNA clone IMAGE:1139248 5' similar to gb:K02241_cds1 Mouse alkali myosin light chains (MOUSE);.	EST	0.08	0.09	0.10	0.02	0.15	0.03	0.16	0.07	-0.02	0.02	0.10	0.03
GE40604	NM_024427.2	tropomyosin 1, alpha	Tpm1	162.52	27.23	91.66	20.20	130.19	14.73	204.48	35.46	86.06	9.38	170.24	13.60
GE38227	NM_022879.1	myosin, light polypeptide 7, regulatory	Myl7	37.41	9.13	23.55	10.42	23.57	38.39	25.47	33.73	10.73	7.11	25.86	5.49
GE130925	AK033571.1	Myosin VC	Myo5c	2.60	0.44	1.51	0.11	2.92	0.17	2.09	0.50	0.89	0.10	2.72	0.63
GE1567587	BC030481.1	myosin Vb, mRNA, with apparent retained intron	Myo5b	0.16	0.16	0.08	0.06	0.11	0.06	0.22	0.07	0.10	0.01	0.17	0.00
GE38396	NM_021285.1	myosin, light polypeptide 1	Myl1	0.47	0.08	0.32	0.09	0.59	0.07	0.61	0.05	0.29	0.16	0.47	0.10
GE35775	NM_010858.3	myosin, light polypeptide 4	Myl4	36.43	10.52	27.26	3.78	28.00	11.34	38.61	14.12	21.00	4.19	32.27	0.49
GE1483970	AK017709.1	filamin A interacting protein 1	Filip1	1.85	0.07	1.19	0.10	1.79	0.13	1.98	0.32	1.11	0.11	1.73	0.20
GE119117	NM_010861.1	myosin, light polypeptide 2, regulatory, cardiac, slow	Myl2	475.16	80.76	332.41	29.41	341.49	16.16	640.13	61.35	389.69	22.98	483.97	49.77
GE109000	AV298897.1	RIKEN 8 days embryo cDNA clone 5730462N15 3' similar to K02242 Mouse alkali myosin light chains MLC1f and MLC3f 3' mRNA, clone p161	EST	0.05	0.07	0.08	0.03	0.03	0.04	0.17	0.01	0.11	0.07	0.08	0.13
GE32913	X67685.1	Mmusculus mRNA for ventricular alkali myosin light chain	Myl3	229.60	40.31	174.33	25.03	176.15	8.04	283.11	38.18	180.22	11.65	236.19	8.53
GE33337	NM_144961.1	myosin, heavy polypeptide 2, skeletal muscle, adult	Myh2	0.26	0.08	0.29	0.04	0.38	0.04	0.32	0.07	0.21	0.05	0.43	0.04
GE117269	M12289.1	perinatal skeletal myosin heavy chain mRNA, 3' end	Not available	1.40	0.26	1.35	0.02	1.61	0.04	3.46	1.10	2.39	0.23	3.45	0.89
GE1473366	BC023701.1	tropomyosin 4	Tpm4	73.55	17.28	70.10	5.12	64.92	1.17	70.80	11.47	50.82	3.46	62.98	15.60
GE129726	NM_144557.2	myosin VIIA and Rab interacting protein	Myrip	0.14	0.03	0.19	0.07	0.19	0.09	0.16	0.17	0.12	0.03	0.20	0.06
GE1567384	NM_010859.1	myosin, light polypeptide 3	Myl3	502.89	30.54	417.80	49.01	399.11	6.20	600.36	27.61	438.01	4.59	495.28	34.81
GE42738	NM_016754.3	myosin light chain, phosphorylatable, fast skeletal muscle	Mylpf	0.46	0.21	0.53	0.04	0.52	0.04	0.71	0.16	0.53	0.02	0.64	0.10
GE120785	NM_022410.1	myosin heavy chain IX	Myh9	73.24	3.02	74.71	3.98	65.14	6.94	72.78	13.37	53.73	3.31	55.92	7.16
GE34069	NM_008663.1	myosin VIIa	Myo7a	0.61	0.06	0.50	0.04	0.50	0.03	0.88	0.17	0.65	0.11	0.57	0.18
GE35777	NM_010863.1	myosin IB	Myo1b	7.56	0.59	7.92	0.63	7.24	0.21	5.29	1.22	3.96	0.14	4.68	0.30
GE38363	NM_023402.1	myosin light chain, regulatory B	Mylc2b	18.67	4.14	16.89	1.94	16.94	1.02	19.67	5.53	14.72	0.34	15.86	3.21
GE110488	AV379817.1	myosin, heavy polypeptide 13, skeletal muscle	Myh13	0.12	0.12	0.12	0.04	0.16	0.09	0.14	0.06	0.11	0.08	0.16	0.07
GE33070	AA619330.1	vo83e06r1 Barstead mouse myotubes MPLRB5 cDNA clone IMAGE:1065730 5' similar to gb:U04443 Mus musculus non-muscle myosin light chain 3 (MOUSE);.	EST	82.88	22.10	76.02	7.52	65.18	4.10	96.20	15.85	75.72	8.45	83.75	16.86
GE41650	AA882433.1	vx44g03r1 Stratagene mouse lung 937302 cDNA clone IMAGE:1278100 5' similar to gb:X53753 Mouse mRNA for nonmuscle tropomyosin 5 (MOUSE);.	EST	19.95	0.44	18.93	0.83	16.33	0.78	20.45	4.04	16.44	1.75	16.70	2.47
GE1498071	W40629.1	mc49f05r1 Soares mouse p3NMF19.5 cDNA clone IMAGE:351873 5' similar to gb:M36769 MYOSIN HEAVY CHAIN, PERINATAL SKELETAL MUSCLE (HUMAN); gb:M76601 Mouse alpha cardiac myosin heavy chain mRNA, complete (MOUSE);.	EST	0.20	0.02	0.19	0.05	0.28	0.05	0.22	0.11	0.18	0.09	0.28	0.10
GE35774	NM_010856.2	myosin, heavy polypeptide 6, cardiac muscle, alpha	Myh6	3.78	1.30	4.26	0.23	5.35	0.50	4.91	1.31	4.27	0.94	5.15	1.17
GE136335	NM_148413.1	myosin IIIA	Myo3a	0.10	0.08	0.11	0.04	0.06	0.03	0.10	0.09	0.09	0.05	0.08	0.03
GE137117	NM_153789.2	myosin regulatory light chain interacting protein	Mylip	9.56	1.34	7.15	0.80	7.77	0.29	7.72	1.00	6.80	0.37	5.88	0.78
GE1431137	XM_359049.1	similar to myosin heavy chain (LOC386062)	Not available	0.06	0.04	0.08	0.04	0.07	0.03	0.09	0.01	0.08	0.07	0.03	0.06
GE1506439	AJ293626.1	partial mRNA for myosin heavy chain IIX (MYHC-IIX gene)	Myh1	0.12	0.08	0.11	0.06	0.04	0.04	0.16	0.09	0.14	0.02	0.10	0.01
GE37442	NM_019584.2	beclin 1 (coiled-coil, myosin-like BCL2-interacting protein)	Becn1	11.66	1.65	11.69	0.59	10.30	0.53	12.58	0.69	11.27	1.24	9.89	0.55
GE42536	NM_008653.1	myosin binding protein C, cardiac	Mybpc3	137.57	15.74	139.01	2.44	123.84	5.86	161.22	15.11	147.40	9.81	134.85	12.62
GE37047	NM_016749.1	myosin binding protein H	Mybph	0.09	0.05	0.25	0.03	0.09	0.02	0.20	0.15	0.18	0.07	0.13	0.04
GE41741	NM_032394.1	myosin VIIb	Myo7b	0.92	0.15	1.02	0.09	1.17	0.24	1.03	0.11	0.97	0.03	1.05	0.10
GE36841	NM_011586.1	myosin XVIIIa	Myo18a	9.06	1.60	8.62	0.07	8.79	0.54	8.26	0.51	7.85	0.55	7.27	1.10
GE32958	AJ278733.1	myosin, heavy polypeptide 4, skeletal muscle	Myh4	0.12	0.07	0.12	0.01	0.02	0.01	0.06	0.03	0.06	0.05	0.08	0.05
GE1437572	BC046444.1	myosin VB	Myo5b	0.29	0.08	0.43	0.03	0.63	0.08	0.55	0.05	0.54	0.05	0.56	0.06
GE1576101	AA048060.1	mj28d02r1 Soares mouse embryo NbME13.5 14.5 cDNA clone IMAGE:477411 5' similar to gb:X53753 Mouse mRNA for nonmuscle tropomyosin 5 (MOUSE);.	EST	1.25	0.05	1.15	0.08	1.22	0.03	1.24	0.13	1.21	0.10	1.28	0.07
GE111603	NM_053214.1	myosin IF	Myo1f	2.04	0.69	1.71	0.11	1.87	0.12	2.89	0.79	2.83	0.26	2.39	0.72
GE35778	NM_010864.1	myosin Va	Myo5a	0.60	0.11	0.65	0.01	0.52	0.01	0.69	0.27	0.72	0.06	0.62	0.07
GE111857	AW907483.1	us16b11.x1 Soares_NMGBC_B-cell cDNA clone IMAGE:3167229 3' similar to gb:X65979 M.musculus PLRLC-A mRNA for myosin light chain 2 (MOUSE);	EST	1.61	0.21	1.69	0.22	1.89	0.06	1.79	0.27	1.87	0.09	1.92	0.31

Supplementary table 9. Cont'd….

Substance	ACCN#	Description	Gene Symbol	(Gene expression) 2 weeks						(Gene expression) 3 weeks					
				CTL		Hom-CKO		Het_CKO		CTL		Hom-CKO		Het_CKO	
				Average	SD	Average	SD	Average	SD	Average	SD	Average	SD	Average	SD
GE117299	M74753.1	myosin heavy chain mRNA, 3' flank	Myh3	0.08	0.04	0.17	0.06	0.15	0.05	0.22	0.03	0.23	0.11	0.16	0.04
GE1572942	NM_008662.1	myosin VI	Myo6	3.64	0.13	3.83	0.53	3.57	0.35	2.77	0.70	2.97	0.15	2.56	0.24
GE40236	BC040786.1	myosin IE	Myo1e	1.40	0.31	1.71	0.04	1.58	0.10	1.53	0.15	1.67	0.08	1.15	0.09
GE1574436	AK004148.1	myosin binding protein H-like	Mybphl	1.85	0.49	2.71	0.95	1.78	2.77	1.86	2.47	2.04	1.05	1.61	0.74
GE38574	AK004785.1	protein phosphatase 1, regulatory (inhibitor) subunit 12A	Ppp1r12a	4.55	0.83	5.11	0.13	4.38	0.17	4.03	0.51	4.44	0.33	4.10	0.98
GE37397	NM_019472.1	myosin X	Myo10	5.05	0.93	4.75	0.24	4.36	0.07	4.05	0.29	4.46	0.40	3.70	0.87
GE1541508	AK034257.1	myosin XVI	Myo16	0.07	0.05	0.06	0.03	0.05	0.07	0.10	0.01	0.11	0.04	0.06	0.01
GE39975	AK016515.1	myosin XVIIIb	Myo18b	3.61	0.18	3.97	0.29	4.31	0.30	3.89	0.43	4.40	0.39	3.51	0.17
GE39967	AK016375.1	family with sequence similarity 184, member A	Fam184a	0.16	0.09	0.18	0.14	0.15	0.03	0.16	0.10	0.18	0.04	0.11	0.05
GE40857	NM_181072.2	myosin IE	Myo1e	0.55	0.28	0.88	0.08	0.75	0.03	0.48	0.25	0.57	0.06	0.44	0.10
GE34067	NM_008659.2	myosin IC	Myo1c	7.57	1.18	8.02	0.44	7.43	0.14	7.35	0.85	8.72	0.81	7.84	2.63
GE36698	NM_013607.1	myosin heavy chain 11, smooth muscle	Myh11	5.83	2.33	5.42	0.94	6.16	0.67	3.03	0.26	3.65	0.30	3.64	1.16
GE1548796	NM_178440.2	myosin IG	Myo1g	1.57	0.35	1.30	0.11	1.34	0.06	2.19	0.24	2.65	0.33	1.82	0.44
GE108930	AV293917.1	RIKEN 10 days neonate head cDNA clone 5530401A04 3' similar to AJ002522 Mus musculus mRNA for myosin heavy chain 2X	EST	0.13	0.08	0.15	0.05	0.08	0.05	0.05	0.07	0.06	0.04	0.10	0.06
GE35776	NM_010862.1	myosin XV, transcript variant 1	Myo15	0.86	0.07	0.98	0.05	1.09	0.02	0.83	0.04	1.02	0.02	1.04	0.08
GE1422824	AF336977.1	extraocular myosin heavy chain EO-MYHC (Eomyhc) mRNA	Myh13	0.18	0.06	0.28	0.16	0.41	0.01	0.26	0.06	0.33	0.15	0.29	0.02
GE138465	AK029836.1	myosin IXa	Myo9a	0.96	0.20	1.02	0.07	1.03	0.08	0.92	0.15	1.16	0.07	0.92	0.04
GE38063	NM_021611.1	myosin light chain 2, precursor lymphocyte-specific	Mylc2pl	0.06	0.08	0.05	0.06	0.09	0.04	0.07	0.02	0.09	0.07	0.11	0.05
GE133585	NM_146163.2	myosin 1H	Myo1h	0.17	0.10	0.25	0.07	0.21	0.03	0.19	0.11	0.24	0.14	0.20	0.02
GE1526239	BC049974.1	myosin, light polypeptide 9, regulatory, mRNA	Myl9	39.03	1.64	54.36	6.31	39.44	4.73	32.97	2.37	44.54	4.99	30.62	2.04
GE112680	BB023791.1	RIKEN adult male pituitary gland cDNA clone 5330416O10 3' similar to AJ223362 Mus musculus mRNA for slow myosin heavy chain-beta, partial	EST	0.08	0.02	0.02	0.03	0.05	0.03	0.10	0.03	0.13	0.04	0.13	0.07
GE39239	AF009960.1	brush border myosin-I mRNA	BBM-I	0.39	0.16	0.50	0.06	0.45	0.11	0.24	0.05	0.34	0.07	0.24	0.04
GE38164	NM_009416.2	tropomyosin 2, beta	Tpm2	3.35	0.18	4.34	0.08	3.39	0.25	3.52	0.55	5.20	0.51	3.24	0.34
GE40303	NM_175260.1	myosin heavy chain 10, non-muscle	Myh10	1.53	0.10	1.49	0.21	1.20	0.07	0.96	0.17	1.63	0.17	1.12	0.14
GE36906	NM_015742.1	myosin IXb	Myo9b	6.76	0.27	7.91	0.73	6.39	0.69	5.44	1.03	9.57	0.76	6.02	0.51
GE121329	NM_025414.2	myosin head domain containing 1	Myohd1	2.81	0.29	3.38	0.08	2.54	0.09	2.35	0.64	4.38	0.26	2.63	0.13
GE1424158	NM_146189.1	myosin binding protein C, fast-type	Mybpc2	12.78	0.47	14.77	1.44	13.66	1.80	13.91	5.03	30.31	0.88	12.51	2.46
GE1394211	AK079396.1	Myosin light polypeptide kinase 2, skeletal muscle	Mylk2	0.23	0.04	0.40	0.07	0.22	0.04	0.26	0.01	2.01	0.22	0.12	0.04
GE112311	NM_138579.2	TRIO and F-actin binding protein	Triobp	20.70	0.49	19.13	0.55	18.57	0.88	20.52	2.34	16.94	1.30	14.79	2.54

Supplementary table 10. List of hand-curated "Cell-death pathway" genes used for Fisher's Exact test

				(Gene expression) 2 weeks						(Gene expression) 3 weeks					
Substance	ACCN#	Description	Gene Symbol	CTL		Hom-CKO		Het-CKO		CTL		Hom-CKO		Het-CKO	
				Average	SD	Average	SD	Average	SD	Average	SD	Average	SD	Average	SD
GE34173	NM_008799.1	programmed cell death 2	pdcd2	16.49	2.85	14.66	0.98	14.20	0.87	20.42	3.02	16.42	0.78	16.64	1.89
GE36969	NM_016688.1	programmed cell death protein 7	pdcd7	1.68	0.14	1.59	0.15	1.56	0.13	1.76	0.17	1.46	0.06	1.81	0.09
GE35866	NM_011051.2	programmed cell death 6	pdcd6	36.33	9.33	34.09	1.44	30.23	1.05	35.63	5.19	31.91	0.48	29.96	4.82
GE1565628	NM_008798.1	programmed cell death 1	Pdcd1	0.79	0.24	0.86	0.08	0.92	0.16	0.93	0.03	0.84	0.04	0.93	0.07
GE38457	BC038503.1	programmed cell death 11	pdcd11	3.15	0.54	3.68	1.05	3.02	0.35	3.36	0.08	3.16	0.35	2.95	0.37
GE35867	NM_011052.1	programmed cell death 6 interacting protein	pdcd6ip	1.87	0.55	2.23	0.42	1.63	0.33	2.49	0.90	2.39	0.16	1.94	0.54
GE118757	NM_010015.1	defender against cell death 1	dad1	31.99	4.08	29.12	1.21	29.52	0.79	38.56	2.29	37.60	0.17	34.09	2.44
GE1456353	AK035678.1	predicted gene, 36043	gm36043	0.48	0.03	0.48	0.05	0.59	0.03	0.43	0.10	0.45	0.09	0.56	0.14
GE35865	NM_011050.1	programmed cell death 4	pdcd4	2.92	0.61	3.31	0.50	3.01	0.12	3.04	0.11	3.72	0.27	2.64	0.08
GE42705	NM_012019.2	programmed cell death 8	pdcd8	20.11	1.62	17.29	2.07	18.82	0.22	23.12	4.48	16.03	0.27	20.13	1.53
GE38098	NM_021893.2	programmed cell death 1 ligand 1	pdcd1lg1	0.99	0.21	0.99	0.10	1.03	0.20	1.66	0.19	1.18	0.05	1.33	0.13
GE1517980	NM_021893.2	programmed cell death 1 ligand 1	pdcd1lg1	2.50	0.42	2.41	0.31	2.63	0.33	3.82	0.85	2.78	0.17	2.80	0.57
GE120632	NM_021396.1	programmed cell death 1 ligand 2	pdcd1lg2	3.96	0.41	3.37	0.08	3.56	0.26	3.65	0.42	2.82	0.18	4.39	0.21
GE34856	NM_007702.1	cell death-inducing DNA fragmentation factor, alpha subunit-like effector A	cidea	18.26	0.88	17.39	0.87	16.89	0.52	11.42	3.40	15.75	0.90	10.62	1.29
GE122035	AK046479.1	apoptosis, caspase activation inhibitor	aven	0.08	0.10	0.19	0.09	0.12	0.04	0.02	0.09	0.19	0.03	0.15	0.04
GE41345	AK003968.1	cell death-inducing DNA fragmentation factor, alpha subunit-like effector B	cideb	0.77	0.05	0.62	0.02	0.67	0.01	1.08	0.28	0.68	0.01	0.91	0.08
GE38155	NM_022323.3	modulator of apoptosis 1	moap1	8.92	0.55	8.21	0.41	7.77	0.69	10.63	1.32	8.44	0.31	8.87	0.86
GE42299	BE628951.1	tumor necrosis factor (ligand) superfamily, member 12	tnfsf12	2.27	0.38	2.34	0.28	2.44	0.19	2.80	0.17	2.42	0.05	2.34	0.36
GE102349	NM_025976.1	bifunctional apoptosis regulator	bfar	18.01	0.39	15.40	1.49	15.30	0.98	18.52	2.39	16.10	1.28	18.70	4.13
GE40080	NM_153779.1	apoptosis-inducing factor (AIF)-like mitochondrion-associated inducer of death	amid	3.06	0.39	3.68	0.28	2.80	0.05	4.09	0.61	3.57	0.20	3.31	0.54
GE39789	NM_025976.1	bifunctional apoptosis regulator	bfar	16.79	1.45	16.26	1.24	16.77	1.81	18.62	3.31	16.32	0.32	16.04	1.29
GE136866	NM_153516.1	BCL2-like 13 (apoptosis facilitator)	bcl2l13	6.48	0.68	6.53	0.79	6.76	0.49	7.69	1.17	6.97	0.40	6.46	0.66
GE1403671	NM_199042.1	THAP domain containing, apoptosis associated protein 1	thap1	1.24	0.18	1.32	0.08	1.15	0.12	1.54	0.15	1.44	0.08	1.16	0.05
GE37519	NM_019816.1	apoptosis antagonizing transcription factor	aatf	0.48	0.06	0.39	0.09	0.43	0.05	0.48	0.25	0.45	0.09	0.58	0.03
GE127337	NM_133810.2	serine/threonine kinase 17b (apoptosis-inducing)	stk17b	3.50	0.52	3.14	0.12	3.01	0.15	2.97	0.38	2.84	0.23	3.14	0.18
GE107600	NM_025778.1	Bcl2-like 14 (apoptosis facilitator)	bcl2l14	1.37	0.10	1.50	0.19	1.68	0.22	1.41	0.16	1.35	0.11	1.42	0.24
GE33470	NM_007609.1	caspase 4, apoptosis-related cysteine protease	casp4	1.08	0.59	0.73	0.00	0.75	0.05	1.25	0.29	1.24	0.14	1.19	0.29
GE35157	NM_009810.1	caspase 3, apoptosis related cysteine protease	casp3	2.25	0.68	2.18	0.12	1.90	0.13	1.60	0.41	1.63	0.16	1.48	0.30
GE124534	NM_028844.1	apoptosis, caspase activation inhibitor	aven	4.02	0.64	4.19	0.15	3.81	0.40	4.57	0.28	4.71	0.12	4.24	0.15
GE103585	AK007714.1	Fc fragment of IgM receptor	fcmr	0.33	0.27	0.44	0.03	0.46	0.07	0.50	0.16	0.56	0.05	0.45	0.02
GE41287	NM_026531.2	apoptosis enhancing nuclease , mRNA	aen	2.87	0.41	4.98	0.23	3.24	0.32	5.01	1.87	8.64	1.44	3.93	0.33
GE33454	NM_007545.1	BH3 interacting (with BCL2 family) domain, apoptosis agonist	bid3	0.32	0.00	0.32	0.06	0.31	0.11	0.15	0.14	0.32	0.04	0.29	0.08
GE40413	NM_023258.3	apoptosis-associated speck-like protein containing a CARD	asc	4.16	1.48	2.26	0.14	3.03	0.12	5.79	2.24	2.66	0.40	5.53	1.25
GE1565718	NM_007377.1	apoptosis-associated tyrosine kinase	aatk	0.19	0.05	0.12	0.08	0.16	0.11	0.20	0.06	0.13	0.03	0.11	0.12
GE38143	NM_022032.1	PERP, TP53 apoptosis effector	perp	22.68	3.33	15.73	0.97	20.85	1.61	27.32	0.76	19.12	0.57	24.33	3.18
GE35120	NM_009754.2	BCL2-like 11 (apoptosis facilitator), transcript variant 3	bcl2l11	1.47	0.28	1.23	0.21	1.63	0.08	1.45	0.35	1.05	0.20	1.26	0.06
GE1513430	NM_134131.1	tumor necrosis factor, alpha-induced protein 8	tnfaip8	1.75	0.58	0.76	0.15	1.30	0.10	1.96	0.20	0.66	0.11	2.03	0.28
GE1414048	NM_175425.2	C1q and tumor necrosis factor related protein 7	c1qtnf7	5.66	2.14	3.52	0.13	3.85	0.20	5.87	1.46	3.42	0.25	4.74	1.24
GE1543381	NM_175649.2	tumor necrosis factor receptor superfamily, member 26	tnfrsf26	0.30	0.10	0.27	0.08	0.35	0.06	0.34	0.07	0.21	0.05	0.14	0.05
GE42868	NM_026161.1	C1q and tumor necrosis factor related protein 4	c1qtnf4	3.33	0.71	1.83	0.09	2.91	0.25	3.70	0.46	2.43	0.27	4.12	0.53
GE41978	NM_178589.2	tumor necrosis factor receptor superfamily, member 21	tnfrsf21	0.76	0.11	0.77	0.02	0.73	0.02	1.02	0.18	0.67	0.07	0.75	0.09
GE38882	NM_134138.1	tumor necrosis factor superfamily, member 5-induced protein 1	tnfsf5ip1	12.68	1.80	10.90	0.49	11.18	0.65	16.30	2.62	10.92	0.28	13.17	1.31
GE40456	NM_023517.1	tumor necrosis factor (ligand) superfamily, member 13	tnfsf13	3.29	0.36	2.88	0.23	3.23	0.35	3.70	0.68	2.56	0.18	2.76	0.23
GE36222	NM_011617.1	tumor necrosis factor (ligand) superfamily, member 7	tnfsf7	0.18	0.06	0.15	0.07	0.18	0.03	0.27	0.02	0.19	0.08	0.20	0.12
GE34378	NM_007987.1	tumor necrosis factor receptor superfamily, member 6	tnfrsf6	0.11	0.01	0.07	0.07	0.08	0.08	0.14	0.01	0.10	0.06	0.12	0.04
GE41973	NM_028075.1	tumor necrosis factor receptor superfamily, member 13c	tnfrsf13c	0.80	0.04	0.98	0.14	1.22	0.11	1.18	0.18	0.86	0.06	1.09	0.18
GE37908	NM_021349.1	tumor necrosis factor receptor superfamily, member 13b	tnfrsf13b	0.62	0.11	0.56	0.01	0.57	0.06	0.91	0.09	0.67	0.09	0.76	0.10
GE40486	NM_023680.2	tumor necrosis factor receptor superfamily, member 22	tnfrsf22	0.65	0.11	0.60	0.07	0.60	0.01	0.73	0.04	0.55	0.07	0.53	0.08
GE1422504	NM_009399.2	tumor necrosis factor receptor superfamily, member 11a	tnfrsf11a	0.88	0.11	0.70	0.03	0.74	0.02	1.18	0.10	0.92	0.01	0.83	0.15
GE1421441	NM_011613.2	tumor necrosis factor (ligand) superfamily, member 11	tnfsf11	0.16	0.07	0.09	0.01	0.13	0.07	0.21	0.12	0.16	0.08	0.17	0.02
GE125781	NM_033042.2	tumor necrosis factor receptor superfamily, member 25	tnfrsf25	4.99	0.36	4.24	0.30	4.33	0.05	5.74	0.80	4.57	0.09	4.69	0.64
GE41772	NM_013869.3	tumor necrosis factor receptor superfamily, member 19	tnfrsf19	0.62	0.04	0.43	0.02	0.57	0.08	0.60	0.10	0.48	0.07	0.48	0.06
GE33829	NM_009404.1	tumor necrosis factor (ligand) superfamily, member 9	tnfsf9	0.92	0.11	0.96	0.02	0.94	0.04	1.09	0.22	0.88	0.08	1.03	0.02
GE41520	NM_030711.2	type 1 tumor necrosis factor receptor shedding aminopeptidase regulator	arts1	2.20	0.08	2.01	0.24	2.35	0.08	2.69	0.19	2.19	0.03	2.57	0.09
GE36219	NM_011611.1	tumor necrosis factor receptor superfamily, member 5	tnfrsf5	1.37	0.40	1.37	0.05	1.18	0.02	1.89	0.27	1.57	0.21	1.42	0.33
GE136309	NM_145613.2	C1q and tumor necrosis factor related protein 5	c1qtnf5	9.49	0.94	8.21	0.26	7.18	0.23	9.62	1.44	8.14	0.16	7.62	1.18

Supplementary table 10. Cont'd….

Substance	ACCN#	Description	Gene Symbol	(Gene expression) 2 weeks						(Gene expression) 3 weeks					
				CTL		Hom-CKO		Het-CKO		CTL		Hom-CKO		Het-CKO	
				Average	SD	Average	SD	Average	SD	Average	SD	Average	SD	Average	SD
GE36217	NM_011609.2	tumor necrosis factor receptor superfamily, member 1a	tnfrsf1a	7.68	0.02	7.77	0.18	7.31	0.81	9.81	0.59	8.36	0.38	7.29	0.50
GE42801	NM_019959.1	C1q and tumor necrosis factor related protein 1	c1qtnf1	4.52	0.22	4.26	0.32	4.36	0.40	4.20	0.41	3.65	0.18	3.30	0.09
GE33822	NM_009395.2	tumor necrosis factor, alpha-induced protein 1 (endothelial)	tnfaip1	7.44	0.38	7.99	0.86	6.97	0.36	5.78	0.90	5.24	0.11	5.26	0.08
GE33843	NM_009425.1	tumor necrosis factor (ligand) superfamily, member 10	tnfsf10	3.06	0.48	2.94	0.06	2.90	0.21	2.83	0.24	2.60	0.14	2.34	0.26
GE41817	NM_011616.2	tumor necrosis factor (ligand) superfamily, member 5	tnfsf5	0.60	0.10	0.69	0.13	0.76	0.08	0.85	0.06	0.79	0.03	0.77	0.07
GE33828	NM_009401.1	tumor necrosis factor receptor superfamily, member 8	tnfrsf8	2.04	0.36	2.11	0.23	1.94	0.16	2.27	0.13	2.13	0.11	2.15	0.05
GE1475439	AK054557.1	potassium channel tetramerisation domain containing 1	Kctd1	3.39	0.30	3.01	0.21	3.15	0.21	3.30	0.15	3.19	0.13	3.34	0.18
GE36218	NM_011610.1	tumor necrosis factor receptor superfamily, member 1b	tnfrsf1b	0.52	0.06	0.64	0.03	0.53	0.05	0.63	0.11	0.61	0.07	0.48	0.07
GE37434	NM_019418.1	tumor necrosis factor (ligand) superfamily, member 14	tnfsf14	1.12	0.23	1.05	0.21	0.94	0.03	0.89	0.19	0.87	0.18	0.86	0.12
GE34145	NM_008764.2	tumor necrosis factor receptor superfamily, member 11b (osteoprotegerin)	tnfrsf11b	3.00	0.19	2.99	0.14	2.99	0.10	3.55	0.52	3.53	0.17	3.58	0.57
GE119416	NM_011612.1	tumor necrosis factor receptor superfamily, member 9	tnfrsf9	5.08	0.38	5.01	0.38	4.71	0.71	4.00	0.66	4.00	0.31	4.02	0.33
GE33825	NM_009398.1	tumor necrosis factor alpha induced protein 6	tnfaip6	1.45	0.21	1.27	0.10	1.28	0.05	1.35	0.03	1.36	0.14	1.16	0.12
GE1392670	NM_011608.1	tumor necrosis factor receptor superfamily, member 17	tnfrsf17	0.40	0.07	0.26	0.06	0.35	0.05	0.35	0.08	0.36	0.10	0.31	0.07
GE42396	NM_028331.2	C1q and tumor necrosis factor related protein 6	c1qtnf6	9.73	0.92	8.94	0.06	8.92	0.79	8.65	0.92	9.00	0.46	8.13	0.43
GE41555	NM_030888.2	C1q and tumor necrosis factor related protein 3	c1qtnf3	0.38	0.15	0.49	0.09	0.47	0.04	0.33	0.08	0.35	0.16	0.42	0.11
GE36249	NM_011659.1	tumor necrosis factor receptor superfamily, member 4	tnfrsf4	0.53	0.13	0.55	0.07	0.42	0.09	0.51	0.12	0.54	0.01	0.43	0.11
GE35385	NM_010177.2	tumor necrosis factor (ligand) superfamily, member 6	tnfsf6	0.14	0.03	0.08	0.03	0.18	0.03	0.11	0.05	0.12	0.04	0.10	0.05
GE33823	NM_009396.1	tumor necrosis factor, alpha-induced protein 2	tnfaip2	13.54	1.14	14.57	1.77	15.01	0.97	13.06	3.09	14.21	8.20	12.17	1.27
GE33824	NM_009397.2	tumor necrosis factor, alpha-induced protein 3	tnfaip3	4.01	0.22	4.40	0.48	4.43	0.44	3.64	0.55	4.11	0.22	4.12	0.27
GE36806	NM_013693.1	tumor necrosis factor	tnf	0.16	0.07	0.33	0.04	0.27	0.07	0.29	0.05	0.33	0.16	0.29	0.01
GE1505825	NM_172541.1	transmembrane protein induced by tumor necrosis factor alpha	tmpit	2.10	0.39	2.17	0.25	1.85	0.16	3.06	0.19	3.55	0.23	2.50	0.58
GE38186	NM_020275.3	tumor necrosis factor receptor superfamily, member 10b	tnfrsf10b	2.68	0.11	2.78	0.18	3.52	0.27	3.04	0.66	3.53	0.27	4.13	0.21
GE41950	NM_033622.1	tumor necrosis factor (ligand) superfamily, member 13b	tnfsf13b	0.72	0.05	0.63	0.09	0.62	0.04	0.60	0.16	0.78	0.01	0.58	0.08
GE118534	NM_009452.1	tumor necrosis factor (ligand) superfamily, member 4	tnfsf4	0.04	0.03	0.09	0.02	0.08	0.02	0.11	0.04	0.17	0.03	0.10	0.04
GE1492107	NM_177073.3	tumor necrosis factor receptor superfamily, member 19-like	tnfrsf19l	0.58	0.09	0.83	0.09	0.74	0.12	0.55	0.13	0.91	0.10	0.58	0.06
GE40595	NM_024290.2	tumor necrosis factor receptor superfamily, member 23	tnfrsf23	0.54	0.07	0.64	0.03	0.52	0.04	0.69	0.11	1.19	0.15	0.53	0.05
GE102765	NM_009400.1	tumor necrosis factor receptor superfamily, member 18	tnfrsf18	0.27	0.10	0.27	0.07	0.16	0.07	0.24	0.08	0.70	0.10	0.19	0.11
GE33827	NM_009400.1	tumor necrosis factor receptor superfamily, member 18	tnfrsf18	0.23	0.02	0.26	0.04	0.18	0.05	0.22	0.05	0.71	0.07	0.16	0.08
GE1481184	NM_183391.2	tumor necrosis factor (ligand) superfamily, member 18	tnfsf18	0.18	0.11	0.19	0.11	0.13	0.05	0.16	0.12	0.64	0.19	0.16	0.08
GE33446	NM_007522.1	Bcl-associated death promoter	bad	1.71	0.29	1.61	0.11	1.35	0.13	1.65	0.20	1.74	0.11	1.70	0.36
GE33447	NM_007523.1	BCL2-antagonist/killer 1	bak1	2.50	0.08	3.01	0.28	2.76	0.21	3.00	0.44	2.84	0.18	2.29	0.13
GE34777	NM_007527.2	Bcl2-associated X protein	bax	9.61	0.60	12.40	1.35	8.31	0.21	13.73	2.05	21.01	0.91	11.14	1.97
GE1492802	NM_172729.1	caspase recruitment domain 4	card4	3.94	0.56	2.76	0.32	3.23	0.06	4.23	0.40	2.33	0.43	3.34	0.27
GE102998	AK002346.1	caspase recruitment domain family, member 11	card11	0.11	0.03	0.06	0.01	0.10	0.03	0.21	0.09	0.14	0.05	0.15	0.09
GE35156	NM_009807.1	caspase 1	casp1	2.62	1.27	1.74	0.16	1.80	0.03	4.04	1.06	2.61	0.40	3.27	1.02
GE41643	AK079804.1	predicted gene, 20257	gm20257	7.13	0.73	5.94	0.51	6.02	0.30	6.67	1.13	4.73	0.85	5.68	1.51
GE34811	NM_007611.1	caspase 7	casp7	5.90	0.57	5.33	0.51	5.49	0.41	5.51	0.43	3.98	0.18	4.75	0.45
GE35158	NM_009811.2	caspase 6	casp6	18.70	2.57	16.23	1.28	14.72	1.13	17.88	1.76	13.55	0.50	13.69	1.70
GE112035	NM_009809.2	caspase 14	casp14	0.62	0.05	0.60	0.07	0.57	0.10	0.83	0.03	0.65	0.05	0.62	0.05
GE35154	NM_009808.2	caspase 12	casp12	11.01	5.13	8.79	0.59	7.67	0.52	9.86	3.02	8.34	0.61	7.90	2.28
GE1543198	AK035300.1	NLR family, CARD domain containing 4	nlrc4	0.38	0.17	0.24	0.06	0.26	0.05	0.37	0.09	0.36	0.02	0.29	0.06
GE33470	NM_007609.1	caspase 4, apoptosis-related cysteine protease	casp4	1.08	0.59	0.73	0.00	0.75	0.05	1.25	0.29	1.24	0.14	1.19	0.29
GE42275	NM_130886.2	caspase recruitment domain family, member 14	card14	1.46	0.37	1.87	0.38	2.58	0.35	2.20	0.57	2.21	0.17	2.92	0.61
GE35157	NM_009810.1	caspase 3, apoptosis related cysteine protease	casp3	2.25	0.68	2.18	0.12	1.90	0.13	1.60	0.41	1.63	0.16	1.48	0.30
GE124534	NM_028844.1	apoptosis, caspase activation inhibitor	aven	4.02	0.64	4.19	0.15	3.81	0.40	4.57	0.28	4.71	0.12	4.24	0.15
GE42274	NM_130859.2	caspase recruitment domain family, member 10	card10	3.96	0.10	3.41	0.48	3.31	0.31	3.02	0.23	3.16	0.29	2.57	0.38
GE1569919	NM_175362.1	caspase recruitment domain family, member 11	card11	0.20	0.04	0.23	0.02	0.21	0.04	0.19	0.12	0.20	0.09	0.21	0.02
GE42472	NM_007610.1	caspase 2	casp2	7.72	1.94	8.84	0.81	7.01	0.37	6.86	1.11	7.71	0.60	5.48	0.62
GE35254	NM_009950.1	CASP2 and RIPK1 domain containing adaptor with death domain	cradd	8.90	1.44	7.02	0.46	6.71	0.56	11.18	0.23	8.13	0.24	7.99	0.40
GE35383	NM_010175.2	Fas (TNFRSF6)-associated via death domain	fadd	5.80	0.58	5.86	0.23	5.51	0.28	6.51	0.87	7.98	0.76	5.27	0.76
GE117206	NM_009594.1	v-abl Abelson murine leukemia oncogene 1	abl1	7.16	0.49	7.39	0.22	7.03	0.53	7.56	1.57	6.28	0.68	6.44	0.50
GE35109	NM_009740.1	B-cell leukemia/lymphoma 10	bcl10	15.89	1.69	16.14	0.36	13.88	0.58	14.18	1.72	15.10	0.15	13.00	0.57
GE1527640	AK031208.1	BCL2/adenovirus E1B interacting protein 1	Bnip1	1.19	0.04	1.25	0.18	1.11	0.05	1.02	0.14	1.14	0.11	1.12	0.10
GE42743	NM_016787.2	BCL2/adenovirus E1B 19kDa-interacting protein 1, NIP2	bnip2	40.03	4.44	32.74	1.86	32.13	1.28	40.65	2.36	35.71	3.21	37.48	3.03
GE35123	NM_009760.2	BCL2/adenovirus E1B 19kDa-interacting protein 1, NIP3	bnip3	37.57	8.86	30.90	0.79	31.06	3.23	51.54	1.29	47.15	2.00	43.99	0.68
GE42677	NM_011632.1	Tnf receptor-associated factor 3	traf3	5.04	0.26	6.97	0.22	5.17	0.72	4.44	0.58	7.93	0.19	4.18	0.55

Supplementary table 10. Cont'd….

Substance	ACCN#	Description	Gene Symbol	(Gene expression) 2 weeks						(Gene expression) 3 weeks					
				CTL		Hom-CKO		Het-CKO		CTL		Hom-CKO		Het-CKO	
				Average	SD	Average	SD	Average	SD	Average	SD	Average	SD	Average	SD
GE35054	NM_009652.1	thymoma viral proto-oncogene 1	akt1	15.11	0.71	15.15	0.63	12.93	0.49	16.44	1.49	12.86	1.77	13.97	0.93
GE36558	NM_013863.3	Bcl2-associated athanogene 3	bag3	8.78	2.51	10.11	0.72	9.86	0.48	12.63	3.34	11.33	0.57	10.38	1.82
GE39499	NM_026121.1	BCL2-associated athanogene 4	bag4	1.20	0.22	1.24	0.16	1.05	0.04	1.16	0.06	1.34	0.04	1.05	0.06
GE42603	NM_009736.1	Bcl2-associated athanogene 1	bag1	23.93	6.78	20.17	0.62	21.37	0.53	30.15	5.39	21.61	1.48	23.78	4.73
GE35110	NM_009741.2	B-cell leukemia/lymphoma 2	bcl2	4.07	0.77	5.24	0.96	3.42	0.23	2.95	0.13	4.75	0.47	2.75	0.06
GE117659	NM_007534.1	B-cell leukemia/lymphoma 2 related protein A1b	bcl2a1b	2.40	1.05	2.59	0.13	1.68	0.22	3.36	0.73	7.95	2.31	2.36	0.70
GE119649	NM_013479.1	Bcl2-like 10	bcl2l10	0.16	0.06	0.18	0.05	0.17	0.05	0.16	0.16	0.15	0.05	0.15	0.10
GE34779	NM_007537.1	Bcl2-like 2	bcl2l2	17.76	0.99	16.01	0.51	16.89	1.71	20.90	1.69	14.98	0.75	20.32	0.22
GE37790	NM_007566.1	baculoviral IAP repeat-containing 6	birc6	5.52	1.05	5.52	0.13	5.43	0.69	4.83	0.34	4.36	0.54	4.39	0.79
GE34749	NM_007464.1	baculoviral IAP repeat-containing 3	birc3	1.92	0.21	1.91	0.14	1.76	0.03	2.31	0.14	2.47	0.08	1.80	0.08
GE35076	NM_009688.1	baculoviral IAP repeat-containing 4	birc4	1.12	0.09	1.04	0.10	0.98	0.05	1.00	0.04	0.68	0.14	0.82	0.14
GE39382	NM_029653.1	death associated protein kinase 1	dapk1	0.80	0.02	0.91	0.07	0.74	0.09	1.00	0.21	0.91	0.03	0.79	0.08
GE33248	NM_010513.1	insulin-like growth factor I receptor	igf1r	1.57	0.33	1.75	0.44	1.45	0.06	1.29	0.11	1.82	0.39	1.28	0.02
GE33996	NM_008562.2	myeloid cell leukemia sequence 1	mcl1	24.84	2.36	21.99	3.29	23.81	0.91	29.28	5.15	24.62	1.53	26.17	2.75
GE129186	NM_138952.2	receptor (TNFRSF)-interacting serine-threonine kinase 2	ripk2	2.64	0.25	2.98	0.16	2.46	0.13	3.01	0.21	3.29	0.33	2.49	0.42
GE34075	NM_008670.1	baculoviral IAP repeat-containing 1a	birc1a	0.92	0.18	0.90	0.18	0.84	0.01	0.78	0.11	0.98	0.10	1.11	0.17
GE119127	NM_010870.1	baculoviral IAP repeat-containing 1e	birc1e	40.67	7.72	30.64	2.01	30.79	2.92	37.28	1.67	29.41	1.97	33.05	3.22
GE35781	NM_010872.1	baculoviral IAP repeat-containing 1b	birc1b	0.40	0.13	0.39	0.12	0.50	0.07	0.68	0.10	0.64	0.07	0.53	0.05
